# Design, Synthesis, and Selective Antiproliferative Activity of Indolizine Derivatives as Microtubule Destabilizers

**DOI:** 10.1002/ardp.70161

**Published:** 2025-12-03

**Authors:** Victor Hugo Catricala Fernandes, Maitê Bueno Giometti, Franco Jazon Caires, Gabriel de Paula Bueno, Gabriel da Silva, Andréia Machado Leopoldino, Anna Junker, Giuliano Cesar Clososki

**Affiliations:** ^1^ Department of Biomolecular Sciences, Faculty of Pharmaceutical Sciences of Ribeirão Preto University of Sao Paulo Ribeirão Preto Brazil; ^2^ Werner Siemens Imaging Center, Department of Preclinical Imaging and Radiopharmacy, Cluster of Excellence iFIT (EXC 2180) “Image‐guided and Functionally Instructed Tumor Therapies” University of Tuebingen Tuebingen Germany; ^3^ Department of Clinical Analyses, Toxicological and Food Sciences, Faculty of Pharmaceutical Sciences of Ribeirão Preto University of Sao Paulo Ribeirão Preto Brazil

**Keywords:** antiproliferative, bioisosteric exchange, indolizine, molecular docking, tubulin inhibitor

## Abstract

The development of selective anticancer agents with minimal off‐target toxicity remains a major therapeutic goal. In this study, we synthesized and evaluated a series of 32 indolizine derivatives for antiproliferative activity against oral (CAL‐27), breast (BT‐20), and gastric (HGC‐27) cancer cell lines, as well as non‐tumoral fibroblasts (OHMF). Compounds **8e** and **8h** emerged as potent and selective candidates, exhibiting nanomolar IC₅₀ values (47–117 nM) and negligible cytotoxicity toward healthy cells. These compounds induced G2/M cell‐cycle arrest, inhibited tubulin polymerization, and modulated proteins related to apoptosis and proliferation, including p‐AKT, cyclin D1, Bcl‐2, and p21. Docking studies confirmed their interaction with the colchicine‐binding site of tubulin. Together, the results support further investigation of these compounds as microtubule‐interacting agents with selective antiproliferative activity.

## Graphical abstract

A series of indolizine derivatives was synthesized and biologically evaluated. Compounds **8e** and **8h** showed nanomolar antiproliferative activity against cancer cells and low toxicity to normal cells. They act as microtubule‐destabilizing agents, inducing G2/M arrest and modulating key proteins. Docking confirmed binding at the colchicine site of tubulin.

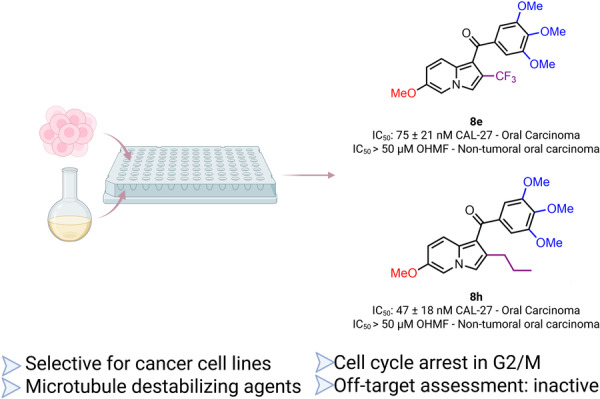

## 1 Introduction

Cancer remains a leading cause of death worldwide, with an estimated 2,041,910 new cases and 618,120 deaths projected in the United States for 2025. While mortality rates have declined since 1991, preventing nearly 4.5 million deaths due to reduced smoking, earlier diagnoses, and improved treatments [1]. The absolute number of cancer deaths continues to rise, primarily due to population aging, highlighting the persistent need for the development of more efficient and targeted anticancer therapies [2].

In the search for new druggable cancer targets, phenotypic and targeted approaches coexist, each offering distinct advantages and limitations [3, 4]. Phenotypic screening enables the unbiased discovery of compounds based on observable effects in cells, making it particularly valuable for identifying novel mechanisms in complex or poorly understood cancers. However, this approach can be limited by challenges in pinpointing the exact molecular targets, lower throughput, and reproducibility issues [4, 5]. Targeted approaches, by contrast, focus on known cancer‐related proteins, enabling rational drug design, high‐throughput screening, and the development of precision therapies and biomarkers. Nevertheless, they may miss broader biological contexts and are vulnerable to resistance as cancer cells adapt or find alternative pathways [6, 7]. Combining both strategies can leverage their complementary strengths and lead to more effective and innovative cancer treatments [7, 8].

**Figure 1:**
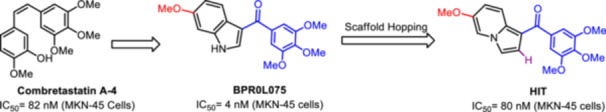
Scaffold‐hopping strategy leading to the indolizine hit compound (**HIT**).

Natural product‐derived drugs have long been central to cancer therapy, forming the foundation of many successful chemotherapeutics such as paclitaxel, vincristine, and doxorubicin [9]. With their unique structural complexity and potent biological activity, these compounds remain a rich source of new anticancer agents, particularly for treating tumors that are resistant or difficult to treat. One notable example is combretastatin A‐4, a natural product initially discovered through phenotypic screening based on its antiproliferative effects. Its mode of action was later identified as an inhibition of tubulin polymerization, disrupting microtubule dynamics and cell division [10]. Although combretastatin A‐4 (Figure 1) advanced to clinical trials, it was ultimately withdrawn due to unsuitable pharmacokinetic properties. Nevertheless, several research groups have since explored this class of compounds to improve both the activity profile and solubility of tubulin polymerization inhibitors [11, 12]. One of the advancements was the development of the indole derivative **BPR0L075** (Figure 1), which features a substitution pattern similar to that of combretastatin A‐4. This compound exhibited over a 10‐fold improvement in antiproliferative activity against MKN‐45 (gastric carcinoma) cells, accompanied by improved solubility. Additionally, **BPR0L075** demonstrated growth inhibitory activity against a stomach carcinoma cell line (NUGC3), where combretastatin A‐4 showed no appreciable effect in comparison [13, 14]. Based on this indole derivative, a study on “scaffold hopping” was conducted, a medicinal chemistry strategy in which the central core is replaced with different cores in an attempt to improve the activity/affinity, metabolic stability profile, or alter physicochemical attributes and ADMET properties. Through this method, it was found that replacing the indole ring of compound **BPR0L075** with the indolizine ring, as in compound **HIT** was feasible; however, the antiproliferative activity of the indolizine compound was lower than that **BPR0L075** [15]. However, this study only replaced the indole core with the indolizine, and the influences of the substituents were not investigated. Further studies have shown that the trimethoxyphenyl (TMP) group is commonly found in tubulin inhibitors and derivatives of Combretastatin A‐4. However, it is not considered essential for activity, opening opportunities for further investigation into alternative substituents on the acyl‐benzene moiety to assess their impact on antiproliferative activity [16]. In this study, we aimed to improve the antiproliferative activity of a previously identified **HIT** compound (Figure 1) by evaluating the effects of modifying the acyl‐benzene moiety and the methoxy group at the 6‐position of the indolizine core. We also explored substitutions at the 2‐position, which had not been properly addressed before, to identify key substituents. These modifications enabled the preparation of a new library of compounds with enhanced antiproliferative activity in the cell lines tested and allowed us to confirm the mechanism of action as tubulin polymerization inhibitors and other biochemical evaluations of our lead compound.

## 2 Results and Discussion

We designed the initial series of novel indolizine‐based compounds with a focus on understanding the impact of the aryl‐ketone moiety in combination with a methoxy group at the 6‐position of the indolizine ring, incorporating both electron‐withdrawing and electron‐donating groups to examine their inductive and mesomeric effects on antiproliferative activity.

**Scheme 1:**
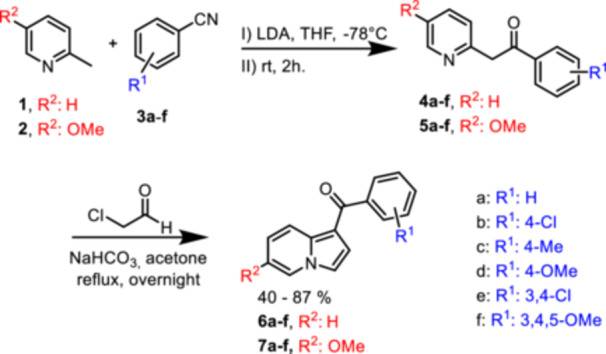
Synthesis of intermediates **3a–f**, **4a–f** and **5a–f**, and different indolizines **6a–f** and **7a–f**.

Starting from 2‐methylpyridine (**1**) or synthesized 5‐methoxy‐2‐methylpyridine (**2**), direct metalation with LDA was employed, followed by the addition of different previously prepared benzonitrile derivatives (**3a–3f**) to form the intermediates **4a–4f** (R^2^: H) and **5a–7f** (R^2^: OMe, Scheme 1). Intermediates containing a methoxy substituent at position C‐6 in the indolizine core were not isolated and were used without purification, as they exhibited sufficient purity after the workup. After the preparation of the intermediates, the cyclization step involved the use of chloroacetaldehyde in a slightly basic medium with sodium bicarbonate in acetone, yielding the desired indolizines **6a–6f** (R^2^: H) and **7a–7e** and the initial lead structure **HIT** (**7f**, R^2^: OMe) with yields ranging from 40% to 87%.

This initial set of compounds provided a preliminary guide toward understanding key structural features required for activity. Variations at the 6‐position of the indolizine core (presence or absence of a methoxy group), along with modifications in the aryl substituents, allowed us to evaluate how electronic and lipophilic changes impact antiproliferative activity.

The compounds **6a–f** and **7a–f** were submitted to a phenotypic screening of the antiproliferative activity against BT‐20 (breast carcinoma), HCG‐27 (gastric carcinoma), and CAL‐27 (oral carcinoma) cell lines. The compounds were tested at a concentration of 50 µM, administered for 72 h (Table 1) alongside paclitaxel as a positive control at a concentration of 2 μM. The intermediates **4a–4f** were also evaluated; however, they did not exhibit any significant antiproliferative activity (see Supporting Information S1: Figure S1).

**Table 1:** Cellular growth inhibition in % of different cell lines at 50 μM of **6a–f** and **7a–f**.

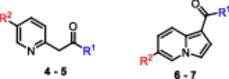					
			% Cellular growth inhibition (at 50 µM)^d^		
			Mean ± SD		
Compound	R^2^	R^1^	CAL‐27^b^	BT‐20^c^	HGC‐27^a^
**4f**	H	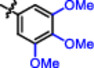	20 ± 2.2	19 ± 1.2	10 ± 0.7
**5f**	OMe	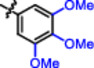	67 ± 2.7	52 ± 4.2	48 ± 4.9
**6a**	H	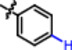	15 ± 0.8	26 ± 1.0	20 ± 1.1
**6b**		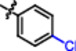	25 ± 0.4	32 ± 0.8	26 ± 1.0
**6c**		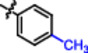	22 ± 2.6	28 ± 1.3	20 ± 0.6
**6d**		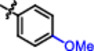	18 ± 0.6	28 ± 0.5	24 ± 1.1
**6e**		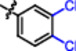	42 ± 0.8	40 ± 0.4	41 ± 1.0
**6f**		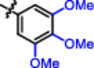	57 ± 0.9	43 ± 0.5	38 ± 1.6
**7a**	OMe	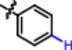	20 ± 0.9	20 ± 0.4	18 ± 0.5
**7b**		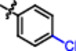	23 ± 1.0	8 ± 1.3	10 ± 1.4
**7c**		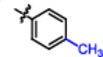	20 ± 0.7	15 ± 0.5	13 ± 0.7
**7d**		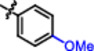	15 ± 1.1	7 ± 0.9	1 ± 1.0
**7e**		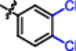	70 ± 3.8	53 ± 1.8	68 ± 3.4
**7f (HIT)**		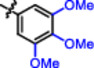	58 ± 1.3	43 ± 0.8	43 ± 0.7
Paclitaxel (2 μM)—positive control			54 ± 1.2	50 ± 1.0	46 ± 1.8

^a^ HGC‐27 (gastric carcinoma);

^b^ CAL‐27 (oral carcinoma);

^c^ BT‐20 (breast carcinoma);

^d^ Determination of % Cellular growth inhibition was made in triplicate (*n* = 3).

The intermediates **5a–5e** were not assessed. However, compound **5f** was tested and showed a marked increase in antiproliferative activity compared with **4f**, suggesting that the methoxy group plays an important role in the observed activity. These non‐cyclized intermediates **4a–f** and **5a–f** were not subjected to further investigation due to the possibility of tautomerization (as observed by the NMR analysis) that could lead to chemical instability, potential assay interference, and metabolic liability [17].

Among the indolizines tested, derivatives lacking a methoxy group at the 6‐position exhibited similar or slightly lower potency in antiproliferative activity compared with compounds with methoxy groups at this position. This suggests that the methoxy group may be beneficial for activity in some specific cases, as compound **6f**, lacking the substituent, still exhibits comparable levels of cell growth inhibition to compound **7f**, which bears the methoxy group at the 6‐position. However, the lower activity observed for compound **6e** in comparison to **7e** indicates that, in certain cases, the presence of the methoxy group at the 6‐position may be beneficial for antiproliferative activity.

While compounds **6f**, **7e**, and **7f** exhibited the highest antiproliferative potential, it is worth noting that our anticipated 70% inhibition threshold in initial screenings was not met, with the best result hovering around 60% inhibition. The initial screening indicates that the TMP group could be successfully replaced by a dichloro substitution pattern, especially when combined with the methoxy group at the 6‐position of the indolizine core. Lack of substituents, as in compounds **6a** and **7a**, as well as the presence of only one substituent at the phenyl ring, resulted in compounds with a weak inhibitory profile.

Given the structural similarities between our most active compounds and known tubulin inhibitors, particularly concerning the TMP substitution pattern, we hypothesized that these compounds may exert their effects through interference with tubulin dynamics. Tubulin is a well‐established target in anticancer therapy and inhibition of its polymerization disrupts mitotic spindle formation, ultimately leading to cell‐cycle arrest and apoptosis [18]. Therefore, to validate this hypothesis and gain insight into possible molecular interactions, we performed docking studies using the colchicine‐binding domain of tubulin (PDB ID: 4O2B) as the target binding site.

**Figure 2:**
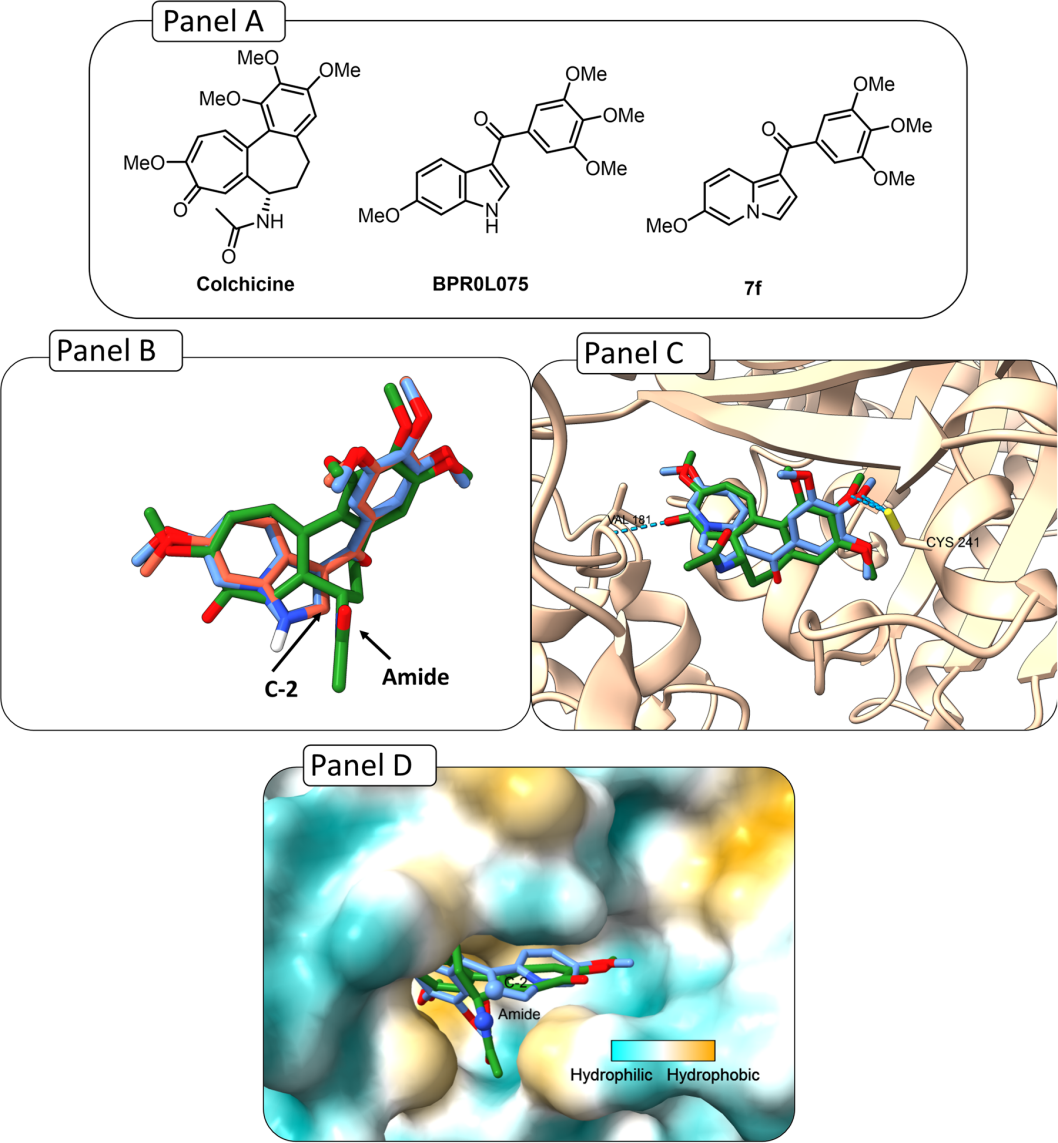
Docking interactions of the compounds **7f**, **BPR0L075**, and **colchicine** in the colchicine binding site of tubulin (PDB ID: **4O2B**). (Panel A) Structure of compound **7f**, **BPR0L075**, and colchicine. (Panel B) Superimposition of compound **7f** (in blue), colchicine (in green), and **BPR0L075** (in red). (Panel C) Interactions of the molecule **7f** (in blue) and colchicine (in green) within the Cys241 of the binding pocket. (**Panel D**) Hydrophobicity surface of docked compounds **7f** (in blue, C‐2 position highlighted), and colchicine (in orange, amide highlighted).

Compounds **7f** and **BPR0L075** were docked into the colchicine‐binding pocket and superimposed with colchicine. The TMP groups of both compounds overlapped well (Figure 2, Panel B), suggesting a binding mode similar to colchicine and, consequently, potential tubulin inhibition. Notably, **BPR0L075** has already been validated both in silico and in vitro, further supporting its role as a tubulin inhibitor [15]. The docked poses of compounds **7f** and **BPR0L075** exhibited nearly identical orientations within the colchicine‐binding site. Superimposition of the indole and indolizine scaffolds yielded an RMSD close to zero, further indicating highly similar interactions (Figure 2, Panel B). Finally, in the docked pose of compound **7f**, a hydrogen bond with Cys241 was observed, an interaction also presents for **BPR0L075** and colchicine (Figure 2, Panel C).

Furthermore, docking results revealed that the 2‐position of the indolizine core lies within a pocket suitable for molecular expansion through the introduction of new fragments, potentially enabling additional interactions, such as hydrophobic contacts. (Figure 2, Panel D). Based on this observation, we initiated a SAR study derived from compound **7f** by introducing substituents at this position. It was proposed that these substituents could occupy the same region as the amide group of colchicine (Figure 2, Panel D). To explore this possibility, compounds bearing substituents at the 2‐position were designed, including an ester and an acetamide, in an attempt to mimic the amide group of colchicine (Figure 2, Panel D).

**Scheme 2:**
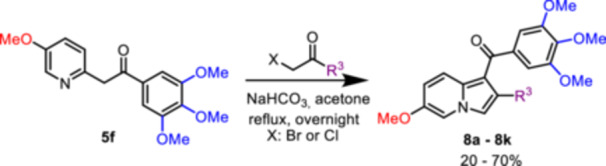
Synthesis of indolizine derivatives **8a**–**8k**.

The second series of indolizine derivatives **8a–8k** was prepared starting from the intermediate **5f** using different α‐haloketones with the desired substitution pattern (Scheme 2).

The compounds were subjected to the phenotypic assay of their antiproliferative activity at a concentration of 50 μM. The results (Table 2) revealed an increase in activity upon the introduction of more hydrophobic groups into the structure.

**Table 2:** Cellular growth inhibition of different cell lines at 50 μM of and **8a–8k**.

		% Cellular growth inhibition (at 50 µM)^d^		
		Mean ± SD		
Compound	R^3^	CAL‐27^b^	BT‐20^c^	HGC‐27^a^
**8a**	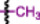	64 ± 2.7	52 ± 5.0	41 ± 4.3
**8b**	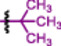	84 ± 3.2	62 ± 0.5	46 ± 6.0
**8c**	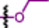	56 ± 4.6	55 ± 4.7	43 ± 3.5
**8d**	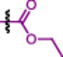	66 ± 0.8	55 ± 4.7	44 ± 1.9
**8e**	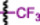	76 ± 1.3	62 ± 2.3	53 ± 2.2
**8f**	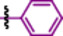	69 ± 0.4	55 ± 1.8	49 ± 4.0
**8g**	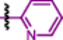	68 ± 1.0	54 ± 2.8	44 ± 0.4
**8h**	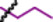	90 ± 1.8	71 ± 2.8	70 ± 3.3
**8i**	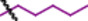	57 ± 2.5	38 ± 3.5	61 ± 1.5
**8j**	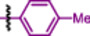	73 ± 4.0	61 ± 2.0	71 ± 2.0
**8k**	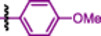	73 ± 4.5	64 ± 1.5	74 ± 2.0
Paclitaxel (2 μM)		54 ± 1.2	50 ± 1.0	46 ± 1.8

^a^ HGC‐27 (gastric carcinoma);

^b^ CAL‐27 (oral carcinoma);

^c^ BT‐20 (breast carcinoma);

^d^ Determination of % Cellular growth inhibition was determined in triplicate (*n* = 3).

As suggested by molecular docking results, the functionalization of the 2‐position by introducing an amide group would be an interesting modification; however, synthetic challenges prevented us from accomplishing this transformation (Figure 2, Panel D). As an alternative, to mimic this functionality, an analog bearing an ethyl ester was designed, since this functional group can emulate the amide by maintaining a similar carbonyl‐based hydrogen bond acceptor capability and preserving the geometry.

Compound **8d**, bearing an ethyl ester, exhibited antiproliferative activity but did not surpass the 70% inhibition threshold. Interestingly, compounds containing **8b** (R^3^: *tert*‐butyl), **8e** (R^3^: CF₃), **8h** (R^3^: *n*‐propyl), **8j** (R^3^: tolyl), and **8k** (R^3^: anisole) substituents showed higher levels of inhibition, ranging from 89.5% to 73.1%. These groups share a common feature: their lipophilic character, which aligns with our docking results, indicating that the indolizine core is positioned within a lipophilic pocket of the target protein, thereby favoring stronger interactions with lipophilic substituents. Compounds that exceeded the 70% inhibition threshold were selected for IC_50_ determination. Additionally, all selected compounds were tested against nontumor human fibroblasts (OHMF) to assess selectivity and potential cytotoxicity toward healthy cells.

Thus, compounds **8b** (R^3^: *tert*‐butyl), **8e** (R^3^: CF₃), **8h** (R^3^: *n*‐propyl), **8j** (R^3^: tolyl), and **8k** (R^3^: anisole) were selected for IC_50_ determination. We also determined the IC_50_ value of compound **7e**, synthesized in the first series with a 3,4‐dichloro group at R², to confirm whether this substitution could effectively replace the TMP group while maintaining antiproliferative activity. Compound **7f** was used as our positive control due to its known inhibitory activity against various cancer cell lines and its effect on α‐tubulin. However, its performance against the specific cell lines used in this study was previously unknown (Table 3).

**Table 3:** IC_50_‐values in nM of the selected compounds on different cell lines.

	IC_50_ (nM)^a^			
	Mean ± SD			
Compound	HGC‐27^b^	CAL‐27^c^	BT‐20^d^	OHMF^e^
**7f (HIT)**	475 ± 105	105 ± 21	111 ± 28	> 50,000
**7e**	11,860 ± 1,180	12,350 ± 353	14,095 ± 1,138	> 50,000
**8a**	410 ± 212	325 ± 92	370 ± 28	> 50,000
**8e**	117 ± 32	75 ± 21	105 ± 49	> 50,000
**8h**	75 ± 35	47 ± 18	90 ± 56	> 50,000
**8j**	465 ± 35	300 ± 85	465 ± 35	> 50,000
**8k**	880 ± 14	690 ± 156	800 ± 127	> 50,000
**BPR0L075**	5 ± 1.4	2 ± 0.1	4 ± 1.1	—
Verubulin	3 ± 0.4	2 ± 0.3	5.0 ± 0.7	—
Paclitaxel	48 ± 16	3 ± 0.6	45 ± 7	—

^a^ Determination of IC_50_ was made in triplicate (*n* = 3);

b HGC‐27 (gastric carcinoma);

c CAL‐27 (oral carcinoma);

d BT‐20 (breast carcinoma);

e OHMF (non‐tumoral oral fibroblast).

All IC₅₀ values obtained in our study were in the micromolar to high nanomolar range (above 100 nM), except for compounds **8e** and **8h**, which demonstrated moderate nanomolar potency (47–117 nM), indicating stronger antiproliferative effects. The HIT compound **7f** remained in the higher nanomolar range when tested on the selected cancer cell lines. This aligns well with the reported IC_50_‐value of 80 nM for compound **7f** when tested on MKN‐45 gastric cancer cells [15]. On HGC‐27 cell line, the compound was, however, around fourfold less potent. This discrepancy is most likely due to differences between the cell lines used. Although both MKN‐45 and HGC‐27 are derived from gastric carcinoma, they possess distinct molecular and phenotypic profiles. MKN‐45 cells originate from a gastric adenocarcinoma and are characterized by high proliferation rates and greater chemosensitivity, while HGC‐27 cells derive from an undifferentiated gastric carcinoma and exhibit mutations associated with chemoresistance, along with enhanced migratory and invasive behavior [19, 20, 21]. Such genetic and functional differences likely impact drug uptake, efflux, and overall sensitivity, resulting in the higher IC_50_ value observed for the HGC‐27 cell line.

Compound **7e**, which bears a 3,4‐dichloro substitution at the R² position and slightly outperformed compound **7f** at the concentration of 50 µM, showed lower potency compared with **7f** in the full concentration‐response range, supporting the notion that the TMP group is more favorable for antitumor activity.

In all cancer cell lines tested, both compounds **8e** and **8h** exhibited lower IC_50_ values than the reference compound **7f** (**HIT**), indicating greater potency and supporting their potential use as lead compounds in further studies. In the HGC‐27 gastric cancer cell line, the improvement was particularly significant, with approximately a four‐ to sixfold decrease in IC_50_ values. In the CAL‐27 and BT‐20 cell lines, **8e** and **8h** showed no enhanced inhibitory activity compared with **7f**.

It is also worth noting that all compounds were evaluated for their growth‐inhibitory effects on the healthy fibroblast cell line OHMF. All compounds exhibited IC_50_ values higher than 50 µM. These results indicate a favorable selectivity toward tumor cell lines and low cytotoxicity to normal cells, underscoring the therapeutic potential of these indolizine derivatives.

The identified promising compounds were evaluated in a tubulin polymerization inhibition assay alongside paclitaxel and verubulin as reference compounds (Table 4). The IC_50_ values obtained for the tested compounds were very similar to those of paclitaxel. These results suggest that our compounds exhibit comparable tubulin inhibitory activity to paclitaxel. In contrast, verubulin, a known microtubule destabilizer, showed a significantly lower IC_50_ value, approximately three times lower than those of our compounds. The IC_50_ of verubulin is consistent with previously reported data in the literature, which demonstrated an IC50 value of 3.2* ±* 0.7 µM for tubulin destabilization [22].

**Table 4:** IC_50_ values determined in the tubulin inhibition assay.

Compound	IC_50_ (µM)^a^
**7f (HIT)**	14.8
**8e**	9.3
**8h**	10.9
Verubulin	3.2
Paclitaxel	8.2

^a^ Determination of IC_50_ was made in duplicate (*n* = 2).

**Figure 3:**
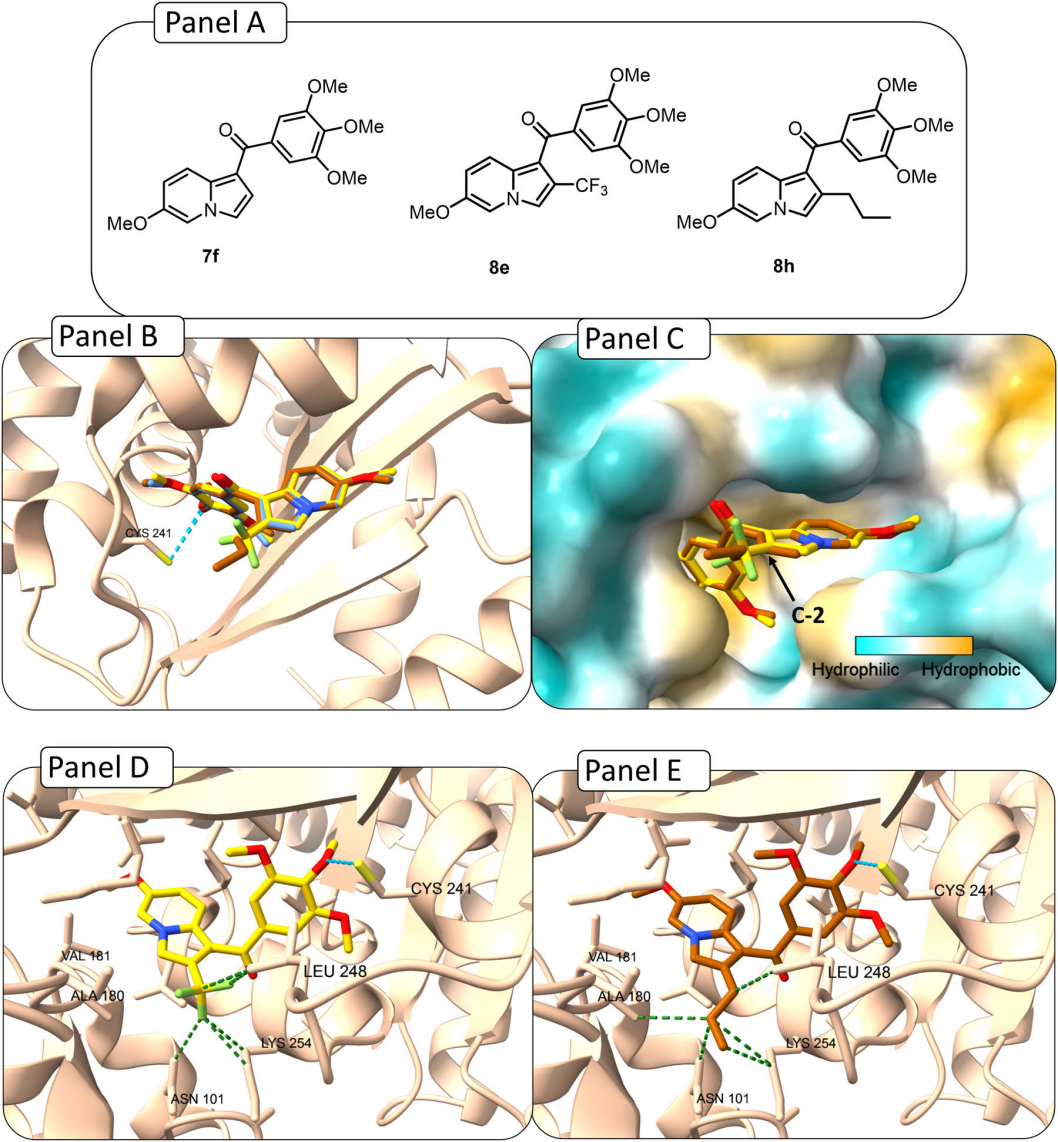
Docking interactions of the compounds **7f**, **8e**, and **8h** in the colchicine binding site of tubulin (PDB ID: **4O2B**). (Panel A) Structure of compounds **7f**, **8e**, and **8h**. (Panel B) Superimposition of compounds **7f** (in blue), **8e** (in yellow), and **8h** (in the dark orange). (Panel C) Hydrophobicity surface of docked compounds **7f** (in blue), **8e** (in yellow), and **8h** (in the dark orange). (Panel D) Interactions of the molecule **8e** (in yellow) within the Cys241 of the binding pocket, and also hydrophobic contacts with Leu248, Lys 254, and Asn101 Panel E) Interactions of the molecule **8h** (in the dark orange) within the Cys241 of the binding pocket, and also hydrophobic contacts with Leu248, Lys254, Ala180, and Asn101.

Compounds **7f**, **8e**, and **8h** (Figure 3, Panel A) were also docked into tubulin (PDB ID: 4O2B). The docked poses of compounds **8e** and **8h** were similar to that when compared with compound **7f** (our HIT compound), suggesting that these molecules may interact with tubulin in a comparable manner (Figure 3, Panel B).

Our biological results, together with the docking simulations of compounds **8e** and **8h**, confirmed that more hydrophobic groups fit well into this pocket, which had already been shown to be favorable for such substituents (Figure 3, Panel C). Furthermore, both **8e** and **8h** were found to preserve the hydrogen bond with Cys241 and, in addition, established new hydrophobic contacts with Leu248, Lys254, and Asn101, and one additional interaction in **8h** with Ala180. The hydrophobic interactions were not observed for compound **7f**, which could explain the improvement in the activity of **8e** and **8h**.

**Figure 4:**
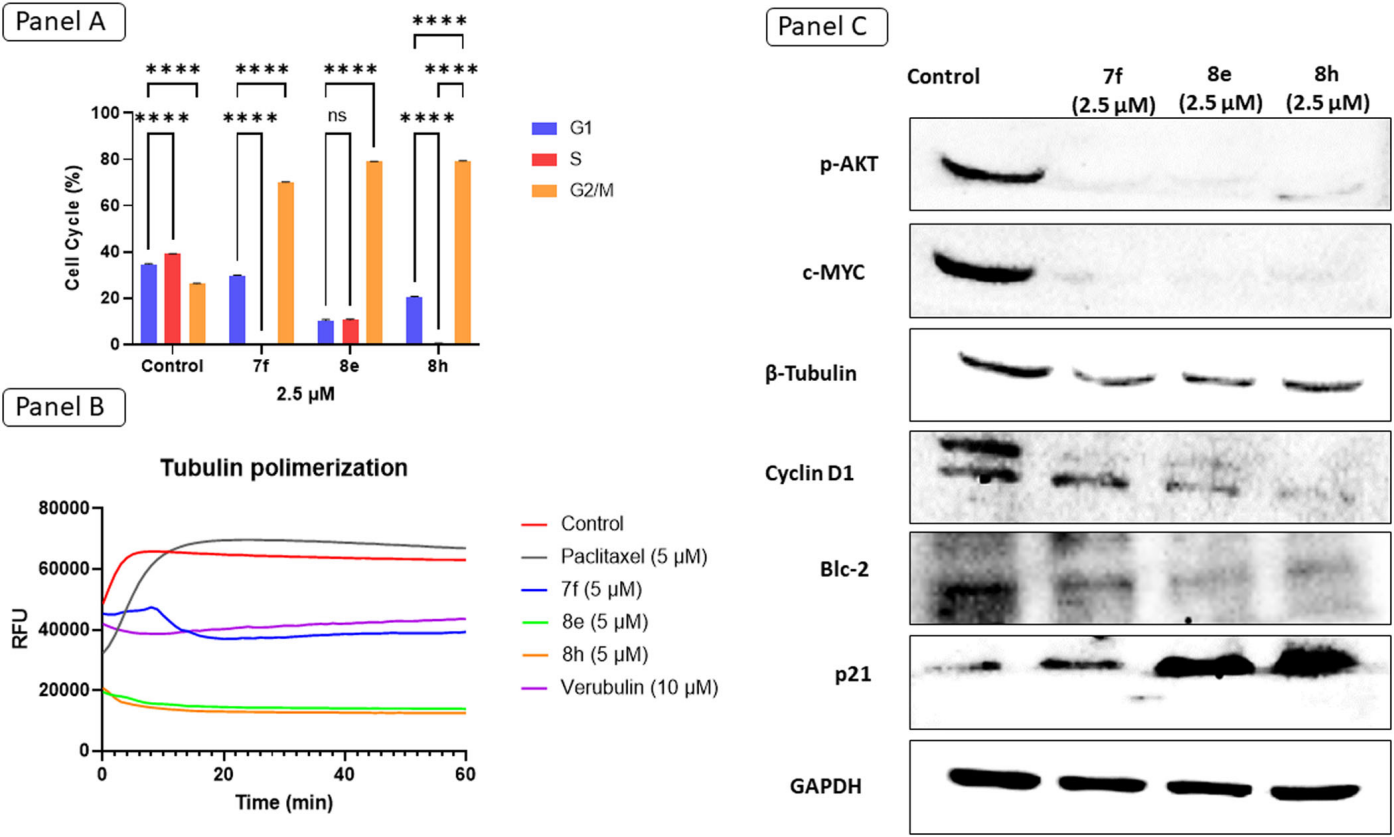
Antiproliferative effects of compounds **7f**, **8e**, and **8h** on BT‐20 breast cancer cells. (Panel A) Cell‐cycle distribution after treatment with compounds (2.5 µM for 24 h) and vehicle control, showing G1, S, and G2/M phases. Statistical analysis was performed using two‐way ANOVA followed by Tukey's posttest. The indicated statistical differences refer to comparisons within the same treatment (****p < 0.001). (Panel B) Tubulin polymerization assay performed with compounds (5 µM), compared with control (without treatment) and paclitaxel. (Panel C) Western blot analysis of key proteins involved in cell‐cycle regulation (Cyclin D1, p21), apoptosis (Bcl‐2), proliferation (c‐MYC), and signaling (p‐AKT), as well as β‐Tubulin and GAPDH as primary antibody controls, following treatment with **7f**, **8e**, and **8h** (2.5 µM for 24 h).

To investigate the mechanism of action, the most promising compounds, **7f**, **8e**, and **8h**, were selected for further studies. We first assessed their effects on the cell cycle, since microtubule‐targeting agents typically lead to mitotic arrest. BT‐20 breast cancer cells were treated with the compounds at a concentration of 2.5 µM, and cell‐cycle distribution was analyzed by flow cytometry (Figure 4, Panel A). All three compounds induced a significant accumulation of cells in the G2/M phase, indicating a cell‐cycle arrest at this checkpoint. This behavior is consistent with the known effects of tubulin inhibitors, which disrupt spindle formation during mitosis and prevent proper chromosome segregation [23]. The G2/M arrest observed is consistent with the antiproliferative effects noted in breast, gastric, and oral carcinoma cells, supporting a mechanism involving microtubule disruption.

Next, to directly confirm the compounds' interaction with tubulin, we performed a tubulin polymerization assay, which tracks the assembly of microtubules in vitro by measuring fluorescence changing over time. All compounds exhibited tubulin‐destabilizing activity, with **8e** and **8h** being the most potent (Figure 4, Panel B). Their polymerization curves showed significantly lower relative fluorescence units (RFUs) compared with both the negative control and paclitaxel, a known microtubule‐stabilizing agent. While paclitaxel promoted rapid and sustained polymerization, the test compounds inhibited microtubule formation over time, a hallmark of agents that destabilize microtubules. The cell‐cycle arrest in G2/M further supports this observation, as destabilization prevents proper spindle formation, leading to a block in cell division.

To further investigate the downstream effects, we analyzed the expression of proteins involved in cell‐cycle control and apoptosis using Western blot analysis (Figure 4, Panel C). A reduction in phosphorylated AKT (p‐AKT) levels was observed for all three compounds, suggesting inhibition of the PI3K/AKT pathway, which is involved in promoting cell survival and cell‐cycle progression [24]. Additionally, expression of the oncogenic transcription factor c‐MYC was also reduced, indicating downregulation of proliferative signaling.

Focusing on regulators of cell‐cycle checkpoints, cyclin D1 levels were markedly decreased in cells treated with **8e** and **8h**, suggesting an additional interference with the G1/S transition. In parallel, Bcl‐2, a key antiapoptotic protein, was downregulated, supporting the activation of apoptotic mechanisms. In contrast, the expression of p21 was upregulated in response to treatment with **8e** and **8h**.

Moreover, while p21 is often considered cytoprotective in normal cells, its role in cancer cells is complex. Under certain stress conditions, such as microtubule destabilization, p21 can shift toward proapoptotic functions, particularly when antiapoptotic signals, like Bcl‐2, are suppressed [25]. Therefore, the concurrent increase in p21 expression and decrease in Bcl‐2 levels in cells treated with **8e** and **8h** suggest a cooperative mechanism, reinforcing both cell‐cycle arrest and apoptotic signaling. This dual activity strengthens the case for these compounds as effective antitumor agents, capable of not only halting cell proliferation but also triggering programmed cell death.

Given the observed effects on cell‐cycle regulation and apoptosis, we also explored the potential involvement of alternative pathways that may contribute to the compounds' antiproliferative activity. In particular, we focused on the P2X7 receptors, which are known to play roles in cancer progression and have been linked to cytoskeletal organization [26]. This investigation aimed to determine whether the observed biological effects were exclusive to microtubule disruption or if additional targets, such as purinergic receptors, might also be involved. Notably, the P2X7 receptor has been reported to interact with cytoskeletal components such as β‐tubulin, suggesting a potential functional link between microtubule dynamics and P2X7 receptor signaling [27].

All synthesized compounds were, therefore, assessed for off‐target activity against the P2X7 receptor. Despite the reported interplay, none of the synthesized compounds, including **8e** and **8h**, showed significant activity against the P2X7 receptor at 10 μM in the functional P2X7R YO‐PRO‐1 assay. These results indicate that the antiproliferative effects of **8e** and **8h** are primarily driven by microtubule destabilization and cell‐cycle arrest, rather than modulation of the P2X7 receptor pathway.

Due to its structural similarity to the P2X7 receptor, the P2X4 receptor was also selected for assessing off‐target activity. None of the compounds displayed antagonistic activity at a concentration of 10 μM in a functional Ca^2+^‐ion flux P2X4R assay, reinforcing the specificity of **8e** and **8h** towards microtubule interference.

## 3 Conclusions

In this study, we successfully designed, synthesized, and biologically evaluated a novel library of 30 indolizine‐based compounds, 28 of which are reported here for the first time. The synthetic strategy employed allowed for a modular and regioselective synthesis of the indolizine core, enabling systematic modifications at positions 1, 2, and 6. This approach was instrumental in constructing a focused SAR study, revealing the impact of structural variations on biological activity. The methoxy substituent at the 6‐position of the indolizine ring was found not to influence activity directly, but may provide a synergistic effect when combined with the TMP group. Finally, lipophilic and saturated substituents at position 2 of the indolizine ring tend to improve the overall activity profile of the compounds.

Through phenotypic assays, compounds **8e** and **8h** were identified as the most promising candidates, displaying nanomolar IC₅₀ values against oral (CAL‑27), breast (BT‑20), and gastric (HGC‑27) cancer cell lines, and minimal cytotoxicity in non‐tumoral fibroblasts (OHMF). Mechanistic investigations confirmed that these compounds act as microtubule‐destabilizing agents, inducing G2/M phase arrest and modulating key regulatory proteins involved in cell cycle (Cyclin D1, p21), survival (p‐AKT, c‐MYC), and apoptosis (Bcl‐2).

Docking studies further supported their binding to the colchicine‐binding site of tubulin, in line with the observed cellular effects. Notably, the hydrophobic substituents at position 2 of the indolizine ring played a crucial role in enhancing both tubulin affinity and cellular potency. Taken together, our findings validate the indolizine scaffold as a robust and tuneable platform for the development of selective antitumor agents. Compounds **8e** and **8h** stand out as lead candidates for further structural optimization and preclinical evaluation, with the potential to contribute to the next generation of microtubule‐targeting cancer therapies.

## 4 Experimental

### 4.1 Chemistry

#### 4.1.1 General

The reagents and solvents underwent prior purification, following established procedures. For instance, THF was treated with metallic sodium and benzophenone as an indicator and then refluxed for at least an hour before use. Reactions were monitored via TLC on silica gel 60 plates, pre‐coated with a UV light indicator from Sigma‐Aldrich. Product purification was carried out on Flash Silica (Sigma‐Aldrich, 230‐400 mesh, 40–63 μm), employing suitable solvent mixtures as eluents accordingly to specific procedures. Saturated solutions of ammonium chloride, sodium bicarbonate, sodium chloride, and sodium thiosulfate were employed in the preprepared liquid–liquid extraction process for various reactions. The precursor 2‐methyl‐pyridine (**1**) and benzonitrile (**3a**) were purchased from Sigma‐Aldrich, while the Verubulin control was synthesized following a previously established methodology from our research group [28].

The nuclear magnetic resonance spectra were acquired on Bruker Fourier300—Ultra shield, Bruker DRX‐400, or Bruker DRX‐500 spectrometers, operating at frequencies of 300 MHz, 400 MHz, and 500 MHz, respectively, for hydrogen. The chemical shift values (δ) presented are in parts per million (ppm). The signal multiplicity is given in parentheses, along with the coupling constant (J) in Hertz (Hz) and the number of hydrogens for each signal.

The equipment used for obtaining the chromatograms and low‐resolution mass spectra was a Shimadzu GC‐MS‐QP2010 gas chromatography‐mass spectrometry (GC‐MS) system (70 eV), employing a DB‐5‐MS column (J&W Scientific), with helium as the carrier gas at a flow rate of 1.30 mL/min and a pressure of 69.3 kPa. The obtained spectra assisted in monitoring reactions, verifying product formation. High‐resolution accurate mass–mass Spectrometry (HRAM‐MS) spectra were obtained using a Bruker Daltonics LC‐MS system, model MicroTOF QII, equipped with an electrospray ionization source and a time‐of‐flight (TOF) analyzer. Spectra were acquired via direct infusion in positive mode. For determining the melting point range (mp) of synthesized molecules, a Buchi B‐545 apparatus was used, employing a method ranging from 70°C to 400°C with a heating rate of 3°C per minute.

The equipment 1 used for obtaining purity values was an Agilent HPLC system composed of a G1365B Multi‐wavelength Detector (UV detector), G1329A Autosampler, G1312A Binary Pump, and G1379B Degasser. Data acquisition was performed using Agilent software. The analyses were carried out with a Phenomenex Luna C18(2) column (5 µm, 100 Å, 50 × 2 mm) at room temperature, with a flow rate of 0.3 mL/min and an injection volume of 5.0 μL. The mobile phase consisted of solvent A (0.1% formic acid in H₂O) and solvent B (CH₃CN), following the gradient program: 95% A and 5% B at 0.0 min, 0% A and 100% B at 12.0–13.4 min, returning to 95% A and 5% B at 14.5–17.0 min. Detection was performed at a UV wavelength of 254 nm. Mass spectra were obtained using an Agilent 6120 Single Quadrupole System, with a mass range of up to 2000 *m/z*. The chromatograms were manually integrated, and the quantification was carried out based on the area percentage.

The equipment 2 used for obtaining purity values was an Shimadzu HPLC system composed of a SPD‐M10A Multi‐wavelength Detector (UV detector), SIL‐10AF Autosampler, SCL‐10AV Controller, and LC‐6AD Binary Pump. Data acquisition was performed using Class VP software. The analyses were carried out with a Phenomenex Luna Phenyl‐hexyl column (5 µm, 100 Å, 250 × 4.6 mm) at room temperature, with a flow rate of 1.0 mL/min and an injection volume of 50.0 μL. The Method B used mobile phase consisted of solvent A (0.1% formic acid in H₂O) and solvent B (MeOH), following the gradient program: 50% A and 50% B at 0.0 min, 0% A and 100% B at 20.0 min, returning to 95% A and 5% B at 23.0–26.0 min. The Method C used mobile phase consisted of solvent A (0.1% formic acid in H₂O) and solvent B (MeOH), following the gradient program: 5% A and 95% B at 0.0 min, 0% A and 100% B at 33.0 min, returning to 95% A and 5% B at 33.0–40.0 min. Detection was performed at a UV wavelength of 254 nm.

#### 4.1.2 Procedures

**5‐methoxy‐2‐methyl‐pyridine (2)**. In a 50 mL flask equipped with a magnetic stir bar, 5‐hydroxy‐2‐methylpyridine (10 mmol, 1.09 g) was added. Subsequently, 15 mL of dimethyl sulfoxide and, finally, NaOH (40 mmol, 1.60 g) were added. The resulting mixture was kept under constant stirring at room temperature (32°C) for 2 h. After this stage, iodomethane (10 mmol, 0.62 mL) was added and kept under constant stirring at room temperature for an additional 2 h. Then, 10 mL of distilled water was added to the crude reaction material, which was subjected to extraction with ethyl ether (3 × 10 mL). The resulting organic phase was dried using magnesium sulfate and then concentrated under reduced pressure. The resulting crude compound was subjected to a purification process through normal‐phase column chromatography, using a mobile phase composed of hexanes and ethyl acetate in a ratio of 6:4. The purified product appeared as a slightly colorless oil, with a yield of 79%. Rf: 0.45 (7:3 hexanes:ethyl acetate). ^1^H NMR (400 MHz, CDCl_3_): *δ* 8.11 (s, 1H, 5‐C*H*_Pyridine_), 7.06–6.98 (m, 2H, 3‐C*H*_Pyridine_, 4‐C*H*_Pyridine_), 3.68 (s, 3H, 8‐OC*H*_3methoxy_), 2.36 (s, 3H, 7‐C*H*_3methyl_). ^13^C NMR (100 MHz, CDCl_3_): *δ* 153.7 (1 C, 5‐C_pyridine_), 150.2 (1 C, 2‐C_pyridine_), 135.9 (1 C, 6‐C_pyridine_), 123.4 (1 C, 4‐C_pyridine_), 121.5 (1 C, 3‐C_pyridine_), 55.6 (1 C, 8‐*C*_methoxy_), 23.2 (1 C, 7‐*C*_methyl_). GC‐MS (70 eV, *m/z*, relative abundance, %): 123 (100); 108 (60); 80(35); 53(38).

##### 4.1.2.1 General Procedure 1 (GP): Preparation of Benzonitriles

In a 50 mL flask, equipped with a magnetic stir bar, substituted benzaldehyde (1 equiv.) was added, followed by ammonium acetate (1.5 equiv.), sodium carbonate (1 equiv.), iodine (2.5 mol%), 8 mL of ethanol and, finally, tert‐butyl hydroperoxide (TBHP) (1.1 equiv.). The resulting mixture was maintained under constant stirring at a temperature of 50°C for 4 h. After this step, the crude reaction material was concentrated under reduced pressure, followed by the addition of 10 mL of distilled water and subsequent extraction with ethyl ether. The resulting organic phase was dried over magnesium sulfate, filtered, and then concentrated under reduced pressure. The resulting crude material was then subjected to a purification process through normal‐phase column chromatography, using a mobile phase composed of hexanes and ethyl acetate.

***p*** **‐chloro‐benzonitrile (3b)**: A white solid was obtained in 92% yield, using *p*‐chlorobenzaldehyde. ^1^H NMR (400 MHz, CDCl_3_): *δ* 7.60 (*d*, 2H, *J* = 8.4 Hz, 2‐CH_Phenyl_, 6‐CH_Phenyl_), 7.46 (*d*, 2H, *J* = 8.4 Hz, 3‐CH_Phenyl_, 5‐CH_Phenyl_). ^13^C NMR (100 MHz, CDCl_3_): *δ* 139.6 (1 C, 4‐C_Phenyl_), 133.4 (2 C, 2‐C_Phenyl_, 6‐C_Phenyl_), 129.7 (2 C, 3‐C_Phenyl_, 5‐C_Phenyl_), 118.0 (1 C, ‐*C*N), 110.8 (1 C, 1‐C_Phenyl_). GC‐MS (70 eV, *m/z*, relative abundance %): 137 (100); 139 (35); 102(34); 75(15). mp: 89.4°C–90.2°C.

***p*** **‐methyl‐benzonitrile (3c)**: A colorless oil was obtained in 64% yield, using *p*‐methylbenzaldehyde. ^1^H NMR (400 MHz, CDCl_3_): 7.47 (*d*, 2H, *J* = 8.0 Hz, 2‐CH_Phenyl_, 6‐CH_Phenyl_), 7.19 (*d*, 2H, *J* = 8.0 Hz, 3‐CH_Phenyl_, 5‐CH_Phenyl_), 2.35 (*s*, 3H, C*H*_3Methyl_). ^13^C NMR (100 MHz, CDCl_3_): *δ* 143.7 (1 C, 4‐C_Phenyl_), 132.1 (2 C, 2‐C_Phenyl_, 6‐C_Phenyl_), 129.8 (2 C, 3‐C_Phenyl_, 5‐C_Phenyl_), 119.2 (1 C, ‐*C*N), 109.3 (1 C, 1‐C_Phenyl_), 21.9 (1 C, O‐C_Methyl_). GC‐MS (70 eV, *m/z*, relative abundance %): 117 (100); 116 (60); 90 (49); 39 (24).

***p*** **‐methoxy‐benzonitrile (3d)**: A white solid was obtained in 72% yield, using *p*‐methoxybenzaldehyde. ^1^H NMR (400 MHz, CDCl_3_): *δ* 7.57 (*d*, 2H, *J* = 8.8 Hz, 2‐CH_Phenyl_, 6‐CH_Phenyl_), 6.94 (*d*, 2H, *J* = 8.8 Hz, 3‐CH_Phenyl_, 5‐CH_Phenyl_), 3.85 (*s*, 3H, C*H*_3Methoxy_). ^13^C NMR (100 MHz, CDCl_3_): *δ* 162.8 (1 C, 4‐C_Phenyl_), 134.0 (2 C, 2‐C_Phenyl_, 6‐C_Phenyl_), 119.3 (1 C, ‐*C*N), 114.8 (2 C, 3‐C_Phenyl_, 5‐C_Phenyl_), 103.9 (1 C, 1‐C_Phenyl_), 55.6 (1 C, O‐C_Methoxy_). GC‐MS (70 eV, *m/z*, relative abundance %): 133 (100); 103 (48); 90 (63); 63 (26). mp: 59.1°C–60.1°C.

**3,4‐dichloro‐benzonitrile (3e)**: A white solid was obtained in 97% yield, using 3,4‐dichlorobenzaldehyde. ^1^H NMR (400 MHz, CDCl_3_): *δ* 7.75 (*d*, 1H, *J* = 1.6 Hz, 2‐CH_Phenyl_), 7.57 (*d*, 1H, *J* = 8.4 Hz, 5‐C_Phenyl_), 7.50 (*dd*, 1H, *J* = 1.6, 8.4 Hz, 6‐C_Phenyl_). ^13^C NMR (100 MHz, CDCl_3_): *δ* 138.3 (1 C, 4‐C_Phenyl_), 134.0 (C, 3‐C_Phenyl_), 133.7 (1 C, 6‐C_Phenyl_), 131.5 (1 C, 2‐C_Phenyl_), 131.1 (1 C, 5‐C_Phenyl_), 116.8 (1 C, –*C*N), 112.0 (1 C, 1‐C_Phenyl_). GC‐MS (70 eV, *m/z*, relative abundance %): 172(66); 170(100); 136(23); 100(33); 50(28). mp: 72.0°C–72.8°C.

**3,4,5‐trimethoxybenzonitrile (3f)**: A pale yellow solid was obtained in 90% yield, using 3,4,5‐trimethoxybenzaldehyde. ^1^H NMR (400 MHz, CDCl_3_): *δ* 6.79 (*s*, 2H, 2‐CH_Phenyl_, 6‐CH_Phenyl_), 3.82 (*s*, 3H, 4‐OCH_3_), 3.80 (*s*, 6H, 3‐OCH_3_, 5‐OCH_3_). ^13^C NMR (100 MHz, CDCl_3_): *δ* 153.5 (2 C, 3‐C_Phenyl_, 5‐C_Phenyl_), 142.2 (1 C, 4‐C_Phenyl_), 119.0 (1 C, –*C*N), 109.4 (2 C, 2‐C_Phenyl_, 6‐C_Phenyl_), 106.7 (1 C, 1‐C_Phenyl_), 61.0 (1 C, 4‐OCH_3_), 56.4 (2 C, 3‐OCH_3_, 5‐OCH_3_). GC‐MS (70 eV, *m/z*, relative abundance %): 193(100); 178(71); 135(45); 118(33); 64(30). mp: 94.6°C–96.3°C.

##### 4.1.2.2 General Procedure 2 (GP2): Preparation of Ketone Intermediates

In a 20 mL reaction flask, duly purged with an inert atmosphere and kept moisture‐free, 2‐methylpyridine (1 equiv.) was added, which was dissolved in anhydrous THF (1 mmol/mL) at a specified temperature (−20°C for R^2^ = H, −78°C for R^2^ = OMe). Subsequently, a solution of lithium diisopropylamide (LDA) (1.05 equiv.), previously prepared, was added dropwise to the substrate, and the reaction mixture was maintained at the specified temperature (−20°C for R^2^ = H, −78°C for R^2^ = OMe) for a specified period (30 min. for R^2^ = H, 10 min for R^2^ = OMe). After this time interval, the desired substituted benzonitrile (1 equiv.) was added, remaining at the specified temperature (−20°C for R^2^ = H, −78°C for R^2^ = OMe) for 1 h. Subsequently, the mixture was heated to room temperature and maintained at this condition for 2 h. Finally, the crude reaction material was subjected to a 10% HCl solution, which remained for 15 min under agitation followed by neuralization with NaHCO_3_ and extraction with ethyl acetate. The resulting organic phase was dried over magnesium sulfate, filtered, and then concentrated under reduced pressure. The crude products were analyzed by gas chromatography–mass spectrometry (GC‐MS). All ketone intermediates bearing a 6‐methoxy substituent were used without further purification. Instead, they were passed through a short silica gel plug using a hexanes/ethyl acetate (6:4) mixture as the eluent. All ketone intermediates exhibited duplicated NMR signals due to keto–enol tautomerism, as previously reported in the literature [29].

**1‐phenyl‐2‐(pyridin‐2‐yl)ethanone (4a)** [29]: A yellow solid was obtained in 82% yield, using benzonitrile as electrophile. Rf: 0.25 (9:1 hexanes:ethyl acetate). ^1^H NMR (400 MHz, DMSO‐d_6_): Keto form *δ* 8.47 (*d*, 1H, *J* = 5.6 Hz, 6‐C_Pyridine_), 8.03 (*d*, 2H, *J* = 7.1 Hz 2‐CH_phenyl_, 6‐CH_phenyl_), 7.85 (*m*, 1H, 5‐CH_pyridine_), 7.77 (*m*, 2H, 3‐CH_phenyl_, 5‐CH_phenyl_), 7.64 (*m*, 1H, 4‐CH_phenyl_), 7.48–7.40 (*m*, 1H, 3‐CH_pyridine_), 7.24 (*d*, 1H, *J* = 8.2 Hz, 4‐CH_pyridine_), 4.54 (*s*, 2H, CH_2_). Enol form δ 8.41 (*d*, 1H, *J* = 5.1 Hz, 6‐C_Pyridine_), 7.85 (*m*, 2H, 2‐CH_phenyl_, 6‐CH_phenyl_), 7.77 (*m* 1H, 5‐CH_pyridine_), 7.48–7.40 (*m*, 2H, 3‐CH_phenyl_, 5‐CH_phenyl_), 7.38 (*m*, 1H, 3‐CH_pyridine_), 7.17(*m*, 1H, 4‐CH_pyridine_), 6.38 (*s*, 1H, CH_enol_). ^13^C NMR (100 MHz, DMSO‐d_6_): Keto form *δ* 197.0 (C═O), 124.4(4‐C_Phenyl_), 155.7 (2‐C_pyridine_), 149.1 (6‐C_pyridine_), 137.9 (5‐C_pyridine_), 135.9 (1‐C_phenyl_), 129.5 (2‐C_phenyl_, 6‐C_phenyl_), 128.4 (3‐C_phenyl_, 5‐C_phenyl_), 125.1 (3‐C_pyridine_), 124.4(4‐C_Phenyl_) 121.9 (4‐C_pyridine_), 47.6 (CH_2_). Enol form δ 157.7 (2‐C_pyridine_), 144.2 (6‐C_pyridine_), 137.9 (5‐C_pyridine_), 136.4 (1‐C_phenyl_), 133.9 (═C–OH), 128.7 (2‐C_phenyl_, 6‐C_phenyl_), 126.9 (3‐C_phenyl_, 5‐C_phenyl_), 125.1 (4‐C_phenyl_) 122.8 (3‐C_pyridine_), 119.0 (4‐C_pyridine_), 93.7 (C═C_enol_). GC‐MS (70 eV, *m/z*, relative abundance %): 196(40); 195(100); 92(15); 65(17); 51(14). mp: 50.8 – 52.1°C. Purity (Equipment 2, Method B): 99.0% (15.3 min).

**1‐(4‐chlorophenyl)‐2‐(pyridin‐2‐yl)ethanone (4b)** [29]: A yellow solid was obtained in 87% yield, using 4‐chlorobenzonitrile as electrophile. Rf: 0.30 (9:1 hexanes:ethyl acetate). ^1^H NMR (400 MHz, DMSO‐d_6_): Keto form *δ* 8.57 (*d*, 1H, *J* = 5.0 Hz, 6‐C_Pyridine_), 8.01 (*d*, 2H, *J* = 8.6 Hz 2‐CH_phenyl_, 6‐CH_phenyl_), 7.77 (*m*, 1H, 5‐CH_pyridine_), 7.43 (*d*, 2H, *J* = 8.6 Hz, 3‐CH_phenyl_, 5‐CH_phenyl_), 7.40–7.34 (*m*, 1H, 3‐CH_pyridine_), 7.06 (*d*, 1H, *J* = 8.2 Hz, 4‐CH_pyridine_), 4.56 (*s*, 2H, CH_2_). Enol form *δ* 8.27 (*d*, 1H, *J* = 5.0 Hz, 6‐C_Pyridine_), 7.77 (*m*, 2H, 2‐CH_phenyl_, 6‐CH_phenyl_), 7.61 (*t*, 1H, *J* = 8.6 Hz, 5‐CH_pyridine_), 7.40–7.34 (*m*, 2H, 3‐CH_phenyl_, 5‐CH_phenyl_), 7.28 (*d*, 1H, *J* = 7.3 Hz, 3‐CH_pyridine_), 7.02–6.96 (*m*, 1H, 4‐CH_pyridine_), 6.04 (*s*, 1H, CH_enol_). ^13^C NMR (100 MHz, DMSO‐d_6_): Keto form *δ* 195.3 (C═O), 163.7 (4‐C_Phenyl_), 154.4 (2‐C_pyridine_), 148.5 (6‐C_pyridine_), 137.9 (5‐C_pyridine_), 135.2 (1‐C_phenyl_), 130.4 (2‐C_phenyl_, 6‐C_phenyl_), 128.6 (3‐C_phenyl_, 5‐C_phenyl_), 125.0 (3‐C_pyridine_), 121.8 (4‐C_pyridine_), 47.6 (CH_2_). Enol form *δ* 158.3 (2‐C_pyridine_), 144.1 (6‐C_pyridine_), 140.1 (4‐C_phenyl_), 137.4 (5‐C_pyridine_), 135.2 (1‐C_phenyl_), 134.7 (═C–OH), 129.1 (2‐C_phenyl_, 6‐C_phenyl_), 126.9 (3‐C_phenyl_, 5‐C_phenyl_), 122.6 (3‐C_pyridine_), 118.7 (4‐C_pyridine_), 94.2 (C═C_enol_). GC‐MS (70 eV, *m/z*, relative abundance %): 230(28); 202(35); 139(100); 111(68); 75(47). mp: 83.8 – 84.8°C. HRAM‐MS (ESI+) calc for C_13_H_10_ClNO_2_ [M + H], 232.0534; found, 232.0521. Purity (Equipment 2, Method C): 99.2% (8.9 min).

**1‐(4‐methoxyphenyl)‐2‐(pyridin‐2‐yl)ethanone (4c)** [29]: A yellow solid was obtained in 74% yield, using 4‐methoxybenzonitrile as electrophile. Rf: 0.23 (9:1 hexanes:ethyl acetate). ^1^H NMR (400 MHz, DMSO‐d_6_): Keto form *δ* 8.46 (d, *J* = 4.9 Hz, 1H, 6‐CH_pyridine_), 8.01 (d, *J* = 9.0 Hz, 2H, 2‐CH_phenyl_, 6‐CH_phenyl_), 7.76–7.72 (m, 1H, 5‐CH_pyridine_), 7.35 (d, *J* = 7.8 Hz, 1H, 3‐CH_pyridine_), 7.25 (m, 1H, 4‐CH_pyridine_), 7.04 (d, *J* = 9.0 Hz, 2H, 3‐CH_phenyl_, 5‐CH_phenyl_), 4.46 (s, 2H, CH_2_), 3.83 (s, 3H, –OCH_3_), 3.80 (s, 1H). – Enol form *δ* 8.34 (d, 6‐CH_pyridine_), 7.79 (d, 2‐CH_phenyl_, 6‐CH_phenyl_), 7.22 (m, 3‐CH_pyridine_), 7.05 (m, 4‐CH_pyridine_), 6.98 (d, 3‐CH_phenyl_, 5‐CH_phenyl_) 6.25 (s, CH_enol_), 3.80 (s, –OCH_3_) ^13^C NMR (101 MHz, DMSO‐d_6_) – Keto Form *δ* 195.4 (C═O), 163.6 (4‐C_Phenyl_), 156.0 (2‐C_pyridine_), 149.0 (6‐C_pyridine_), 136.5 (5‐C_pyridine_), 130.8 (2‐C_phenyl_, 6‐C_phenyl_), 129.3 (1‐C_Phenyl_), 124.3 (3‐C_pyridine_), 121.7 (4‐C_pyridine_), 113.9 (3‐C_phenyl_, 5‐C_phenyl_), 47.4 (CH_2_), 55.5(‐OC_methoxy_), 47.4 (–OCH_3_). – Keto Form *δ* 163.2 (4‐C_Phenyl_), 160.4 (=C–OH), 157.9 (2‐C_pyridine_), 143.9 (6‐C_pyridine_), 137.8 (5‐C_pyridine_), 128.4 (1‐C_Phenyl_), 126.7 (2‐C_phenyl_, 6‐C_phenyl_), 121.5 (3‐C_pyridine_), 118.4 (4‐C_pyridine_), 113.8 (3‐C_phenyl_, 5‐C_phenyl_), 92.2 (C═C_enol_), 55.2(–OC_methoxy_). GC‐MS (70 eV, *m/z*, relative abundance %): 227(7); 199(21); 135(100); 92(28); 77(30). mp: 73.8°C–75.3°C. HRAM‐MS (ESI+) calc for C_14_H_14_NO_2_ [M + H], 228.1019; found, 228.1011. Purity (Equipment 2, Method B): 99.2% (8.9 min).

**2‐(pyridin‐2‐yl)‐1‐(p‐tolyl)ethanone (4d)** [29]: A yellow solid was obtained in 68% yield, using 4‐methylbenzonitrile as electrophile. Rf: 0.13 (9:1 hexanes:ethyl acetate). ^1^H NMR (400 MHz, DMSO‐d_6_): Keto form *δ* 8.47 (*d*, 1H, *J* = 4.8 Hz, 6‐CH_pyridine_), 7.93 (*d*, 2H, *J* = 8.1 Hz, 2‐CH_phenyl_, 6‐CH_phenyl_), 7.76 (*m*, 1H, 4‐CH_pyridine_), 7.58–7.53 (*m*, 1H, 3‐CH_pyridine_), 7.34 (*m*, 3H, 3‐CH_phenyl_, 5‐CH_phenyl_, 3‐CH_pyridine_), 7.15–7.09 (*m*, 1H, 5‐CH_pyridine_), 4.49 (*s*, 2H, CH_2Keto_), 2.36 (*s*, 3H, ‐CH_3_). Enol form *δ* 8.39 (*d*, 1H, *J* = 5.1 Hz, 6‐CH_pyridine_), 7.76 (*m*, 3H, 2‐CH_phenyl_, 6‐CH_phenyl_, 4‐CH_pyridine_), 7.25 (*m*, 4H, 3‐CH_phenyl_, 5‐CH_phenyl_, 5‐CH_pyridine_), 6.32 (*s*, 1H, ‐CH_enol_), 2.34 (*s*, 3H, –CH_3_). ^13^C NMR (100 MHz, DMSO‐d_6_): Keto form *δ* 196.6 (C═O), 163.4 (4‐C_Phenyl_), 157.9 (1‐C_pyridine_), 149.1 (6‐C_pyridine_), 136.6 (5‐C_pyridine_), 129.3 (1‐C_phenyl_), 129.1 (2‐C_phenyl_, 6‐C_phenyl_), 128.6 (3‐C_phenyl_, 5‐C_phenyl_), 124.5 (3‐C_pyridine_), 47.6 (C═C_enol_), 21.2 (–CH_3_). Enol form 163.4 (4‐C_Phenyl_), 144.3 (═C–OH), 143.8 (6‐C_pyridine_), 139.2 (5‐C_pyridine_), 138.0 (1‐C_phenyl_), 121.8 (4‐C_pyridine_), 121.8, 118.9, 93.2 (C + =C_enol_), 155.9 (2‐C_pyridine_), 129.0 (2‐C_phenyl_, 6‐C_phenyl_), 125.8 (3‐C_phenyl_, 5‐C_phenyl_), 125.8 (4‐C_phenyl_), 125.1 (3‐C_pyridine_), 21.0 (–CH_3_). GC‐MS (70 eV, *m/z*, relative abundance %): 210(45); 209(100); 92(12); 65(18). mp: 83.8°C–84.8°C. HRAM‐MS (ESI+) calc for C_14_H_14_NO [M + H], 212.1069; found, 212.1067. Purity (Equipment 2, Method C): 97.8% (18.2 min).

**1‐(3,4‐dichlorophenyl)‐2‐(pyridin‐2‐yl)ethanone (4e)**: A yellow solid was obtained in 92% yield, using 3,4‐dichlorobenzonitrile as electrophile. Rf: 0.41 (9:1 hexanes:ethyl acetate). ^1^H NMR (400 MHz, DMSO‐d_6_) enol‐form: δ 8.39 (*d*, 1H, *J* = 5.1 Hz, 6‐CH_pyridine_), 8.03 (*d*, 1H, *J* = 2.0 Hz), 7.81 (*dd*, 2H, *J* = 8.5, 2.1 Hz, 6‐CH_pyridine_), 7.69 (*d*, 1H, *J* = 8.5 Hz, 6‐CH_phenyl_), 7.28 (*d*, 1H, *J* = 8.2 Hz, 6‐CH_phenyl_), 7.16 (*ddd*, 1H, *J* = 7.0, 5.4, 1.2 Hz, 3‐CH_pyridine_), 6.47 (*s*, 1H, CH_enol_) 4.56 (*s*, 0.4 H, CH_2Keto_). ^13^C NMR (100 MHz, DMSO‐d_6_): *δ* 162.0 (C═O), 157.0 (1‐C_pyridine_), 143.5 (6‐C_pyridine_), 138.4 (4‐C_pyridine_), 137.1 (1‐C_phenyl_), 131.8 (5‐C_phenyl_, 4‐C_phenyl_), 131.5 (2‐C_phenyl_, 3‐C_phenyl_), 130.7 (6‐C_phenyl_), 126.9 (6‐C_phenyl_), 125.4 (3‐C_pyridine_), 122.2 (5‐C_pyridine_), 119.1, 94.2 (C_enol_). GC‐MS (70 eV, *m/z*, relative abundance %): 264(28); 268(13); 236(52); 173(100); 145(74). mp: 104.1°C–105.6°C. HRAM‐MS (ESI+) calc for C_13_H_10_Cl_2_NO [M + H], 266.0133; found, 266.0131. Purity (Equipment 2, Method B): 98.5% (15.7 min).

**2‐(pyridin‐2‐yl)‐1‐(3,4,5‐trimethoxyphenyl)ethenone (4f):** A pale yale solid was obtained in 72% yield, using 3,4,5‐trimethoxybenzonitrile as electrophile. Rf: 0.46 (8:2 hexanes:ethyl acetate). ^1^H NMR (500 MHz, CDCl_3_): *δ* 8.56 (d, *J *= 4.5 Hz, 1H, 6‐CH_pyridine_), 7.70 (t, *J* = 7.6 Hz, 1H, 5‐CH_pyridine_), 7.37 (s, 1H, 3‐CH_pyridine_), 7.36 (s, 2H, 2‐CH_trimethoxybenzene_, 6‐CH_trimethoxybenzene_), 7.24–7.21 (m, 1H, 4‐CH_pyridine_), 4.52 (s, 2H, –CH_2_), 3.89 (s, 12H, 3‐OCH_3trimethoxybenzene_, 5‐OCH_3trimethoxybenzene_, 4‐OCH_3trimethoxybenzene_). ^13^C NMR (125 MHz, CDCl_3_): δ 195.4 (C═O), 155.2 (4‐C_trimethoxybenzene_), 153.1 (3‐C_trimethoxybenzene_, 5‐C_trimethoxybenzene_), 148.8 (1‐C_trimethoxybenzene_), 137.4 (6‐C_pyridine_), 131.6 (2‐C_pyridine_), 124.5 (4‐C_pyridine_), 122.3 (3‐C_pyridine_) 118.3 (5‐C_pyridine_), 106.7 (2‐C_trimethoxybenzene_, 6‐C_trimethoxybenzene_), 60.9 (4‐OCH_3trimethoxybenzene_), 56.4 (3‐OCH_3trimethoxybenzene_, 5‐OCH_3trimethoxybenzene_), 48.2 (–CH_2_). GC‐MS (70 eV, *m/z*, relative abundance %): 287 (19); 259 (21); 195 (100); 152 (11). HRAM‐MS (ESI+) calc for C_16_H_18_NO_4_ [M + H], 288.1236; found, 288.1240. Purity (Equipment 2, Method B): 95.7% (15.6 min).

**2‐(5‐methoxypyridin‐2‐yl)‐1‐(3,4,5‐trimethoxyphenyl)ethenone (5f):** A yellow solid was obtained in 65% yield, using 3,4,5‐trimethoxybenzonitrile as electrophile. Rf: 0.16 (9:1 hexanes:ethyl acetate). ^1^H NMR (300 MHz, CDCl_3_): *δ* 8.24 (*d*, 1H, *J* = 3 Hz, 6‐CH_pyridine_), 7.36 (*s*, 2H, 2‐CH_trimethoxybenzene_, 6‐CH_trimethoxybenzene_), 7.27 (*d*, 1H, *J* = 4.5 Hz, 3‐CH_pyridine_), 7.20 (*dd*, 1H, *J* = 2.7, 8.4 Hz, 4‐CH_pyridine_), 4.44 (*s*, 2H, –CH_2_), 3.89 (*s*, 9H, 3‐OCH_3trimethoxybenzene_, 5‐OCH_3trimethoxybenzene_, 5‐OCH_3pyridine_), 3.83 (*s*, 3H, 4‐OCH_3trimethoxybenzene_). ^13^C NMR (75 MHz, CDCl_3_): δ 195.9 (C═O), 154.8 (4‐C_trimethoxybenzene_), 153.1 (3‐C_trimethoxybenzene_, 5‐C_trimethoxybenzene_), 147.0 (1‐C_trimethoxybenzene_), 142.7 (6‐C_pyridine_), 136.2 (5‐C_pyridine_), 131.6 (2‐C_pyridine_), 124.6 (4‐C_pyridine_), 122.2 (3‐C_pyridine_), 106.5 (2‐C_trimethoxybenzene_, 6‐C_trimethoxybenzene_), 61.0 (5‐OCH_3pyridine_), 56.3 (3‐OCH_3trimethoxybenzene_, 5‐OCH_3trimethoxybenzene_), 55.8 (4‐OCH_3trimethoxybenzene_), 47.3 (–CH_2_). GC‐MS (70 eV, *m/z*, relative abundance %): 317 (10); 289 (17); 195 (100); 195 (10). mp: 72.1°C–75.3°C. HRAM‐MS (ESI+) calc for C_17_H_20_NO_5_ [M + H], 318.1341; found, 318.1334. Purity (Equipment 1, Method A): 97.6% (6.5 min).

##### 4.1.2.3 General Procedure 3 (GP3): Preparation of Indolizines

In a 20 mL flask, the keto intermediate (1.0 equiv.) and sodium bicarbonate (4.0 equiv.) were combined and dissolved in acetone. In a subsequent step, an α‐haloketone or α‐chloroaldehyde (2.0 equiv.) was added to the reaction mixture, which was refluxed overnight. After this time, the crude reaction material was extracted with water and ethyl acetate. The resulting organic phase was dried using magnesium sulfate and subsequently concentrated under reduced pressure. Finally, the crude reaction compound was analyzed by gas chromatography coupled with mass spectrometry (GC‐MS) and then subjected to a purification process through column chromatography using a mixture of hexanes and ethyl acetate as eluent. The purity of all compounds was evaluated using HPLC‐UV‐MS, following the chromatography method described in the general procedure.

**(Indolizin‐1‐yl)(phenyl)methanone** (**6a**): A yellow solid was obtained in 82% yield, using α‐chloroaldehyde and **4a**. Rf: 0.47 (8:2 hexanes:ethyl acetate). ^1^H NMR (400 MHz, CDCl_3_): *δ* 8.45 (*d*, 1H, *J* = 9.2 Hz, 5‐C*H*_Indolizine_), 8.02 (*d*, 1H, *J* = 6.4 Hz, 2‐C*H*_indolizine_), 7.82 (*d*, 2H, *J* = 6.4 Hz, 2‐C*H*_phenyl_, 6‐C*H*_phenyl_), 7.47 (m, 3H, 1‐C*H*_phenyl_, 3‐C*H*_phenyl_, 5‐C*H*_phenyl_), 7.21 (*d*, 1H, 2.8 Hz, 6‐C*H*_Indolizine_), 7.14 (*t*, 1H, *J* = 6.8 Hz, 7‐C*H*_Indolizine_), 7.04 (*d*, 1H, *J* = 2.4 Hz, 8‐C*H*_Indolizine_), 6.79 (t, 1H, *J* = 6.4 Hz, 3‐C*H*_indolizine_). ^13^C NMR (100 MHz, CDCl_3_): *δ* 190.4 (C═O), 141.0 (1‐C_Indolizine_), 136.9 (1‐C_phenyl_), 130.8 (4‐C_phenyl_), 128.9 (2‐C_phenyl_, 6‐C_phenyl_), 128.1 (3‐C_phenyl_, 5‐C_phenyl_), 126.0 (2‐C_Indolizine_), 124.2 (6‐C_Indolizine_), 120.8 (5‐C_Indolizine_), 118.5 (7‐C_Indolizine_), 113.9 (8‐C_Indolizine_), 113.8 (3‐C_Indolizine_), 112.3 (9‐C_Indolizine_). GC‐MS (70 eV, *m/z*, relative abundance %): 221(51); 144(100); 116(15); 89(51); 77(14). mp: 98.5°C–100.9°C. HRAM‐MS (ESI+) calc for C_15_H_12_NO [M + H], 222.0913; found, 222.0907. Purity (Equipment 2, Method B): 98.9% (14.2 min).

**(4‐chlorophenyl)(indolizin‐1‐yl)methanone** (**6b**): A yellow solid was obtained in 87% yield, using α‐chloroaldehyde and **4b**. Rf: 0.22 (8:2 hexanes:ethyl acetate). ^1^H NMR (400 MHz, CDCl_3_): δ 8.46 (d, *J* = 9.0 Hz, 1H, 5‐C*H*_Indolizine_), 8.07 (d, *J* = 6.8 Hz, 1H, 2‐C*H*_indolizine_), 7.77 (d, *J* = 8.6 Hz, 2H, 2‐C*H*_phenyl_, 6‐C*H*_phenyl_), 7.44 (d, *J* = 8.6 Hz, 2H, 3‐C*H*_phenyl_, 5‐C*H*_phenyl_), 7.25 (d, *J* = 3.0 Hz, 1H, 6‐C*H*_indolizine_), 7.20 (ddd, *J* = 9.0, 6.8, 1.1 Hz, 1H, 7‐C*H*_Indolizine_), 7.03 (d, *J* = 3.0 Hz, 1H, 8‐C*H*_Indolizine_), 6.84 (td, *J* = 6.8, 1.2 Hz, 1H, 3‐C*H*_indolizine_). ^13^C NMR (100 MHz, CDCl_3_): δ 189.0 (C═O), 139.6 (1‐C_Indolizine_), 137.2 (1‐C_phenyl_), 137.1 (4‐C_phenyl_), 130.5 (2‐C_phenyl_, 6‐C_phenyl_), 128.5 (3‐C_phenyl_, 5‐C_phenyl_), 126.2 (2‐C_Indolizine_), 124.5 (6‐C_Indolizine_), 121.0 (5‐C_Indolizine_), 118.4 (7‐C_Indolizine_), 114.2 (8‐C_Indolizine_), 114.1 (3‐C_Indolizine_), 112.3 (9‐C_Indolizine_). GC‐MS (70 eV, *m/z*, relative abundance %): 257(12); 255(38); 144(100); 116(17); 89(56); 75(14). mp: 115.7°C–117.3°C. HRAM‐MS (ESI+) calc for C_15_H_11_NOCl [M + H], 256.0523; found, 256.0523. Purity (Equipment 1, Method A): 98.1% (9.4 min).

**(Indolizin‐1‐yl)(p‐tolyl)methanone** (**6c**): A white solid was obtained in 74% yield, using α‐chloroaldehyde and **4c**. Rf: 0.47 (8:2 hexanes:ethyl acetate). ^1^H NMR (400 MHz, CDCl_3_): *δ* 8.46 (*d*, 1H, *J* = 9.0 Hz, 5‐C*H*_Indolizine_), 8.05 (*dt*, 1H *J* = 6.8, 1.1 Hz, 2‐C*H*_indolizine_), 7.75 (*d*, 2H, *J* = 8.1 Hz, 2‐C*H*_phenyl_, 6‐C*H*_phenyl_), 7.27 (*d*, 2H, *J* = 7.8 Hz, 3‐C*H*_phenyl_, 5‐C*H*_phenyl_), 7.23 (*d*, 1H, *J* = 3.0 Hz, 6‐C*H*_Indolizine_), 7.16 (*ddd*, 1H, *J* = 9.0, 6.7, 1.1 Hz, 7‐C*H*_Indolizine_), 7.09 (*d*, 1H, *J* = 3.0 Hz, 8‐C*H*_Indolizine_), 6.80 (*td*, 1H, *J* = 6.8, 1.3 Hz, 3‐C*H*_indolizine_), 2.43 (*s*, 3H, ‐CH_3Methyl_). ^13^C NMR (100 MHz, CDCl_3_): *δ* 190.2 (C═O), 141.3 (1‐C_Indolizine_), 138.4 (1‐C_phenyl_), 137.0 (4‐C_phenyl_), 129.2 (2‐C_phenyl_, 6‐C_phenyl_), 128.9 (3‐C_phenyl_, 5‐C_phenyl_), 126.1 (2‐C_Indolizine_), 124.0 (6‐C_Indolizine_), 121.1 (5‐C_Indolizine_), 118.6 (7‐C_Indolizine_), 113.9 (8‐C_Indolizine_), 113.8 (3‐C_Indolizine_), 112.6 (9‐C_Indolizine_), 21.6 (‐C_Methyl_). GC‐MS (70 eV, *m/z*, relative abundance %): 235(72); 206(12); 144(100); 89(25). mp: 121.8°C–123.2°C. HRAM‐MS (ESI+) calc for C_16_H_14_NO [M + H], 236.1080; found, 236.1067. Purity (Equipment 1, Method A): 95.7% (9.0 min).

**(Indolizin‐1‐yl)(4‐methoxyphenyl)methanone** (**6d**): A white solid was obtained in 68% yield, using α‐chloroaldehyde and **4d**. Rf: 0.38 (8:2 hexanes:ethyl acetate). ^1^H NMR (400 MHz, CDCl_3_): *δ* 8.43 (d, *J* = 9.0 Hz, 1H, 5‐C*H*_Indolizine_), 8.05 (d, *J* = 6.8 Hz, 1H, 2‐C*H*_indolizine_), 7.85 (d, *J* = 8.9 Hz, 2H, 2‐C*H*_phenyl_, 6‐C*H*_phenyl_), 7.25 (d, *J *= 3.0 Hz, 1H, 6‐C*H*_Indolizine_), 7.17–7.12 (m, 1H. 7‐C*H*_Indolizine_), 7.10 (d, *J* = 2.9 Hz, 1H, 8‐C*H*_Indolizine_), 6.98 (d, *J* = 8.8 Hz, 2H, 3‐C*H*_phenyl_, 5‐C*H*_phenyl_), 6.80 (td, *J *= 6.8, 1.2 Hz, 1H, 3‐C*H*_indolizine_), 3.88 (s, 3H, –OCH_3_). ^13^C NMR (100 MHz, CDCl_3_): *δ* 189.3 (C═O), 162.2 (4‐C_phenyl_), 137.0 (1‐C_Indolizine_), 133.6 (1‐C_phenyl_), 131.3 (3‐C_phenyl_, 5‐C_phenyl_), 126.1 (2‐C_Indolizine_), 123.9 (6‐C_Indolizine_), 121.1 (5‐C_Indolizine_), 118.4 (7‐C_Indolizine_), 113.9 (8‐C_Indolizine_), 113.7 (3‐C_Indolizine_), 113.5 (2‐C_phenyl_, 6‐C_phenyl_), 112.7 (9‐C_Indolizine_), 55.5 (–OC_Methoxy_). GC‐MS (70 eV, *m/z*, relative abundance %): 251(70); 208(12); 144(100); 89(37). mp: 121.8°C–123.2°C. HRAM‐MS (ESI+) calc for C_16_H_13_NaNO_2_ [M + Na], 274.0844; found, 274.0836. Purity (Equipment 1, Method A): 98.8% (8.5 min).

**(3,4‐dichlorophenyl)(indolizin‐1‐yl)methanone** (**6e**): A white solid was obtained in 92% yield, using α‐chloroaldehyde and **4e**. Rf: 0.39 (8:2 hexanes:ethyl acetate). ^1^H NMR (400 MHz, CDCl_3_): *δ* 8.40 (*d*, 1H, *J* = 9.0 Hz, 5‐C*H*_Indolizine_), 8.02 (*d*, 1H, *J* = 6.8 Hz, 2‐C*H*_indolizine_), 7.84 (*d*, 1H, *J* = 1.9 Hz, 2‐CH_phenyl_), 7.58 (*dd*, 1H, *J* = 8.2, 2.0 Hz, 6‐CH_phenyl_), 7.48 (*d*, 1H, *J* = 8.2 Hz, 5‐CH_phenyl_), 7.19 (*d*, 1H, *J* = 3.1 Hz, 6‐C*H*_Indolizine_), 7.18–7.14 (*m*, 1H, 7‐C*H*_Indolizine_), 6.95 (*d*, 1H, *J* = 3.1 Hz, 8‐C*H*_Indolizine_), 6.80 (*td*, 1H, *J* = 6.8, 1.2 Hz, 3‐C*H*_indolizine_). ^13^C NMR (100 MHz, CDCl_3_): *δ* 187.2 (C═O), 140.8 (1‐C_Indolizine_), 137.2 (1‐C_phenyl_), 134.9 (4‐C_phenyl_), 132.6 (3‐C_phenyl_), 130.8 (2‐C_phenyl_), 130.2 (6‐C_phenyl_), 128.0 (6‐C_phenyl_), 126.1 (2‐C_Indolizine_), 124.7 (6‐C_Indolizine_), 120.9 (5‐C_Indolizine_), 118.1 (7‐C_Indolizine_), 114.2 (8‐C_Indolizine_), 114.1 (3‐C_Indolizine_), 111.7 (9‐C_Indolizine_). GC‐MS (70 eV, *m/z*, relative abundance %): 289(33); 291(22); 144(100); 89(28). mp: 158.3°C–160.4°C. HRAM‐MS (ESI+) calc for C_15_H_10_NOCl_2_ [M + H], 290.0133; found, 290.0128. Purity (Equipment 1, Method A): 98.6% (10.1 min).

**(Indolizin‐1‐yl)(3,4,5‐Trimethoxyphenyl)Methanone (6f):** A white solid was obtained in 71% yield, using α‐chloroaldehyde and **4f**. Rf: 0.22 (8:2 hexanes:ethyl acetate). ^1^H NMR (400 MHz, CDCl_3_): *δ* 8.59 (*d*, 1H, *J* = 9.0 Hz, 5‐C*H*_Indolizine_), 8.22 (*d*, 1H, *J* = 6.8 Hz, 2‐C*H*_indolizine_), 7.42 (*t*, 1H, *J* = 3.8 Hz, 6‐C*H*_Indolizine_), 7.36–7.28 (*m*, 2H, 7‐C*H*_Indolizine_, 8‐C*H*_Indolizine_), 7.26 (s, 2H, 2‐C*H*_phenyl_, 6‐C*H*_phenyl_), 6.97 (*t*, 1H, *J* = 6.7 Hz, 3‐C*H*_Indolizine_), 4.07 (*s*, 3H), 4.05 (*s*, 6H). ^13^C NMR (100 MHz, CDCl_3_): *δ* 189.4 (C═O), 152.8 (3‐C_Phenyl_, 5‐C_Phenyl_), 140.4 (4‐C_phenyl_), 136.4 (1‐C_Indolizine_), 126.1 (2‐C_Indolizine_), 124.2 (6‐C_Indolizine_), 120.8 (5‐C_Indolizine_), 118.2 (7‐C_Indolizine_), 113.9 (8‐C_Indolizine_), 113.8 (3‐C_Indolizine_), 112.1 (1‐C_phenyl_), 106.4 (2‐C_phenyl_, 6‐C_phenyl_), 60.9 (4‐OC_Methoxy_), 56.2 (3‐OC_Methoxy_, 5‐OC_Methoxy_). GC‐MS (70 eV, *m/z*, relative abundance %): 311(77); 268(15); 144(100); 89(27). mp: 164.3°C–165.4°C. HRAM‐MS (ESI+) calc for C_18_H_19_NO_4_ [M + H], 312.1230; found, 312.1238. Purity (Equipment 1, Method A): 96.6% (8.2 min).

**(6‐methoxyindolizin‐1‐yl)(phenyl)methanone** (**7a**): A pale green solid was obtained in 75% yield, using α‐chloroaldehyde and **5a**. Rf: 0.54 (7:3 hexanes:ethyl acetate). ^1^H NMR (400 MHz, CDCl_3_): *δ* 8.38 (*d*, 1H, *J* = 9.7 Hz, 8‐CH_indolizine_), 7.81 (*dd*, 2H, *J* = 8.1, 1.5 Hz, 2‐CH_Phenyl_, 6‐CH_Phenyl_), 7.61 (*d*, 1H, *J* = 2.0 Hz, 5‐CH_indolizine_), 7.53–7.43 (*m*, 3H, 3‐CH_Phenyl_, 4‐CH_Phenyl_, 5‐CH_Phenyl_), 7.17 (*d*, 1H, *J* = 2.9 Hz, 3‐CH_indolizine_), 6.99 (*d*, 1H, *J* = 9.6, 7‐CH_indolizine_), 6.96 (*d*, 1H, *J* = 2.2 Hz, 2‐CH_indolizine_), 3.82 (*s*, 3H, –OC*H*_3_).^13^C NMR (100 MHz, CDCl_3_): *δ* 190.2 (C═O), 150.3 (6‐C_indolizine_), 141.0 (1‐C_phenyl_), 133.7 (8‐C_indolizine_), 130.9 (4‐C_phenyl_), 129.0 (2‐C_phenyl_, 6‐C_phenyl_), 128.2 (3‐C_phenyl_, 5‐C_phenyl_), 121.2 (8‐C_indolizine_), 119.0 (3‐C_indolizine_), 118.4 (2‐C_indolizine_), 114.7 (7‐C_indolizine_), 112.4 (1‐C_indolizine_), 108.3 (5‐C_indolizine_). GC‐MS (70 eV, *m/z*, relative abundance %): 251(70); 174(100); 111(10); 77(12). mp: 158.2°C–159.2°C. HRAM‐MS (ESI+) calc for C_14_H_16_NO_2_ [M + H], 252.1019; found, 252.1025. Purity (Equipment 1, Method A): 99.6% (8.8 min).

**(4‐chlorophenyl)(6‐methoxyindolizin‐1‐yl)methanone** (**7b**): A yellow solid was obtained in 40% yield, using α‐chloroaldehyde and **5b**. Rf: 0.50 (7:3 hexanes:ethyl acetate). ^1^H NMR (400 MHz, CDCl_3_): *δ* 8.35 (*d*, 1H, *J* = 9.7 Hz, 8‐CH_indolizine_), 7.75 (*d*, 2H, *J* = 8.5 Hz, 2‐CH_Phenyl_, 6‐CH_Phenyl_), 7.59 (*s*, 1H, 5‐CH_indolizine_), 7.42 (*d*, 2H, *J* = 8.5 Hz, 3‐CH_Phenyl_, 5‐CH_Phenyl_), 7.16 (*d*, 1H, *J* = 2.9 Hz, 3‐CH_indolizine_), 6.98 (*d*, 1H, *J* = 9.7 Hz, 7‐CH_indolizine_), 6.93 (*d*, 1H, *J* = 2.9 Hz, 2‐CH_indolizine_), 3.81 (*s*, 3H, –OC*H*_3_).^13^C NMR (100 MHz, CDCl_3_): *δ* 188.6 (C═O), 150.3 (6‐C_indolizine_), 139.4 (4‐C_phenyl_), 136.8 (1‐C_phenyl_), 133.7 (8‐C_indolizine_), 130.3 (2‐C_phenyl_, 6‐C_phenyl_), 128.4 (3‐C_phenyl_, 5‐C_phenyl_), 121.0 (8‐C_indolizine_), 119.2 (3‐C_indolizine_), 118.0 (2‐C_indolizine_), 114.7 (7‐C_indolizine_), 112.0 (1‐C_indolizine_), 108.2 (5‐C_indolizine_), 55.9 (–O*C*H_3_). GC‐MS (70 eV, *m/z*, relative abundance %): 285(60); 187(20); 174(100); 119(10); 76(09). mp: 140.3°C–142.0°C. HRAM‐MS (ESI+) calc for C_17_H_13_ClNO_2_ [M + H], 286.0629; found, 286.0628. Purity (Equipment 1, Method A): 96.8% (7.7 min).

**(6‐methoxyindolizin‐1‐yl)(p‐tolyl)methanone** (**7c**): A yellow solid was obtained in 85% yield, using α‐chloroaldehyde and **5c**. Rf: 0.52 (7:3 hexanes:ethyl acetate). ^1^H NMR (400 MHz, CDCl_3_): *δ* 8.36 (*d*, 1H, *J* = 9.7 Hz, 8‐CH_Indolizine_), 7.74 (*d*, 2H, *J* = 8.0 Hz, 2‐CH_Phenyl_, 6‐CH_Phenyl_), 7.59 (*s*, 1H, 5‐CH_Indolizine_), 7.26 (*d*, 2H, *J* = 7.9 Hz, 3‐CH_Phenyl_, 5‐CH_Phenyl_), 7.17 (*d*, 1H, *J* = 2.8 Hz, 3‐CH_Indolizine_), 7.01 (*d*, 1H, *J* = 2.7 Hz, 2‐CH_Indolizine_), 6.96 (*dd*, 1H, *J* = 9.7, 1.8 Hz, 7‐CH_Indolizine_), 3.81 (*s*, 3H, –OC*H*_3_), 2.42 (*s*, 3H, CH_3Methyl_). ^13^C NMR (100 MHz, CDCl_3_): *δ* 190.1 (C═O), 150.2 (6‐C_indolizine_), 141.2 (4‐C_Phenyl_), 138.3 (1‐C_Phenyl_), 133.2 (9‐C_indolizine_), 129.2 (2 C, 2‐C_Phenyl_, 6‐C_Phenyl_), 128.8 (2 C, 3‐C_Phenyl_, 5‐C_Phenyl_), 121.2 (8‐C_indolizine_), 118.8 (3‐C_indolizine_), 118.2 (2‐C_indolizine_), 114.5 (7‐C_indolizine_), 112.6 (1‐C_indolizine_), 108.1 (5‐C_indolizine_), 56.0 (–O*C*H_3_), 21.6 (–*C*H_3Methyl_). GC‐MS (70 eV, *m/z*, relative abundance %): 265(72); 174(100); 119(17); 65(09). mp: 156.0°C–157.7°C. HRAM‐MS (ESI+) calc for C_17_H_16_NO_2_ [M + H], 266.1175; found, 266.1180. Purity (Equipment 1, Method A): 98.9% (8.81 min).

**(6‐methoxyindolizin‐1‐yl)(4‐methoxyphenyl)methanone** (**7d**): A pale green solid was obtained in 60% yield, using α‐chloroaldehyde and **5d**. Rf: 0.41 (7:3 hexanes:ethyl acetate). ^1^H NMR (400 MHz, CDCl_3_): *δ* 8.34 (*d*, 1H, *J* = 9.7 Hz, 8‐CH_Indolizine_), 7.84 (*d*, 2H, *J* = 8.7 Hz, 2‐CH_Phenyl_, 6‐CH_Phenyl_), 7.59 (*s*, 1H, 5‐CH_Indolizine_), 7.17 (*d*, 1H, *J* = 2.7 Hz, 3‐CH_Indolizine_), 7.01 (*d*, 1H, *J* = 2.7 Hz, 2‐CH_Indolizine_), 6.98–6.91 (*m*, 3H, 3‐CH_Phenyl_, 5‐CH_Phenyl_, 7‐CH_Indolizine_), 3.87 (*s*, 3H, –OCH_3indolizine_), 3,81 (*s*, 3H, –OCH_3phenyl_). ^13^C NMR (100 MHz, CDCl_3_): *δ* 189.0 (C═O), 161.9 (4‐C_Phenyl_), 150.1 (6‐C_indolizine_), 133.5 (9‐C_indolizine_), 131.2 (2 C, 2‐C_Phenyl_, 4‐C_Phenyl_), 121.1 (1‐C_phenyl_), 118.6 (8‐C_indolizine_), 118.0 (2‐C_indolizine_), 114.4 (2 C, 3‐C_Phenyl_, 5‐C_Phenyl_), 113.4 (7‐C_indolizine_), 112.5 (1‐C_indolizine_), 108.0 (5‐C_indolizine_), 55.9 (–OCH_3indolizine_), 55.4 (–OCH_3Phenyl_). GC‐MS (70 eV, *m/z*, relative abundance %): 281(90); 174(100); 119(10); 77(08). mp: 148.1°C–149.7°C. HRAM‐MS (ESI+) calc for C_17_H_15_NO_3_ [M + H], 282.1124; found, 282.1128. Purity (Equipment 1, Method A): 98.7% (9.3 min).

**(6‐methoxyindolizin‐1‐yl)(3,4‐dichlorophenyl)methanone** (**7e**): A pale yale solid was obtained in 12% yield, using α‐chloroaldehyde and **5e**. Rf: 0.56 (7:3 hexanes:ethyl acetate). ^1^H NMR (400 MHz, CDCl_3_): *δ* 88.35 (s, 1H, 5‐CH_Indolizine_), 8.22 (d, *J *= 9.7 Hz, 1H, 8‐CH_Indolizine_), 7.89 (s, 1H, 1‐CH_phenyl_), 7.79 (d, *J* = 8.1 Hz, 1H, 6‐CH_phenyl_), 7.70 (d, *J* = 8.1 Hz, 1H, 3‐CH_indolizine_), 7.58 (d, *J* = 2.7 Hz, 1H, 5‐CH_phenyl_), 7.16 (d, *J *= 9.9 Hz, 1H, 7‐CH_indolizine_), 6.99 (d, *J* = 2.8 Hz, 1H, 2‐CH_indolizine_), 3.82 (s, 3H, –OCH_3_). ^13^C NMR (100 MHz, CDCl_3_): *δ* 185.4 (C═O), 149.7 (6‐C_indolizine_), 141.0 (9‐C_indolizine_), 133.4 (1‐C_phenyl_), 132.8 (4‐C_phenyl_), 131.3 (3‐C_phenyl_), 130.7 (2‐C_phenyl_), 130.2 (5‐C_phenyl_), 128.6 (6‐C_phenyl_), 119.8 (3‐C_indolizine_), 117.5 (8‐C_indolizine_), 116.1 (2‐C_indolizine_), 110.6 (7‐C_indolizine_), 109.9 (5‐C_indolizine_), 99.6 (6‐C_indolizine_), 56.0 (‐OC_Methoxy_). GC‐MS (70 eV, *m/z*, relative abundance %): 319 (50); 321(33); 174(100); 119 (12). HRAM‐MS (ESI+) calc for C_16_H_12_Cl_2_NO_2_ [M + H], 320.0247; found, 320.0245. Purity (Equipment 1, Method A): 99.8% (9.6 min).

**(6‐methoxyindolizin‐1‐yl)(3,4,5‐trimethoxyphenyl)methanone** (**7f**): A green solid was obtained in 65% yield, using α‐chloroaldehyde and **5f**. Rf: 0.36 (7:3 hexanes:ethyl acetate). ^1^H NMR (400 MHz, CDCl_3_): *δ* 8.36 (d, *J *= 9.7 Hz, 1H, 8‐CH_indolizine_), 7.63 (d, *J *= 2.1 Hz, 1H, 5‐CH_indolizine_), 7.21 (d, *J *= 3.0 Hz, 1H, 3‐CH_indolizine_), 7.12 (s, 2H, 2‐CH_phenyl_, 6‐CH_phenyl_), 7.07 (d, *J *= 3.0 Hz, 1H, 2‐CH_indolizine_), 7.00 (dd, *J *= 9.7, 2.2 Hz, 1H, 7‐CH_indolizine_), 3.94 (s, 3H, 4‐OCH_3Phenyl_), 3.91 (s, 6H, 3‐OCH_3Phenyl_, 5‐OCH_3Phenyl_), 3.85 (s, 3H, 6‐OCH_3Indolizine_).^13^C NMR (100 MHz, CDCl_3_): *δ* 189.2 (C═O), 152.9 (3‐C_Phenyl_, 5‐C_Phenyl_), 150.3 (6‐C_indolizine_), 140.4 (4‐C_phenyl_), 136.4 (9‐C_indolizine_), 133.7 (1‐C_phenyl_), 121.1 (3‐C_indolizine_), 118.9 (8‐C_indolizine_), 118.0 (2‐C_indolizine_), 114.6 (7‐C_indolizine_), 112.2 (1‐C_indolizine_), 108.2 (5‐C_indolizine_), 106.5 (2‐C_Phenyl_, 6‐C_Phenyl_), 61.0 (4‐OCH_3phenyl_), 56.3 (3‐OCH_3phenyl_, 5‐OCH_3phenyl_), 56.1 (3‐OCH_3indolizine_). GC‐MS (70 eV, *m/z*, relative abundance %): 341(92); 298(18); 174(100); 119(12). mp: 182.3°C–184.4°C. HRAM‐MS (ESI+) calc for C_19_H_20_NO_5_ [M + H], 342.1335; found, 342.1336. Purity (Equipment 1, Method A): 99.8% (6.0 min).

**(6‐methoxy‐2‐methylindolizin‐1‐yl)(3,4,5‐trimethoxyphenyl)methanone** (**8a**): A light green solid was obtained in 53% yield, using 1‐chloropropan‐2‐one and **5f**. Rf: 0.26 (7:3 hexanes:ethyl acetate). ^1^H NMR (300 MHz, DMSO‐d_6_) *δ* 8.14 (*d*, *J* = 1.9 Hz, 1H, 5‐CH_Indolizine_), 7.41 (*s*, 1H, 3‐CH_Indolizine_), 7.29 (*d*, *J* = 9.8 Hz, 1H, 8‐CH_Indolizine_), 6.88–6.68 (m, 3H. 7‐CH_Indolizine_, 2‐CH_trimethoxybenzene_, 6‐CH_trimethoxybenzene_), 3.76 (*sl*, 9H, 3‐OCH_3Indolizine_, 4‐OCH_3Indolizine_, 5‐OCH_3Indolizine_), 3.74 (s, 3H, 6‐OCH_3Indolizine_), 2.17 (s, 3H, –CH_3_). ^13^C NMR (75 MHz, DMSO‐ d_6_) *δ* 189.3 (C═O), 152.7 (3‐C_trimethoxybenzene_, 5‐C_trimethoxybenzene_), 148.7 (6‐C_indolizine_), 139.7(4‐C_trimethoxybenzene_), 137.3 (9‐C_indolizine_), 132.6 (1‐C_trimethoxybenzene_), 126.4 (2‐C_indolizine_), 118.7 (3‐C_indolizine_), 117.4 (8‐C_indolizine_), 115.8 (7‐C_indolizine_), 111.4 (1‐C_indolizine_), 108.8 (2‐C_trimethoxybenzene_, 6‐C_trimethoxybenzene_), 105.5 (5‐C_indolizine_), 60.2 (4‐OCH_3trimethoxybenzene_), 56.0 (3‐OCH_3trimethoxybenzene_, 5‐OCH_3trimethoxybenzene_), 55.9 (6‐OCH_3indolizine_), 12.8 (C_mehtyl_). GC‐MS (70 eV, *m/z*, relative abundance,%): 355 (100), 354 (46), 340 (46), 188 (64). mp: 127.4°C–129.3°C. HRAM‐MS (ESI+) calc for C_20_H_21_NO_5_ [M + H], 356.1495; found, 356.1496. Purity (Equipment 1, Method A): 96.2% (8.7 min).

**[2‐(Tert‐Butyl)‐6‐Methoxyindolizin‐1‐yl](3,4,5‐Trimethoxyphenyl)Methanone** (**8b**): A brown oil was obtained in 31% yield, using 1‐bromo‐3,3‐dimethylbutan‐2‐one and **5f**. Rf: 0.61 (7:3 hexanes:ethyl acetate). ^1^H NMR (400 MHz, DMSO‐d_6_) *δ* 9.10 (*d*, *J* = 2.2 Hz, 1H, 5‐CH_Indolizine_), 7.53 (*d*, *J* = 9.2 Hz, 1H, 8‐CH_Indolizine_), 6.95 (*dd*, *J* = 9.5, 2.2 Hz, 1H, 7‐CH_Indolizine_), 6.66 (s, 2H, 2‐CH_trimethoxybenzene_, 6‐CH_trimethoxybenzene_), 6.52 (s, 1H, 3‐CH_Indolizine_), 3.81 (s, 3H, 6‐OCH_3Indolizine_), 3.79 (s, 6H, 3‐OCH_3Indolizine_, 5‐OCH_3Indolizine_), 3.68 (s, 3H, 4‐OCH_3Indolizine_), 1.29 (s, 9H, 3x‐CH_3_). ^13^C NMR (100 MHz, DMSO‐d_6_): 170.4 (C═O), 160.7 (6‐C_indolizine_), 152.0 (3‐C_trimethoxybenzene_, 5‐C_trimethoxybenzene_), 136.6 (4‐C_trimethoxybenzene_), 136.4 (9‐C_indolizine_), 132.5 (1‐C_trimethoxybenzene_), 132.4 (2‐C_indolizine_), 118.8 (3‐C_indolizine_), 116.9 (8‐C_indolizine_), 111.9 (7‐C_indolizine_), 108.9 (1‐C_indolizine_), 107.1 (2‐C_trimethoxybenzene_, 6‐C_trimethoxybenzene_), 103.5 (5‐C_indolizine_), 79.5 (6‐OCH_3indolizine_), 60.0 (4‐OCH_3trimethoxybenzene_), 55.8 (3‐OCH_3trimethoxybenzene_, 5‐OCH_3trimethoxybenzene_), 27.7 (3x‐C_Methyl_). GC‐MS (70 eV, *m/z*, relative abundance %): 397 (100), 380 (32), 354 (46), 195 (44). HRAM‐MS (ESI+) calc for C_23_H_27_NO_5_ [M + H], 398.1976; found, 398.1972. Purity (Equipment 2, Method B): 98.9% (21.8 min).

**(2‐ethoxy‐6‐methoxyindolizin‐1‐yl)(3,4,5‐trimethoxyphenyl)methanone (8c)**: A yellow solid was obtained in 28% yield, using ethyl 2‐bromoacetate and **5f**. Rf: 0.65 (7:3 hexanes:ethyl acetate). ^1^H NMR (300 MHz, DMSO‐d_6_) *δ* 9.14 (*d*, 1H, *J* = 1.2 Hz, 5‐CH_indolizine_), 7.60 (*d*, 1H, *J* = 9.3 Hz, 8‐CH_indolizine_), 7.01 (*dd*, 1H, *J* = 2.1, 9.6 Hz, 7‐CH_indolizine_), 6.73 (*s*, 2H, 2‐CH_phenyl_, 6‐CH_phenyl_), 6.59 (*s*, 1H, 3‐CH_indolizine_), 4.14 (*q*, 2H, *J* = 6.9 Hz, –OCH_2ethoxy_), 3.82 (*s*, 3H, 4‐OCH_3Phenyl_), 3.78 (*s*, 6H, 3‐OCH_3Phenyl_, 5‐OCH_3Phenyl_), 3.69 (*s*, 3H, 6‐OCH_3Indolizine_), 1.05 (*t*, 3H, *J* = 7.2 Hz, –CH_3ethoxy_). ^13^C NMR (75 MHz, DMSO‐d_6_): *δ* 161.2 (C═O), 152.0 (3‐C_trimethoxybenzene_, 5‐C_trimethoxybenzene_), 149.3 (6‐C_indolizine_), 136.7 (4‐C_trimethoxybenzene_), 136.4 (9‐C_indolizine_), 132.9 (1‐C_trimethoxybenzene_), 131.8 (2‐C_indolizine_), 119.0 (3‐C_indolizine_), 117.3 (8‐C_indolizine_), 110.6 (7‐C_indolizine_), 109.3 (1‐C_indolizine_), 107.3 (2‐C_trimethoxybenzene_, 6‐C_trimethoxybenzene_), 104.0 (5‐C_indolizine_), 60.1 (‐CH_2ethoxy_), 59.2 (4‐OCH_3trimethoxybenzene_), 55.8 (3‐OCH_3trimethoxybenzene_, 5‐OCH_3trimethoxybenzene_), 55.71 (6‐OCH_3indolizine_), 13.9 (–CH_3ethoxy_). GC‐MS (70 eV, *m/z*, relative abundance %): 385 (100), 370 (34), 313 (25), 298 (25). mp: 153.2°C–156.2°C. HRAM‐MS (ESI+): calc for C_21_H_23_NO_6_Na [M+Na], 408.1408, found, 408.1410. Purity (Equipment 1, Method A): 98.4% (10.1 min).

**Ethyl [6‐methoxy‐1‐(3,4,5‐trimethoxybenzoyl)indolizine]‐2‐carboxylate (8d)**: A light green solid was obtained in 46% yield, using ethyl 3‐bromo‐2‐oxopropanoate and **5f**. Rf: 0.19 (7:3 hexanes:ethyl acetate). ^1^H NMR (300 MHz, DMSO‐d_6_) *δ* 8.23 (*d*, 1H, *J* = 1.8 Hz, 5‐CH_indolizine_), 8.05 (*s*, 1H, 3‐CH_indolizine_), 7.75 (*d*, 1H, *J* = 9.9 Hz, 8‐CH_indolizine_), 7.02 (*dd*, 1H, *J* = 2.1, 9.9 Hz, 7‐CH_indolizine_), 6.95 (*s*, 2H, 2‐CH_phenyl_, 6‐CH_phenyl_), 3.80–3.78 (*m*, 5H, CH_2Ethyl_, 4‐OCH_3Phenyl_), 3.75 (*s*, 6H, 3‐OCH_3Phenyl_, 5‐OCH_3Phenyl_), 3.71 (*s*, 3H, 6‐OCH_3Indolizine_), 0.843 (*t*, 3H, *J* = 7.2 Hz, CH_3ethyl_). ^13^C NMR (75 MHz, DMSO‐d_6_): *δ* 188.6 (C═O_Ketone_), 164.2 (C═O_ester_), 152.5 (3‐C_trimethoxybenzene_, 5‐C_trimethoxybenzene_), 149.6 (6‐C_indolizine_), 140.6 (4‐C_trimethoxybenzene_), 136.1(9‐C_indolizine_), 132.3 (1‐C_trimethoxybenzene_), 120.6 (2‐C_indolizine_), 119.8 (3‐C_indolizine_), 119.4 (8‐C_indolizine_), 118.4 (7‐C_indolizine_), 110.7 (1‐C_indolizine_), 108.5 (2‐C_trimethoxybenzene_, 6‐C_trimethoxybenzene_), 106.1 (5‐C_indolizine_), 60.3 (–OCH_2ethoxy_), 60.1 (4‐OCH_3trimethoxybenzene_), 56.0 (3‐OCH_3trimethoxybenzene_, 5‐OCH_3trimethoxybenzene_), 55.8 (6‐OCH_3indolizine_), 13.4 (‐CH_3ethoxy_). GC‐MS (70 eV, *m/*z, relative abundance %): 414 (26), 413 (100), 246 (27), 218 (45). mp: 141.7°C–143.9°C. HRAM‐MS (ESI+) calc for C_22_H_23_NO_7_Na [M+Na], 436.1354, found, 436.1355. Purity (Equipment 1, Method A): 97.9% (8.8 min).

**[6‐methoxy‐2‐(trifluoromethyl)indolizin‐1‐yl](3,4,5‐trimethoxyphenyl)methanone (8e)**: A green oil was obtained in 35% yield, using 3‐bromo‐1,1,1‐trifluoropropan‐2‐one and **5f**. Rf: 0.53 (6:4 hexanes:ethyl acetate). ^1^H NMR (300 MHz, DMSO‐d_6_) *δ* 8.23 (s, 1H, 5‐CH_indolizine_), 8.17 (s, 1H, 3‐CH_indolizine_) 6.97 (*sl*, 4H, 8‐CH_indolizine_, 7‐CH_indolizine_, 2‐CH_phenyl_, 6‐CH_phenyl_), 3.78 (*s*, 3H, 4‐OCH_3Phenyl_), 3.76 (*s*, 3H, 6‐OCH_3Indolizine_), 3.7 (*s*, 6H, 3‐OCH_3Phenyl_, 5‐OCH_3Phenyl_). ^13^C NMR (75 MHz, DMSO‐d_6_): *δ* 187.2 (C═O), 152.7 (3‐C_trimethoxybenzene_, 5‐C_trimethoxybenzene_), 149.2 (6‐C_indolizine_), 140.9 (4‐C_trimethoxybenzene_), 135.2 (9‐C_indolizine_), 132.3 (1‐C_trimethoxybenzene_), 119.9 (2‐C_indolizine_), 119.3 (3‐C_indolizine_), 117.8 (8‐C_indolizine_), 117.3 (CF_3_), 117.1 (7‐C_indolizine_), 109.4 (1‐C_indolizine_), 108.9 (2‐C_trimethoxybenzene_, 6‐C_trimethoxybenzene_), 106.3 (5‐C_indolizine_), 60.3 (4‐OCH_3trimethoxybenzene_), 56.0 (3‐OCH_3trimethoxybenzene_, 5‐OCH_3trimethoxybenzene_), 55.9 (6‐OCH_3indolizine_). GC‐MS (70 eV, *m/z*, relative abundance %): 410 (22), 409 (100), 366 (22), 241 (97). mp: 182.3°C–184.4°C. HRAM‐MS (ESI+) calc for C_20_H_18_F_3_NO_5_ [M + H], 410.1221; found, 410.1218. Purity (Equipment 1, Method A): 96.4% (9.3 min).

**(6‐methoxy‐2‐phenylindolizin‐1‐yl)(3,4,5‐trimethoxyphenyl)methanone** (**8f**): A green oil was obtained in 35% yield, using 2‐bromo‐1‐phenylethanone and **5f**. Rf: 0.32 (7:3 hexanes:ethyl acetate). ^1^H NMR (300 MHz, CDCl_3_) *δ* 8.28 (*d*, 1H, *J* = 9.9 Hz, 8‐CH_indolizine_), 7.59 (*d*, 1H, *J* = 1.8 Hz, 5‐CH_indolizine_), 7.17–7.07 (*m*, 6H, 3‐CH_indolizine_, 2‐CH_phenyl_, 3‐CH_phenyl_, 4‐CH_phenyl_, 5‐CH_phenyl_, 6‐CH_phenyl_), 6.91 (*dd*, 1H, *J* = 2.1, 9.6 Hz, 7‐CH_indolizine_), 6.83 (*s*, 2H, 2‐CH_trimethoxybenzene_, 6‐CH_trimethoxybenzene_), 3.83 (*s*, 3H, 4‐OCH_3trimethoxybenzene_), 3.84 (*s*, 3H, 6‐OCH_3Indolizine_), 3.66 (*s*, 6H, 3‐OCH_3trimethoxybenzene_, 5‐OCH_3trimethoxybenzene_). ^13^C NMR (75 MHz, CDCl_3_): δ 190.8 (C═O), 152.4 (3‐C_trimethoxybenzene_, 5‐C_trimethoxybenzene_), 150.2 (6‐C_indolizine_), 140.7 (4‐C_trimethoxybenzene_), 135.5 (9‐C_indolizine_), 135.0 (2‐C_indolizine_), 134.5 (3‐C_indolizine_), 132.1 (1‐C_trimethoxybenzene_), 129.5 (2‐C_Phenyl_, 6‐C_phenyl_), 128.0 (3‐C_Phenyl_, 5‐C_phenyl_), 126.8 (4‐C_phenyl_), 120.7 (8‐C_indolizine_), 118.3 (7‐C_indolizine_), 113.9 (1‐C_Phenyl_), 110.9 (1‐C_indolizine_), 107.6 (5‐C_indolizine_), 107.3 (2‐C_trimethoxybenzene_, 6‐C_trimethoxybenzene_), 60.9 (4‐OCH_3trimethoxybenzene_), 56.1 (3‐OCH_3trimethoxybenzene_, 5‐OCH_3trimethoxybenzene_), 56.1 (6‐OCH_3indolizine_). GC‐MS (70 eV, *m/z*, relative abundance %): 418 (28), 417 (100), 250 (71), 207 (15). HRAM‐MS (ESI+) calc for C_25_H_23_NO_5_ [M + H], 418.1645; found, 418.1644. Purity (Equipment 1, Method A): 96.9% (9.5 min).

**[6‐methoxy‐2‐(pyridin‐2‐yl)indolizin‐1‐yl](3,4,5‐trimethoxyphenyl)methanone** (**8g**): A brown oil was obtained in 30% yield, using 2‐bromo‐1‐(pyridin‐2‐yl)ethanone and **5f**. Rf: 0.28 (6:4 hexanes:ethyl acetate). ^1^H NMR (300 MHz, DMSO‐d_6_) *δ* 8.31 (*d*, 1H, *J* = 4.8 Hz, 5‐CH_indolizine_), 8.26 (*d*, 1H, *J* = 1.8 Hz, 4‐CH_pyridine_), 7.90 (*s*, 1H, 3‐CH_indolizine_), 7.83 (*d*, 1H, *J* = 9.9 Hz, 8‐CH_indolizine_), 7.52 (*td*, 1H, *J* = 1.8, 7.8 Hz, 5‐CH_pyridine_), 7.19 (*d*, 1H, *J* = 7.8 Hz, 6‐CH_pyridine_), 7.06 (*ddd*, 1H, *J* = 0.9, 4.8, 7.2 Hz, 3‐CH_pyridine_), 7.00 (*dd*, 1H, *J* = 2.4, 9.9 Hz, 7‐CH_indolizine_), 6.75 (*s*, 2H, 2‐CH_trimethoxybenzene_, 6‐CH_trimethoxybenzene_), 3.82 (*s*, 3H, 4‐OCH_3trimethoxybenzene_), 3.58 (*sl*, 6H, 3‐OCH_3trimethoxybenzene_, 5‐OCH_3trimethoxybenzene_), 3.36 (*s*, 3H, 6‐OCH_3Indolizine_). ^13^C NMR (75 MHz, DMSO – d_6_) *δ* 189.7 (C═O), 153.8 (3‐C_trimethoxybenzene_, 5‐C_trimethoxybenzene_), 152.4 (2‐C_pyridine_), 149.7 (6‐C_indolizine_), 149.3 (3‐C_pyridine_, 6‐C_pyridine_), 140.3 (4‐C_trimethoxybenzene_), 136.1 (9‐C_indolizine_), 136.0 (4‐C_pyridine_), 133.3 (1‐C_trimethoxybenzene_), 130.9 (3‐C_indolizine_), 124.5 (2‐C_indolizine_), 121.6 (5‐C_indolizine_), 119.7 (8‐C_indolizine_), 119.0 (7‐C_indolizine_), 115.9 (5‐C_pyridine_), 110.5 (1‐C_indolizine_), 109.1 (5‐C_indolizine_), 107.0 (2‐C_trimethoxybenzene_, 6‐C_trimethoxybenzene_), 60.4 (4‐OCH_3trimethoxybenzene_), 56.4 (3‐OCH_3trimethoxybenzene_, 5‐OCH_3trimethoxybenzene_), 56.0 (6‐OCH_3indolizine_). GC‐MS (70 eV, *m/z*, relative abundance %): 18 (100), 359 (22), 251 (70), 208 (31). HRAM‐MS (ESI+) calc for C_24_H_22_N_2_O_5_ [M + H], 419.1612; found, 419.1602. Purity (Equipment 1, Method A): 99.8% (6.5 min).

**(6‐methoxy‐2‐propylindolizin‐1‐yl)(3,4,5‐trimethoxyphenyl)methanone (8h)**: A yellow oil was obtained in 38% yield, using 1‐bromopentan‐2‐one and **5f**. Rf: 0.59 (6:4 hexanes:ethyl acetate). ^1^H NMR (300 MHz, DMSO‐d_6_) *δ* 8.14 (d, *J* = 1.9 Hz, 1H, 5‐CH_indolizine_), 7.44 (s, 1H, 3‐CH_indolizine_), 7.15 (d, *J* = 9.8 Hz, 1H, 8‐CH_indolizine_), 6.87 (s, 2H, 2‐CH_trimethoxybenzene_, 6‐CH_trimethoxybenzene_), 6.86–6.81 (dd, *J* = 2.0, 9.8 Hz, 1H, 7‐CH_indolizine_), 3.75 (m, 12H, 3‐OCH_3trimethoxybenzene_, 4‐OCH_3trimethoxybenzene_, 5‐OCH_3trimethoxybenzene_, 6‐OCH_3indolizine_), 2.64–2.57 (t, *J* = 7.4, 2H, 1‐CH_2propyl_), 1.54 (*s*, *J* = 7.4, 2H, 2‐CH_2propyl_), 0.84 (t, *J* = 7.3 Hz, 3‐CH_3propyl_). ^13^C NMR (75 MHz, DMSO‐d_6_) *δ* 189.3 (C═O), 152.7 (3‐C_trimethoxybenzene_, 5‐C_trimethoxybenzene_), 148.6 (6‐C_indolizine_), 139.8 (4‐C_trimethoxybenzene_), 137.2 (9‐C_indolizine_), 132.5 (1‐C_trimethoxybenzene_), 131.6 (2‐C_indolizine_), 118.7 (3‐C_indolizine_), 117.2 (8‐C_indolizine_), 115.1 (7‐C_indolizine_), 110.8 (1‐C_indolizine_), 108.8 (2‐C_trimethoxybenzene_, 6‐C_trimethoxybenzene_), 105.6 (5‐C_indolizine_), 60.2 (4‐OCH_3trimethoxybenzene_), 56.0 (3‐OCH_3trimethoxybenzene_, 5‐OCH_3trimethoxybenzene_), 55.9 (6‐OCH_3indolizine_), 28.6 (1‐C_propyl_), 23.5 (2‐C_propyl_), 14.0 (3‐C_propyl_). GC‐MS (70 eV, *m/z*, relative abundance %): 383 (100), 368 (50), 354 (29), 195 (57). HRAM‐MS (ESI+) calc for C_24_H_22_N_2_O_5_ [M + H], 384.1812; found, 384.1814 Purity (Equipment 1, Method A): 98.8% (9.6 min).

**(6‐methoxy‐2‐pentylindolizin‐1‐yl)(3,4,5‐trimethoxyphenyl)methanone (8i)**: A brown oil was obtained in 60% yield, using 1‐bromoheptan‐2‐one and **5f**. Rf: 0.37 (6:4 hexanes:ethyl acetate). ^1^H NMR (300 MHz, CDCl_3_): *δ* 7.51 (*d*, 1H, *J* = 2.4 Hz, 5‐CH_indolizine_), 7.29 (*d*, 1H, *J* = *12.8 *Hz, 8‐CH_indolizine_), 7.95 (*s*, 2H, 2‐CH_trimethoxybenzene_, 6‐CH_trimethoxybenzene_) 6.67 (*dd*, 1H, *J* = 12.8, 2.8 Hz, 7‐CH_indolizine_), 3.90 (*s*, 3H, 4‐OCH_3trimethoxybenzene_), 3.82 (*s*, 6H, 3‐OCH_3trimethoxybenzene_, 5‐OCH_3trimethoxybenzene_), 3.78 (*s*, 3H, 6‐OCH_3indolizine_), 2.71 (*t*, 3H, *J* = 10 Hz, 1‐CH_2pentyl_) 1.56 (*q*, 2H, *J* = 9,6 Hz, 2‐CH_2pentyl_) 1.27–1.23 (*m*, 4H, 3‐CH_2pentyl_, 4‐CH_2pentyl_), 0.84 (*t*, 3H, *J* = 9.2 Hz, 5‐CH_3pentyl_).^13^C NMR (75 MHz, CDCl_3_): *δ* 190.7 (C═O), 152.9 (3‐C_trimethoxybenzene_, 5‐C_trimethoxybenzene_), 149.3 (6‐C_indolizine_), 140.6 (4‐C_trimethoxybenzene_), 137.1 (9‐C_indolizine_), 133.5 (1‐C_trimethoxybenzene_), 119.8 (3‐C_indolizine_), 116.9 (8‐C_indolizine_), 114.4 (2‐C_indolizine_), 111.8 (7‐C_indolizine_), 107.8 (5‐C_indolizine_), 106.3 (2‐C_trimethoxybenzene_, 6‐C_trimethoxybenzene_), 61.0 (4‐OCH_3trimethoxybenzene_), 56.2 (3‐OCH_3trimethoxybenzene_, 5‐OCH_3trimethoxybenzene_), 56.0 (6‐OCH_3indolizine_), 31.8 (1‐C_pentyl_), 30.5 (2‐C_pentyl_), 26.9 (3‐C_pentyl_), 22.5 (4‐C_pentyl_), 14.0 (5‐C_pentyl_). GC‐MS (70 eV, *m/z*, relative abundance %): 411(100); 340(43); 354(100); 216(32); 195(76). HRAM‐MS (ESI+) calc for C_24_H_30_NO_5_ [M + H], 412.2118; found, 412.2109. Purity (Equipment 1, Method A): 98.4% (9.8 min).

**[6‐methoxy‐2‐(** ***p*** **‐tolyl)indolizin‐1‐yl](3,4,5‐trimethoxyphenyl)methanone (8j)**: A yellow solid was obtained in 58% yield, using 2‐bromo‐1‐(*p*‐tolyl)ethanone and **5f**. Rf: 0.67 (1:1 hexanes:ethyl acetate). ^1^H NMR (500 MHz, DMSO‐d_6_): *δ* 8.23 (*s*, 1H, 5‐CH_indolizine_), 7.87 (*d*, 1H, *J* = 7.6 Hz, 8‐CH_indolizine_), 7.66 (*s*, 1H, 3‐CH_indolizine_), 7.02–6.99 (*m*, 3H, 7‐CH_indolizine_, 2‐CH_phenyl_, 6‐CH_phenyl_), 6.94 (*d*, 2H, *J* = 6.4 Hz, 3‐CH_phenyl_, 5‐CH_phenyl_), 6.73 (*s*, 2H, 2‐CH_trimethoxybenzene_, 6‐CH_trimethoxybenzene_), 3.81 (*s*, 3H, 4‐OCH_3trimethoxybenzene_), 3.59 (*s*, 6H, 3‐OCH_3trimethoxybenzene_, 5‐OCH_3trimethoxybenzene_), 3.56 (*s*, 3H, 6‐OCH_3indolizine_), 2.18 (*s*, 3H, ‐CH_3Methyl_).^13^C NMR (100 MHz, CDCl_3_): *δ* 189.2 (C═O), 151.9 (3‐C_trimethoxybenzene_, 5‐C_trimethoxybenzene_), 149.2 (6‐C_indolizine_), 140.0 (4‐C_trimethoxybenzene_), 135.3 (9‐C_indolizine_), 133.1 (1‐C_trimethoxybenzene_), 131.8 (2‐C_indolizine_), 130.9 (1‐C_phenyl_), 128.9 (2‐C_Phenyl_, 6‐C_Phenyl_), 128.2 (3‐C_Phenyl_, 5‐C_Phenyl_), 119.2 (3‐C_indolizine_), 118.1 (8‐C_indolizine_), 114.2 (7‐C_indolizine_), 109.7 (4‐C_Phenyl_), 108.7 (5‐C_indolizine_), 106.8 (2‐C_trimethoxybenzene_, 6‐C_trimethoxybenzene_), 60.0 (4‐OCH_3trimethoxybenzene_), 55.6 (3‐OCH_3trimethoxybenzene_, 5‐OCH_3trimethoxybenzene_), 55.6 (6‐OCH_3indolizine_), 20.5 (‐*C*H_3_). GC‐MS (70 eV, *m/z*, relative abundance %): 431(100); 264(72); 216(11). mp: 159.8°C–162.8°C. HRAM‐MS (ESI+) calc for C_26_H_26_NO_5_ [M + H], 432.1805; found, 432.1783. Purity (Equipment 1, Method A): 95.1% (10.4 min).

**[6‐methoxy‐2‐(4‐methoxyphenyl)indolizin‐1‐yl](3,4,5‐trimethoxyphenyl)methanone (8k)**: A yellow solid was obtained in 55% yield, using 2‐bromo‐1‐(4‐methoxyphenyl)ethanone and **5f**. Rf: 0.57 (1:1 hexanes:ethyl acetate). ^1^H NMR (500 MHz, DMSO‐d_6_): *δ* 8.22 (*d*, 1H, *J* = 1.2 Hz, 8‐CH_indolizine_), 7.87 (*d*, 1H, *J* = 7.6 Hz, 5‐CH_indolizine_), 7.63 (*s*, 1H, 3‐CH_indolizine_), 7.05 (*d*, 2H, *J* = 6.8 Hz, 2‐CH_phenyl_, 6‐CH_phenyl_), 7.00 (*dd*, 1H, *J* = *8.0*, 2.0 Hz, 7‐CH_indolizine_), 6.73 (*s*, 2H, 2‐CH_trimethoxybenzene_, 6‐CH_trimethoxybenzene_), 6.70 (*d*, 2H, *J* = 6.8 Hz, 3‐CH_phenyl_, 5‐CH_phenyl_), 3.81 (*s*, 3H, 4‐OCH_3trimethoxybenzene_), 3.66 (*s*, 3H, 4‐OCH_3trimethoxybenzene_), 3.60 (*s*, 6H, 3‐OCH_3trimethoxybenzene_, 5‐OCH_3trimethoxybenzene_), 3.57 (*s*, 3H, 6‐OCH_3indolizine_), 3.31 (*s*, 3H, 4‐OCH_3phenyl_).^13^C NMR (125 MHz, CDCl_3_): *δ* δ 189.2 (C═O), 157.9 (3‐C_trimethoxybenzene_, 5‐C_trimethoxybenzene_), 151.9 (6‐C_indolizine_), 149.2 (4‐C_phenyl_), 140.0 (4‐C_trimethoxybenzene_), 135.3 (9‐C_indolizine_), 133.1 (1‐C_trimethoxybenzene_), 130.7 (2‐C_indolizine_), 130.2 (2‐C_Phenyl_, 6‐C_Phenyl_), 127.1 (1‐C_phenyl_), 119.2 (3‐C_indolizine_), 118.0 (8‐C_indolizine_), 114.1 (7‐C_indolizine_), 113.3 (1‐C_indolizine_), 109.7 (3‐C_Phenyl_, 5‐C_Phenyl_), 108.6 (5‐C_indolizine_), 106.8 (2‐C_trimethoxybenzene_, 6‐C_trimethoxybenzene_), 59.9 (4‐OCH_3trimethoxybenzene_), 55.9 (3‐OCH_3trimethoxybenzene_, 5‐OCH_3trimethoxybenzene_), 55.6 (6‐OCH_3indolizine_), 55.0 (4‐OCH_3Phenyl_). GC‐MS (70 eV, *m/z*, relative abundance %): 447(100); 280(65); 124(9). mp: 138.1°C–140.5°C. HRAM‐MS (ESI+) calc for C_26_H_26_NO_6_ [M + H], 448.1754; found, 448.1738. Purity (Equipment 1, Method A): 96.1% (9.4 min).

### 4.2 Pharmacological/Biological Assays

#### 4.2.1 Cell Culture

The tongue squamous cell carcinoma cell line CAL‐27 (ATCC CRL‐2095), breast carcinoma BT‐20 (ATCC HTB‐19), gastric carcinoma HGC‐27 (BCRJ Code 0310), and normal oral fibroblast OHMF (culture of fibroblasts derived from human oral healthy mucosa, approved by the Research Ethics Committee of the University of São Paulo, Campus of Ribeirão Preto, Brazil (with protocol number 049) [30] were cultured in DMEM medium (DMEM, Sigma‐Aldrich, St. Louis, MO, USA) supplemented with 10% fetal bovine serum and a solution of penicillin (100 U/mL) and streptomycin (100 μg/mL). The cultures were maintained in a humidified incubator with 5% CO₂ at 37°C.

#### 4.2.2 Cell Viability Assay

Cell viability was analyzed using the resazurin assay. For this, cells were seeded in 96‐well plates (1 × 10^3^ cells per well) and incubated at 37°C with 5% CO₂ overnight for cell adhesion. Subsequently, the cells were treated with different compounds (0–50 µM) for 72 h. After treatment, the medium was replaced with phenol‐free DMEM medium (Sigma‐Aldrich) containing resazurin at a concentration of 44 µM (Sigma‐Aldrich). After incubation for 4 h at 37°C, fluorescence detection was performed (excitation 530/25 nm, emission 590/35 nm) using a microplate fluorimeter (Synergy 2, Biotek). Initial cell growth inhibition assays were performed in triplicate or quadruplicate, while IC_50_ determinations were conducted in triplicate. Data were plotted, and IC₅₀ values were determined by nonlinear regression using GraphPad Prism software 9.0.

#### 4.2.3 Tubulin Inhibition Assay

To evaluate the effect of the compounds on tubulin polymerization, the Tubulin Polymerization Assay Kit (Cytoskeleton Inc.) was used according to the manufacturer's instructions. Briefly, 5 µL of the compounds (final concentration ranging from 0.1 to 100 µM) were pipetted per well into a pre‐warmed 96‐well black‐bottom plate at 37°C. After 1 min, 55 µL of the tubulin reaction mix (1X Buffer 1, 0.7X Tubulin Glycerol Buffer, 1 mM GTP, and 2 mg/mL tubulin) was added to each well. Next, after 5 s of shaking, fluorescence detection (excitation: 340–360 nm, emission: 410–460 nm) was performed using a microplate fluorimeter. A total of 61 reading cycles were carried out at 1‐min intervals to obtain kinetic curves and calculate the Maximum Reaction Velocity (Vmax). Data were plotted, and IC₅₀ values were determined by nonlinear regression using GraphPad Prism software 9.0.

#### 4.2.4 Cell‐Cycle Assay

BT‐20 cells were seeded in 10 cm plates (1.5 × 10⁶ cells/plate) and incubated at 37°C with 5% CO₂ for 24 h. Subsequently, the cells were treated with different compounds at a concentration of 2.5 µM for 24 h. After treatment, the cells were collected by centrifugation and fixed in cold 70% ethanol for 1 h at 4°C. The cells were then washed, collected by centrifugation, and treated with RNase A (100 μg/mL, Sigma‐Aldrich) for 30 min at 37°C, followed by staining with propidium iodide (50 μg/mL) for 15 min at 37°C. Finally, the percentages of cells in each phase of the cell cycle were determined using FACSCalibur flow cytometry (BD Biosciences—San Jose, CA, USA). Cell‐cycle distribution was analyzed using ModFit LT V3.3 software.

#### 4.2.5 Western Blot

Cells were seeded in 10 cm plates (1.5 × 10⁶ cells/plate) and incubated for 24 h. Subsequently, the cells were treated with the compounds at a concentration of 2.5 µM for 24 h. After treatment, the cells were collected, and the proteins were isolated. A total of 30 µg of proteins were separated electrophoretically on a sodium dodecyl sulfate‐polyacrylamide gel (SDS‐PAGE) and transferred onto a polyvinylidene fluoride (PVDF) membrane (GE Healthcare) using the PowerBlotter Station (Invitrogen) according to the manufacturer's recommendations. After transfer, the membrane was blocked with a 5% (w/v) nonfat milk solution in TBS‐T (0.1 M Tris (pH 7.5), 0.9% NaCl, 0.1% Tween‐20) at room temperature for 1 h under gentle agitation. The membrane was then washed with TBS‐T and incubated with primary antibodies under agitation for approximately 16 h at 4°C. The primary antibodies used were: c‐Myc (#5605), ß‐tubulin (#2128), p21 (#2947), Cyclin D1 (#2978), Bcl‐2 (#15071), p‐AKT ser473 (#4058), and GAPDH (#2118) (Cell Signaling Technology). After primary antibody incubation, the membranes were washed with TBS‐T and incubated at room temperature for 1 h in a TBS‐T solution containing 5% nonfat milk and the HRP‐conjugated secondary antibody. Following the washes with TBS‐T, the membranes were incubated with the SuperSignal West Dura substrate (Thermo Scientific). Immunodetection was analyzed using the ChemiDoc imaging system (Bio‐Rad).

#### 4.2.6 Cell Preparation

HEK‐293 cell lines stably expressing human P2X7 receptor (hP2X7R‐HEK‐293, B'SYS GmbH, Witterswil, Switzerland) were maintained in a 50/50 mix of Dulbecco's Modified Eagle Medium and F12 medium (DMEM/F12) supplemented with 9% (v/v) FCS, 1% (v/v) Penicillin/Streptomycin (10000 I.U./mL, 10 mg/mL in 0.9% NaCl) and G418 (100 µg/mL) at 37°C and 5% CO_2_. Cells were subcultured in cell culture 75 cm^2^ flasks and split every 2‐4 days (1:3; 1:6; 1:10) once confluent.

Human 1321N1 astrocytoma cells stably expressing the human P2X4 receptor (generated in‐house) [31] were cultured in Dulbecco's Modified Eagle Medium supplemented with 9% (v/v) FCS, 1% (v/v) Penicillin/Streptomycin (10000 I.U./mL, 10 mg/mL in 0.9% NaCl) and puromycin (1 µg/mL) as a selection antibiotic at 37°C and 5% CO_2_. Cells were subcultured in cell culture 75 cm^2^ flasks and split every 2–4 days (1:5; 1:6) once confluent.

#### 4.2.7 YO‐PRO‐1 Dye Uptake Assay for the P2X7 Receptor

HEK‐293 cell lines stably expressing human P2X7 receptor were seeded into black‐walled Nunc 96‐well optical bottom plates (Thermo Fisher Scientific) at 2.0–4.0 × 10^4^ cells/well and incubated at 37°C for 24–48 h. After removing the culture medium, cells were washed with 100 µL wash buffer (280 mM Sucrose; 5.6 mM KCl; 0.5 mM CaCl_2_; 10 mM d‐Glucose; 10 mM HEPES; 5 mM N‐Methyl‐d‐glucamine (pH 7.4)). Then, 50 µL assay buffer (280 mM Sucrose; 6.4 mM KCl, 10 mM d‐Glucose; 10 mM HEPES; 5.0 mM N‐Methyl‐d‐glucamine (pH 7.4)) and 50 µL of antagonist dilutions (10^–5^–10^–9 ^M) in assay buffer were added, and the cells were incubated at 37°C for 30 min. Subsequently, 50 µL of YO‐PRO‐1 iodide solution (2 µM) in assay buffer and 50 µL of BzBzATP (determined EC_50_ concentration under the assay conditions) in assay buffer were added to the cells and incubated at 37°C for 2 h. Finally, the uptake of YO‐PRO‐1 dye was recorded in a 9‐point‐well scan by following the fluorescence change using a FlexStation 3 Multi‐Mode Microplate Reader (Molecular Devices, San Jose, CA, USA, Software SoftMax7 Pro, endpoint protocol, well scan with reading pattern “fill scan”, *λ*_ex_ = 485 nm, *λ*_em_ = 535 nm). To determine the concentration‐dependent uptake inhibition by antagonists, the YO‐PRO‐1 uptake, expressed as RFU, was plotted against the concentrations of the compounds.

#### 4.2.8 Ca^2+^‐Flux Assay for the hP2X4 Receptor

1321N1 cells stably expressing human P2X4 receptor were seeded into black‐walled 96‐well plates (Thermo Fisher Scientific) at 2.5 × 104 cells/well and incubated at 37°C for 24–48 h. All test compounds were diluted in assay buffer solution (SBS: 135 mM NaCl; 5 mM KCl; 1 mM MgCl_2_.6 H_2_O; 1.8 mM CaCl_2_; 5.6 mM d‐Glucose; 10 mM HEPES, 0.1% (m/v) BSA, 0.05% (m/v) gelatine, 2.3 mM probenecid). The culture medium was removed, and 100 µL of 4 μM fluorescent Ca^2+^ indicator Calbryte520AM in SBS was added and incubated at 37°C for 1 h. The dye solution was then removed, 100 µL of SBS solution and 50 µL of antagonist dilutions (10^–5^–10^–9 ^M) in SBS were added, and the cells were incubated at room temperature for an extra 30 min. Finally, 50 µL of ATP (at a concentration of the previously determined EC_50_‐ value) was added by using FlexStation 3 Multi‐Mode Microplate Reader (Molecular Devices, San Jose, CA, USA, Software SoftMax7 Pro). The changes in intracellular Ca^2+^ ion concentrations were monitored over 200 s (20 s baseline) by measuring the fluorescence signal at *λ*_ex_ = 494 nm and *λ*_em_ = 516 nm (515 nM cut‐off). The concentration‐dependent decrease in Ca^2+^‐flux, expressed as RFU, was plotted against the concentrations of the compounds.

### 4.3 Molecular Docking

Molecules were drawn using ChemDraw Ultra 12.0 software and minimized using Avogadro 2.0 software. The crystal structure of Tubulin (PDB ID: 4O2B) downloaded from the Protein Data Bank (https://www.rcsb.org) was prepared using Auto Dock Tools software. Docking was operated using AutoDock Vina software after setting the grid at the center of the Colchicine binding site with a grid size of 20 Å. The lowest binding energy for the docked conformations was chosen from all conformations as the representative binding energy to evaluate the potential of the corresponding compounds. The best docking poses were selected for analyzing the interactions between the receptor tubulin and compounds **8e**, **8h**, **7f**, **Colchicine**, and **BPR0L074**.

## Conflicts of Interest

The authors declare no conflicts of interest.

## Supporting information

Support Information Revised.

ArchPharm SupplMat InChI 24 10 revised.
