# Supplementary material for: Design, Synthesis, and Selective Antiproliferative Activity of Indolizine Derivatives as Microtubule Destabilizers
Source: Arch Pharm (Weinheim). 2025 Dec 3;358(12):e70161. doi: 10.1002/ardp.70161 (PMC12673918; doi:10.1002/ardp.70161)
Supplement: Supplementary file 1 — Support Information Revised. [file ARDP-358-e70161-s002.docx]

Support Information

Design, Synthesis, and Selective Antiproliferative Activity of New Indolizine-Based Microtubule Destabilizers

Victor Hugo Catricala Fernandes^1,3^, Maitê Bueno Giometti^1^, Franco Jazon Caires^1^, Gabriel de Paula Bueno^1^, Gabriel da Silva^2^, Andréia Machado Leopoldino^2^, Anna Junker^3^, Giuliano Cesar Clososki^1,^*

1 Research Center on Natural and Synthetic Products, Department of Biomolecular Sciences, Faculty of Pharmaceutical Sciences of Ribeirão Preto, Ribeirão Preto, Brazil.

2 Department of Clinical Analyses, Toxicological and Food Sciences, Faculty of Pharmaceutical Sciences of Ribeirão Preto, Ribeirão Preto, Brazil.

3 Werner Siemens Imaging Center, Department of Preclinical Imaging and Radiopharmacy, Cluster of Excellence iFIT (EXC 2180) “Image-guided and Functionally Instructed Tumor Therapies”,University of Tuebingen, Roentgenweg 13, 72076 Tuebingen, Germany.

*Correspondence:

Prof. Dr. Giuliano Cesar Clososki, Department of Biomolecular Sciences, Faculty of Pharmaceutical Sciences of Ribeirão Preto, Av. Prof. Zeferino Vaz, S/N, 14040-230, Ribeirão Preto, Brazil.

Email: gclososki@usp.br

Content:

[**1.** **Initial screening of intermediates (4a – 4e):** 2](#_Toc212189724)

[**2.** **Inhibition values [%] of compounds 5f, 6a - 6e, 7a - 7e, 8a – 8k at P2X7 and P2X4 receptors** 3](#_Toc212189725)

[**3.** **NMR, GC-MS, HRAM-MS data.** 4](#_Toc212189726)

1. **Initial screening of intermediates (4a – 4e):**

The initial ketone intermediates were evaluated in a screening with a concentration of 50 µM, showing some cell growth inhibition, however our mainly focus were in the indolizine core.


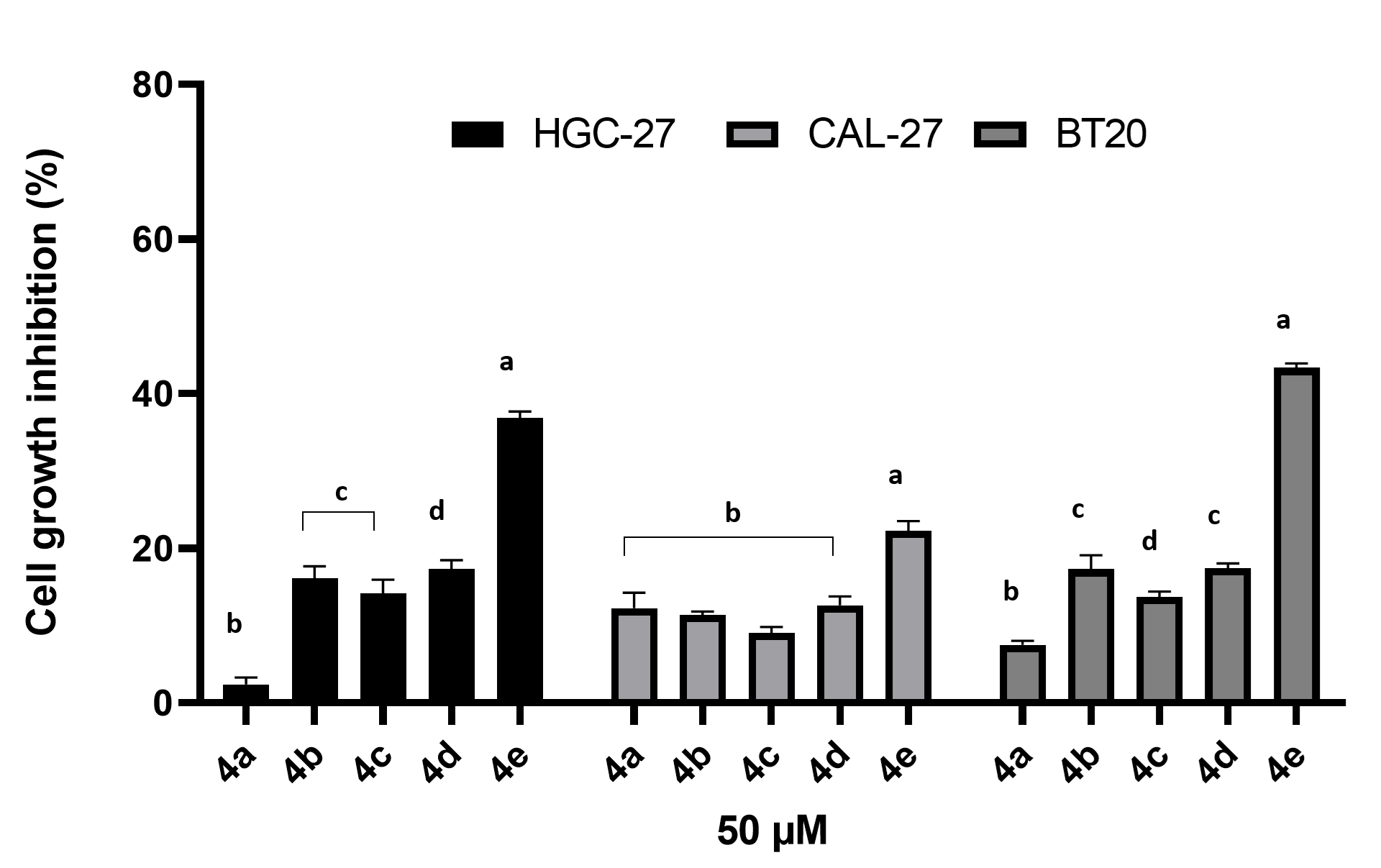


**Figure S1 -** Inhibition of cell growth (%) in the BT20, HGC27, and CAL-27 lines of the ketone intermediates **4a**-**4e**. The results were expressed as means ± standard error of the mean (SEM) and were analyzed using One Way Analysis of Variance (ANOVA). For all variables with the same letter, the difference between the means is not statistically significant.

1. **Inhibition values [%] of compounds 5f, 6a - 6e, 7a - 7e, 8a – 8k at P2X7 and P2X4 receptors**

| Cmpd Nr | % Inhibition of P2X7R at 10 µM compounds’ concentration | % Inhibition of P2X4R at 10 µM compounds’ concentration |
| --- | --- | --- |
| 5f | 5 | 4 |
| 6a | -- | -- |
| 6b | 10 | -3 |
| 6c | -8 | 3 |
| 6d | 5 | 0 |
| 6e | 7 | 9 |
| 7a | -6 | -7 |
| 7b | 4 | 1 |
| 7c | -9 | -2 |
| 7d | 2 | 8 |
| 7e | 4 | -3 |
| 8a | 9 | 5 |
| 8b | -7 | -6 |
| 8c | 3 | 10 |
| 8d | -10 | 2 |
| 8e | 8 | -9 |
| 8f | 5 | 4 |
| 8g | 1 | -8 |
| 8h | -1 | 6 |
| 8i | 6 | -1 |
| 8j | -2 | 0 |
| 8k | 10 | 7 |
| AZD9056 | pIC_50_=7.98±0.16 | -- |
| Bay1797 | -- | pIC_50_=7.02±0.07 |

AZD9056 is a highly potent and selective P2X7R antagonist. Bay1797 is a potent and selective P2X4R antagonist. AZD9056 and Bay1797 were tested under the same conditions as reference compounds.

1. **NMR, GC-MS, HRAM-MS data.**


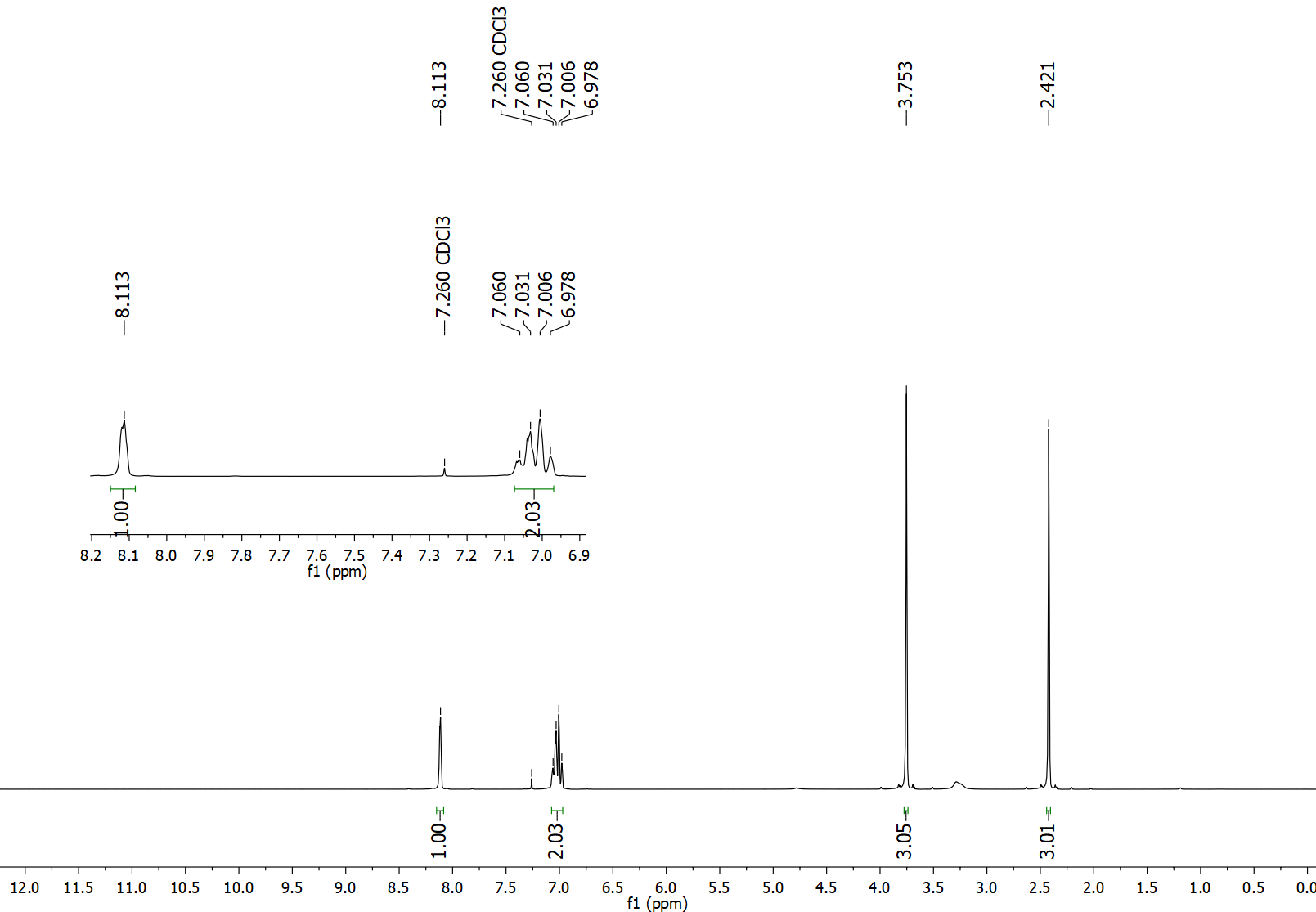


**^1^H NMR (300MHz, CDCl_3_) 5-methoxy-2-methyl-pyridine**


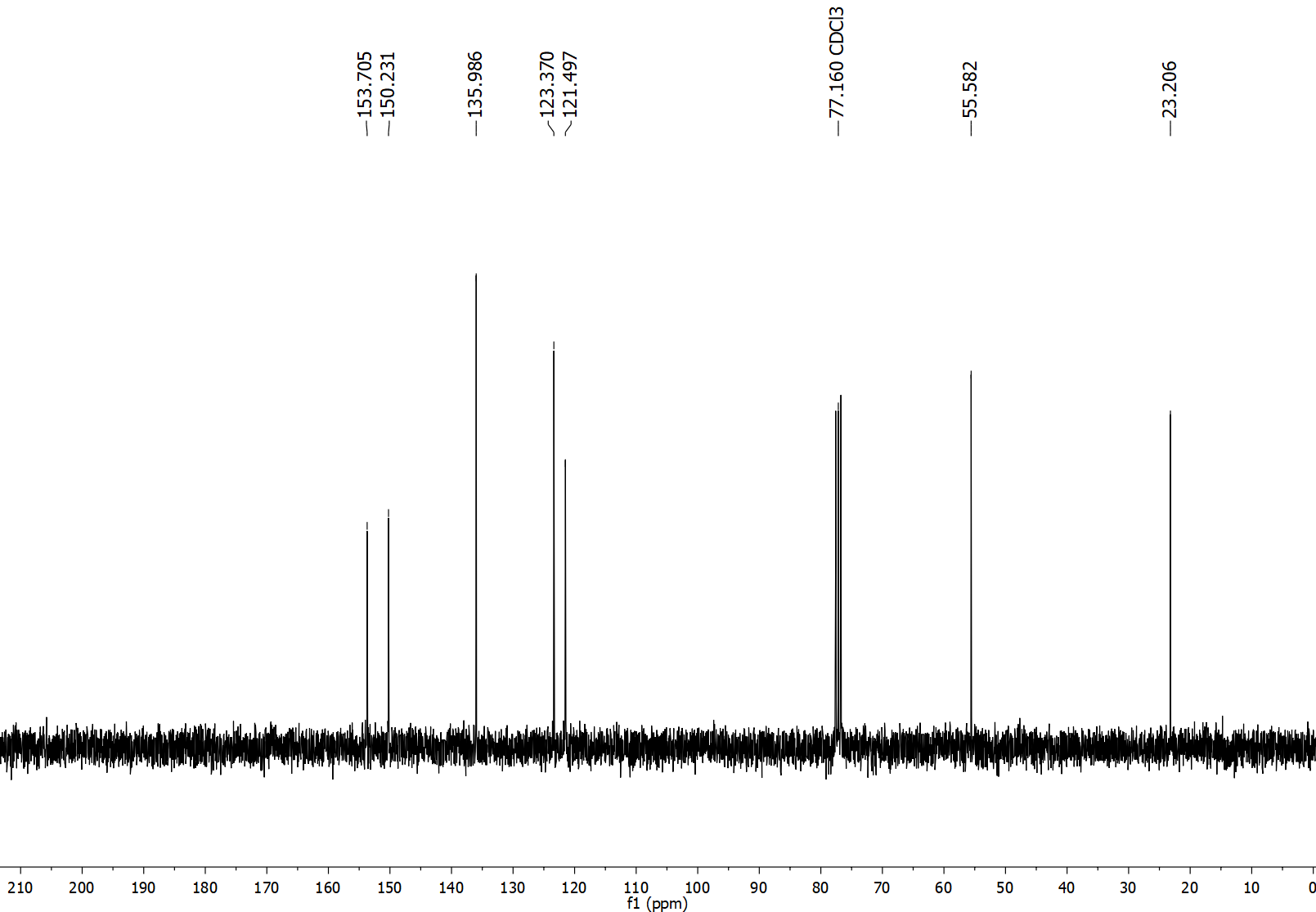


**^13^C NMR (75MHz, CDCl_3_) 5-methoxy-2-methyl-pyridine**


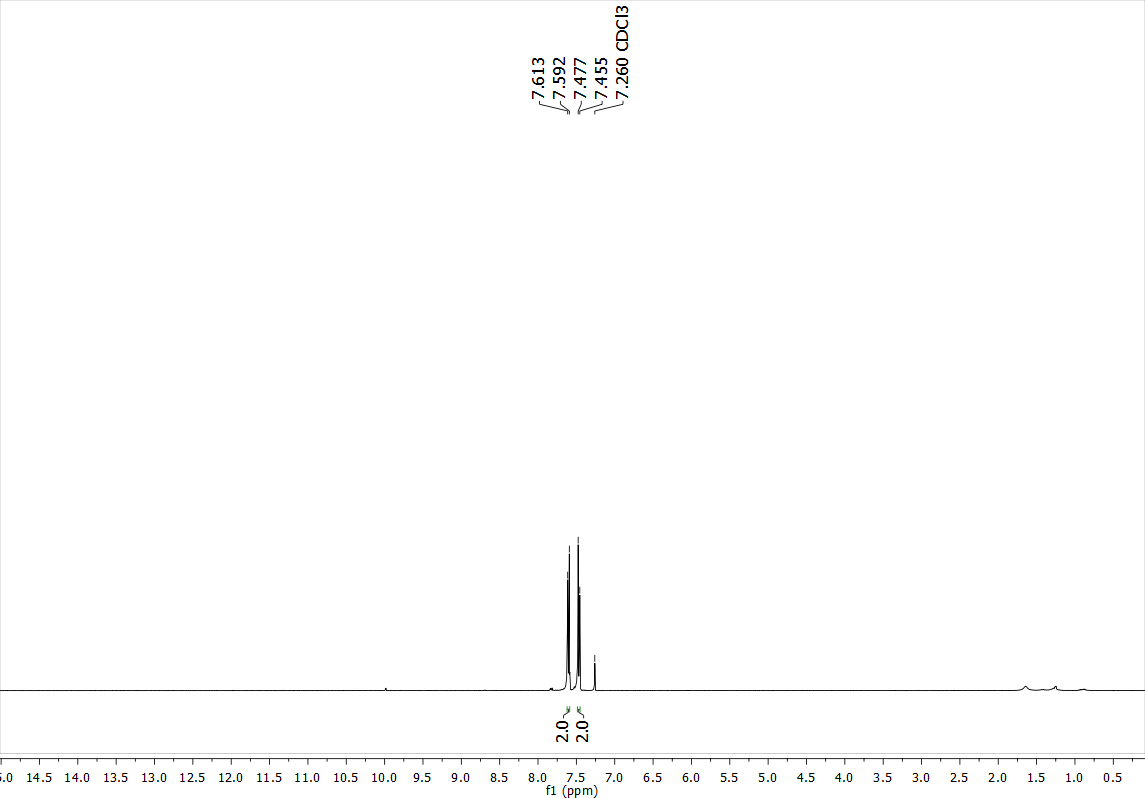


**^1^H NMR (400MHz, CDCl_3_) Benzonitrile 2b**

**
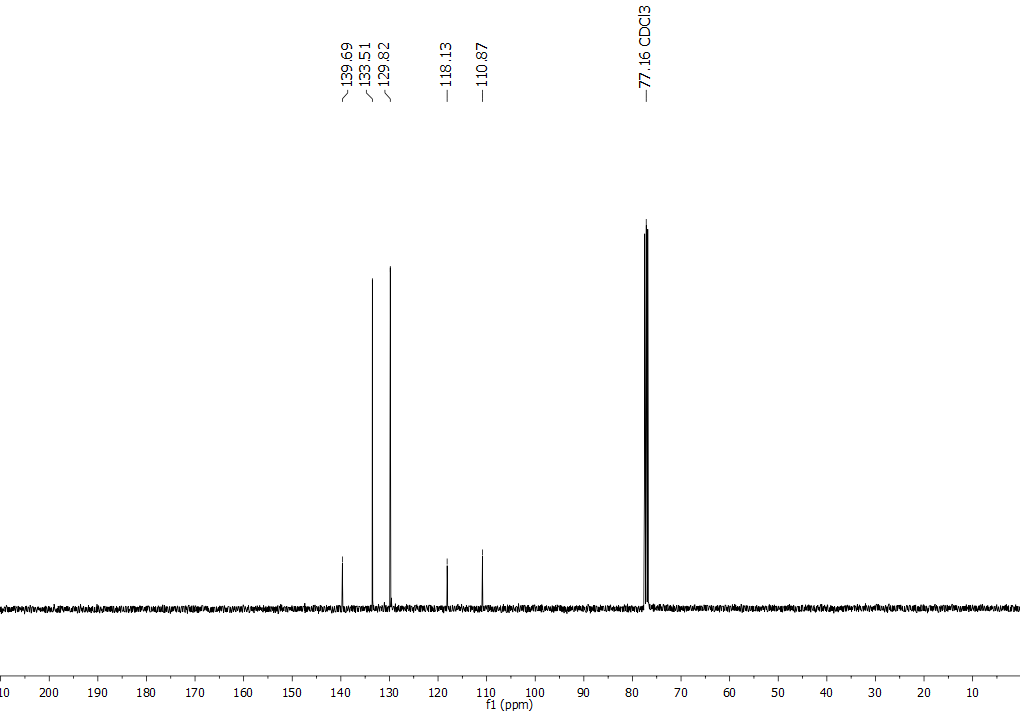
**

**^13^C NMR (100MHz, CDCl_3_) Benzonitrile 2b**


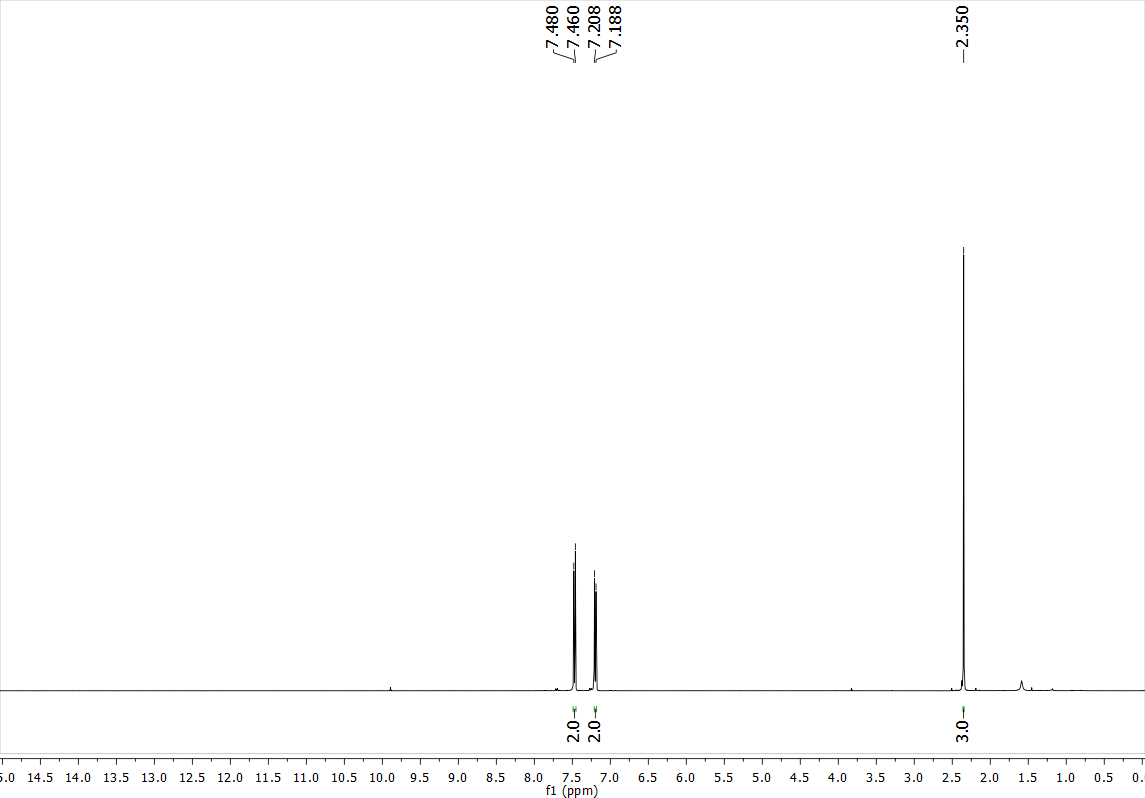


**^1^H NMR (400MHz, CDCl_3_) Benzonitrile 2c**

**
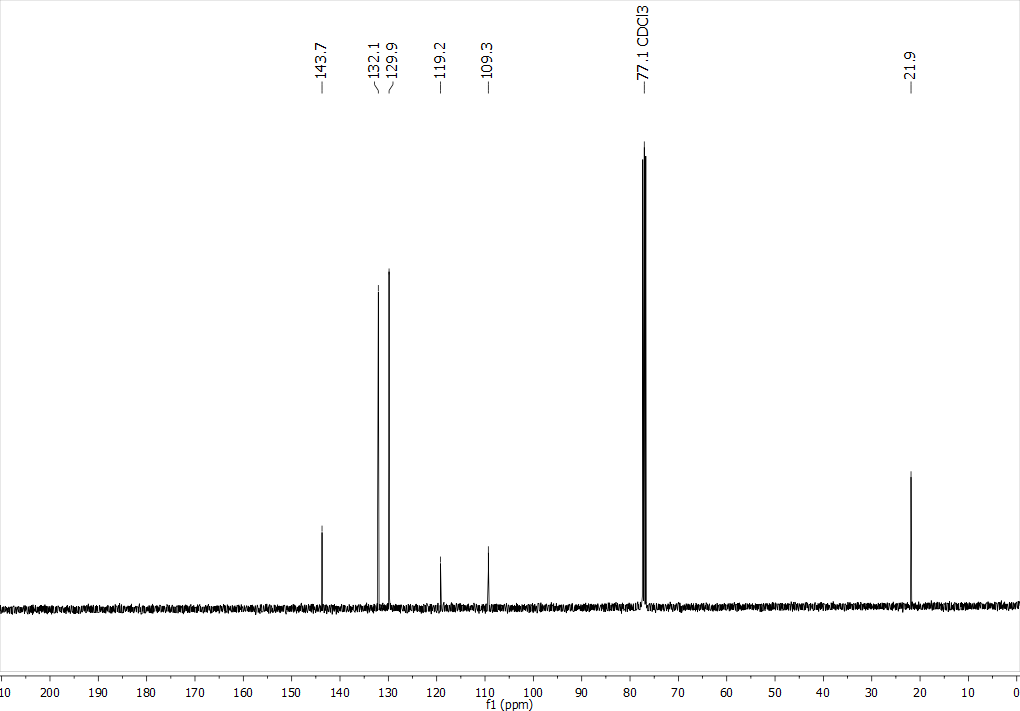
**

**^13^C NMR (100MHz, CDCl_3_) Benzonitrile 2c**

**^^**
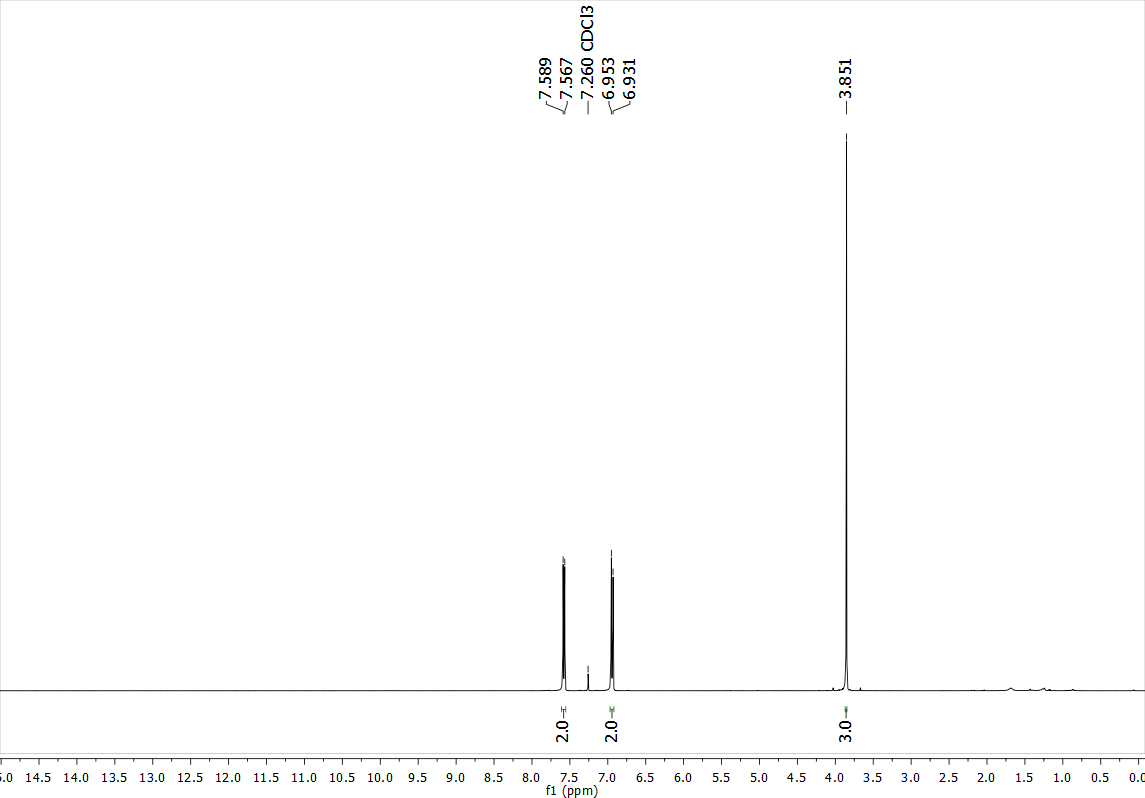


**^1^H NMR (400MHz, CDCl_3_) Benzonitrile 2d**

**^^
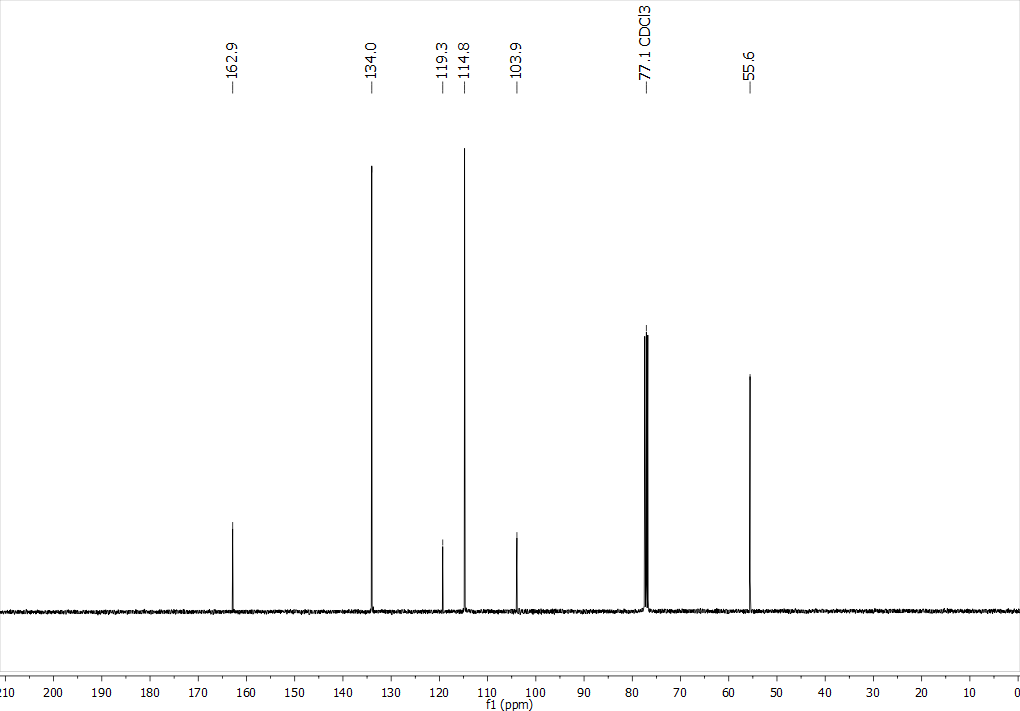
**

**^13^C NMR (100MHz, CDCl_3_) Benzonitrile 2d**


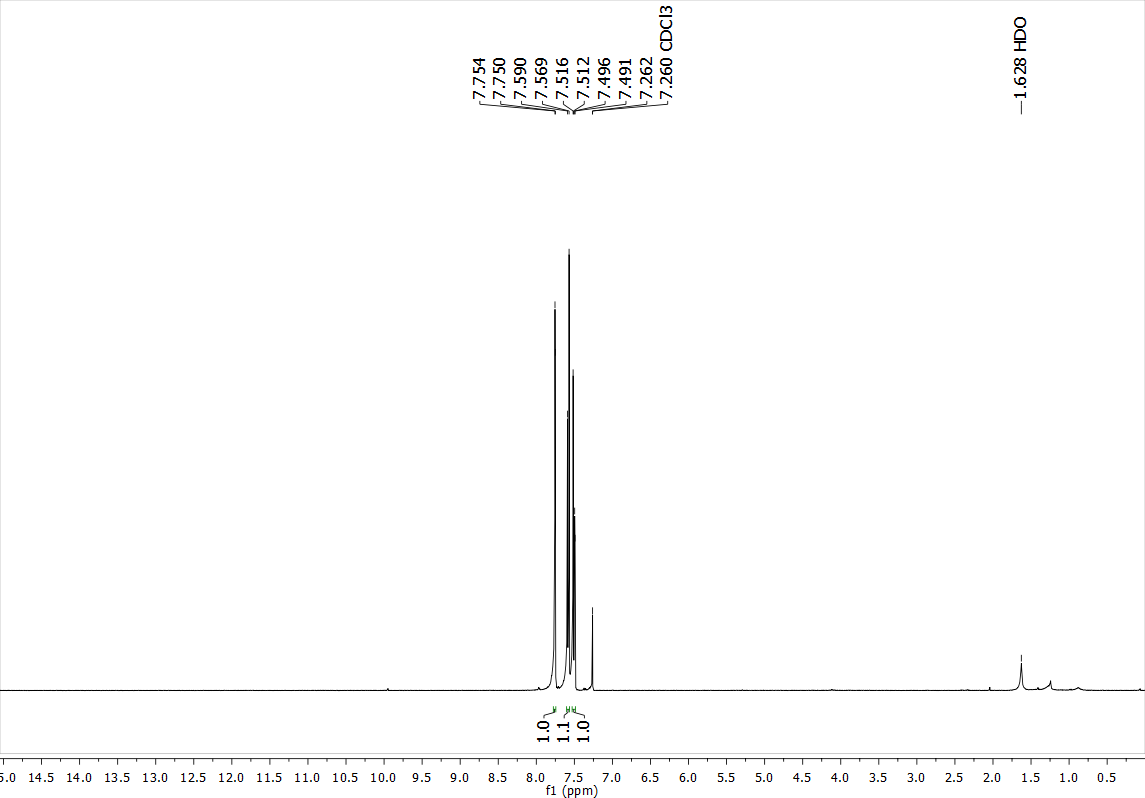


**^1^H NMR (400MHz, CDCl_3_) Benzonitrile 2e**

**^
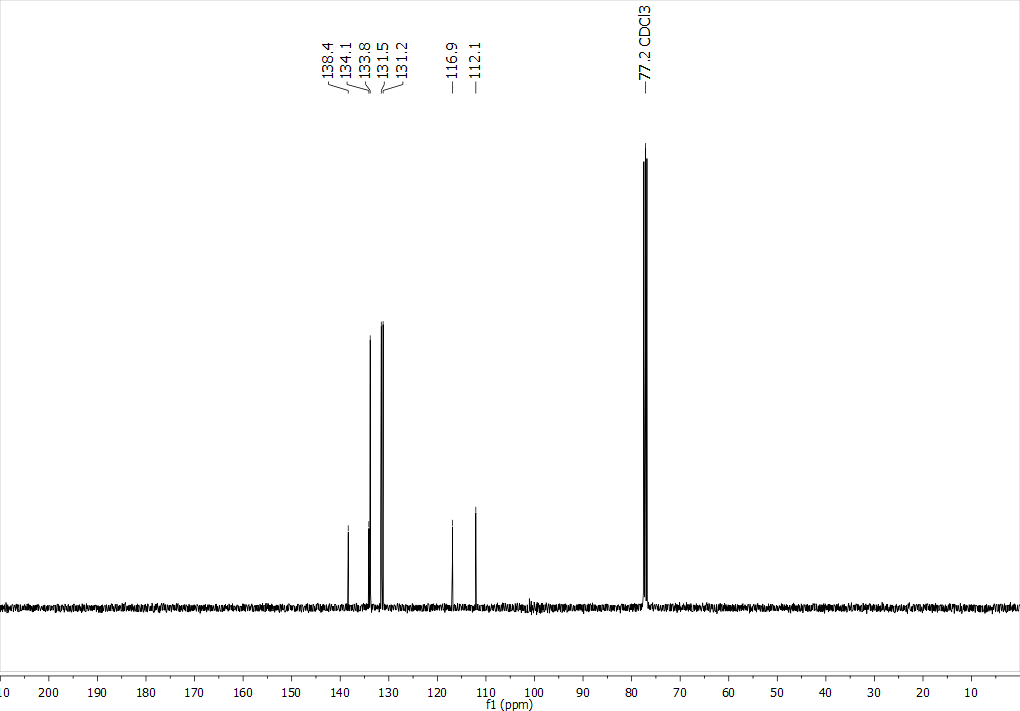
^**

**^13^C NMR (100MHz, CDCl_3_) Benzonitrile 2e**


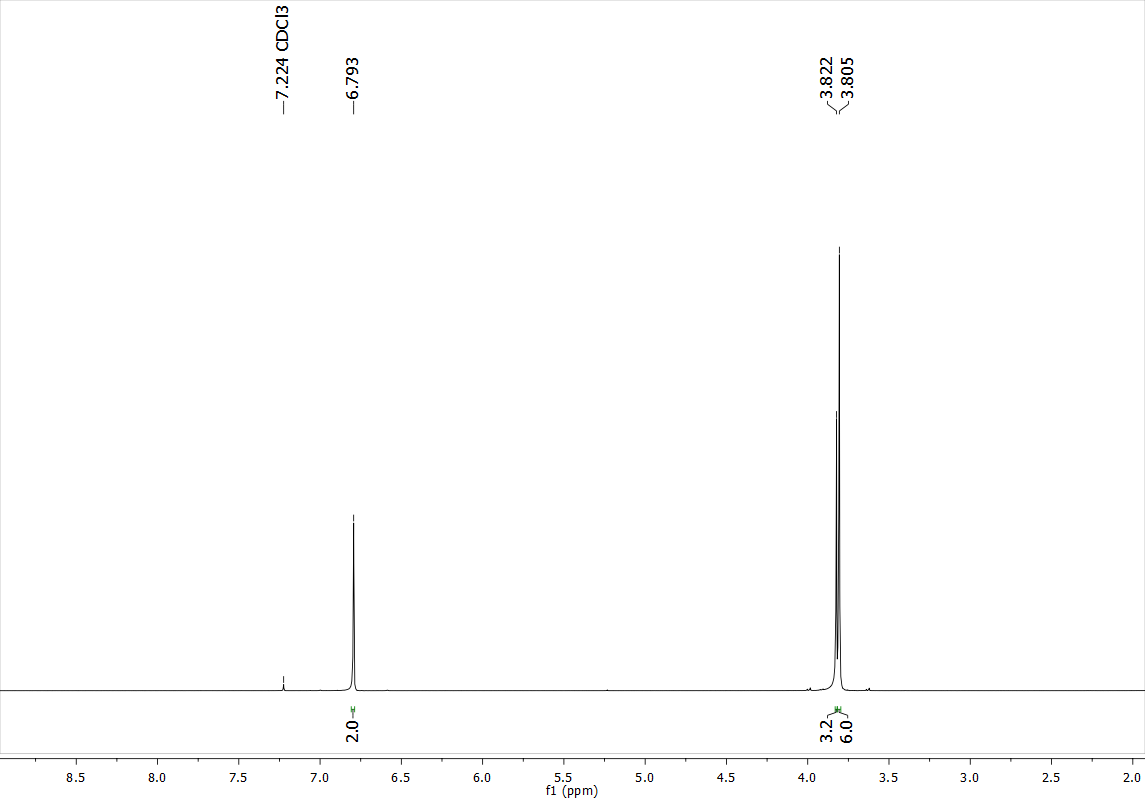


**^1^H NMR (400MHz, CDCl_3_) Benzonitrile 2f**

**
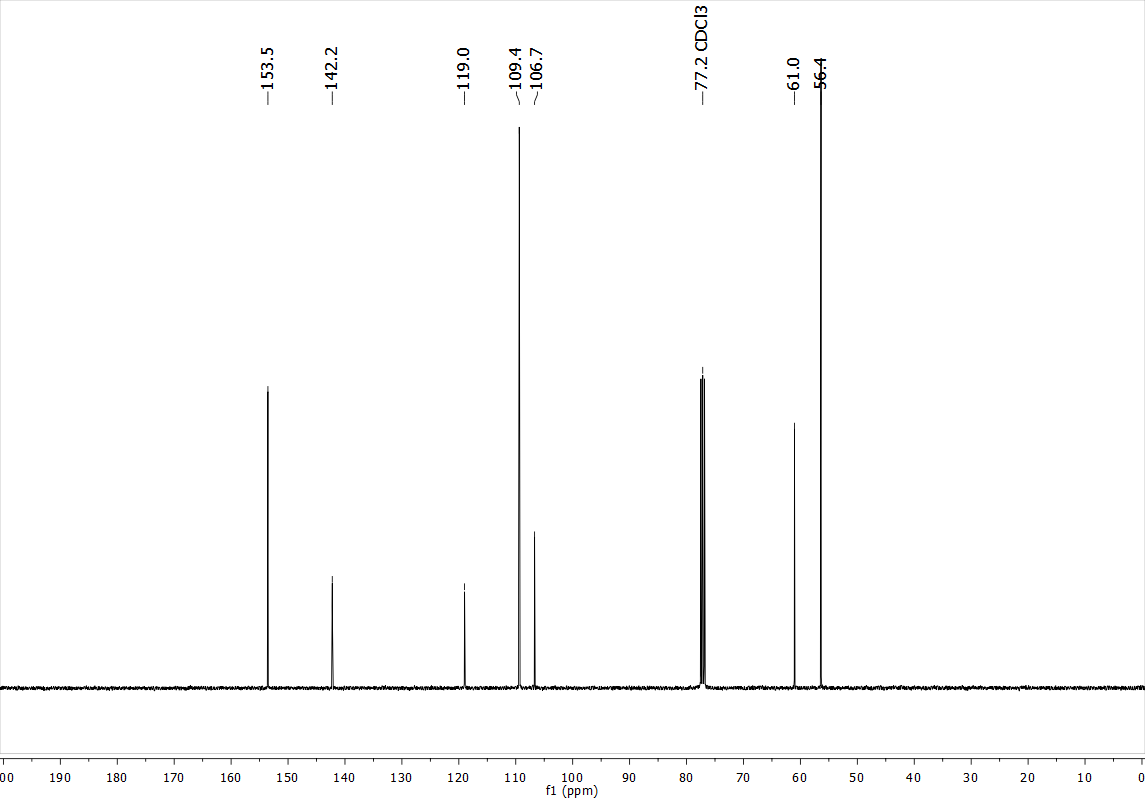
**

**^13^C NMR (100MHz, CDCl_3_) Benzonitrile 2f**


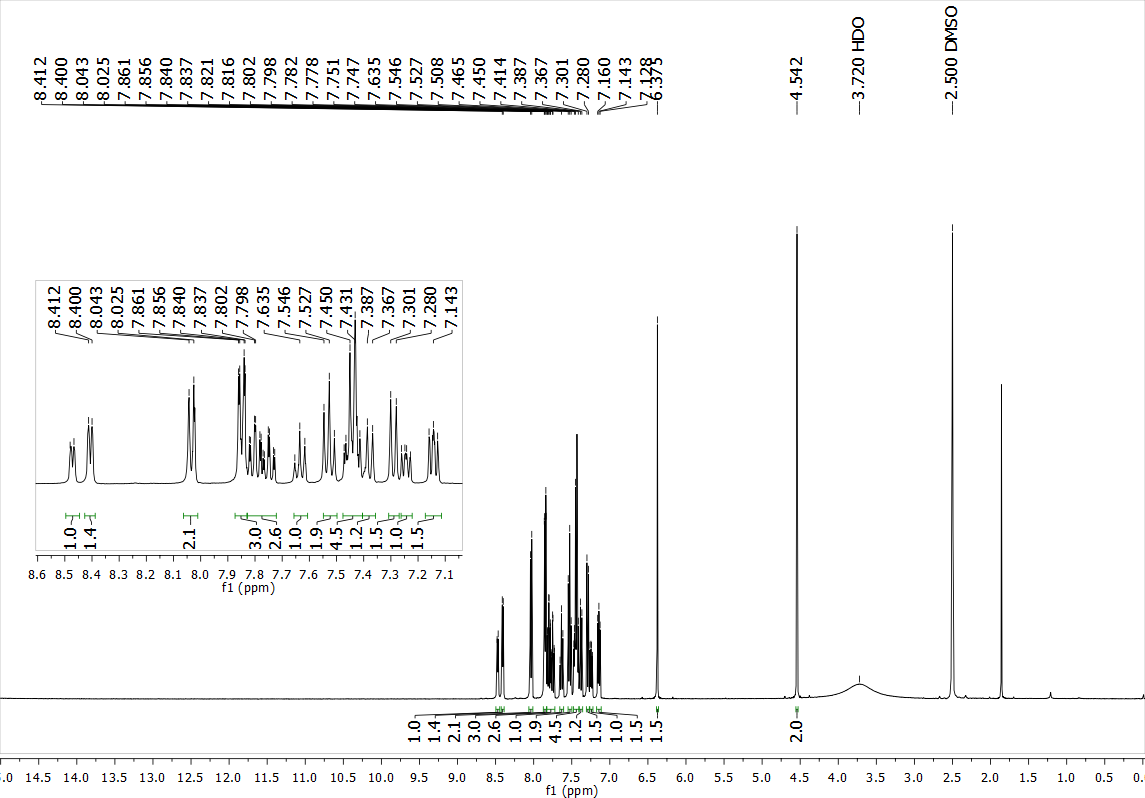


**^1^H NMR (400MHz, DMSO-d_6_) 4a**


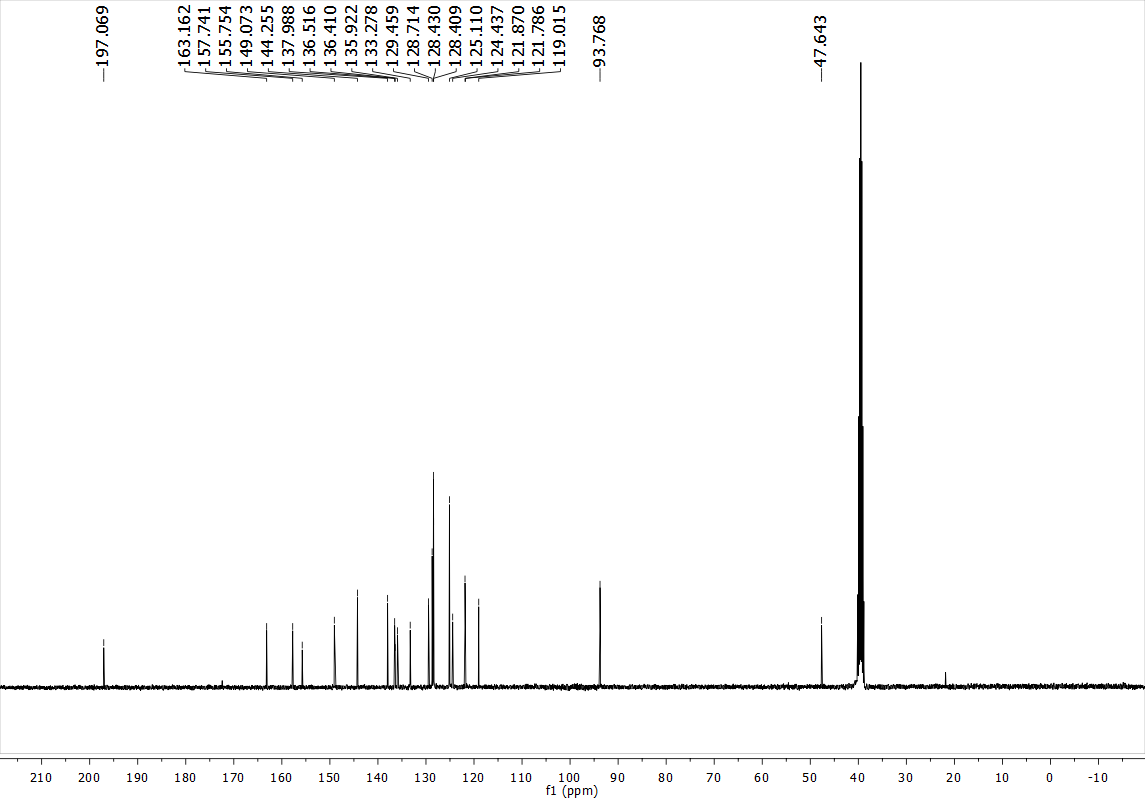


**^13^C NMR (100MHz, DMSO-d_6_) 4a**

**HPLC Purity - 4a (Equipment 2, Method B) R_t_: 15.3 min**


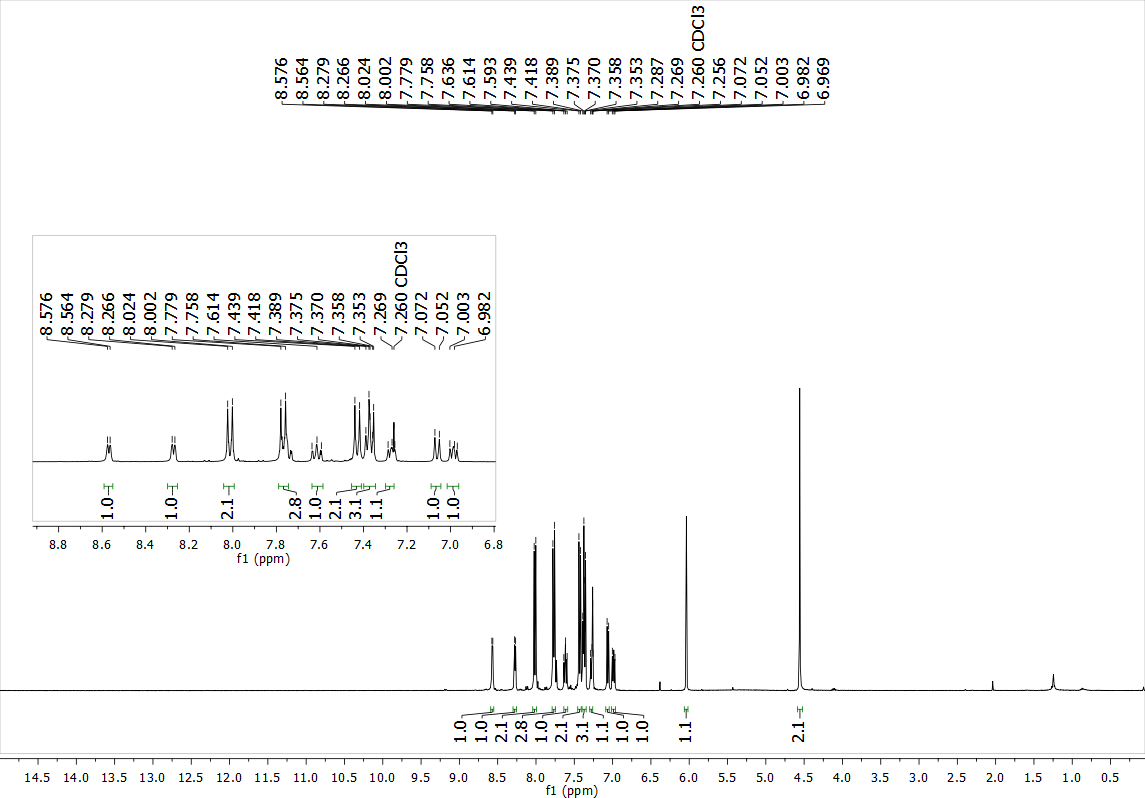


**^1^H NMR (400MHz, DMSO-d_6_) 4b**

**
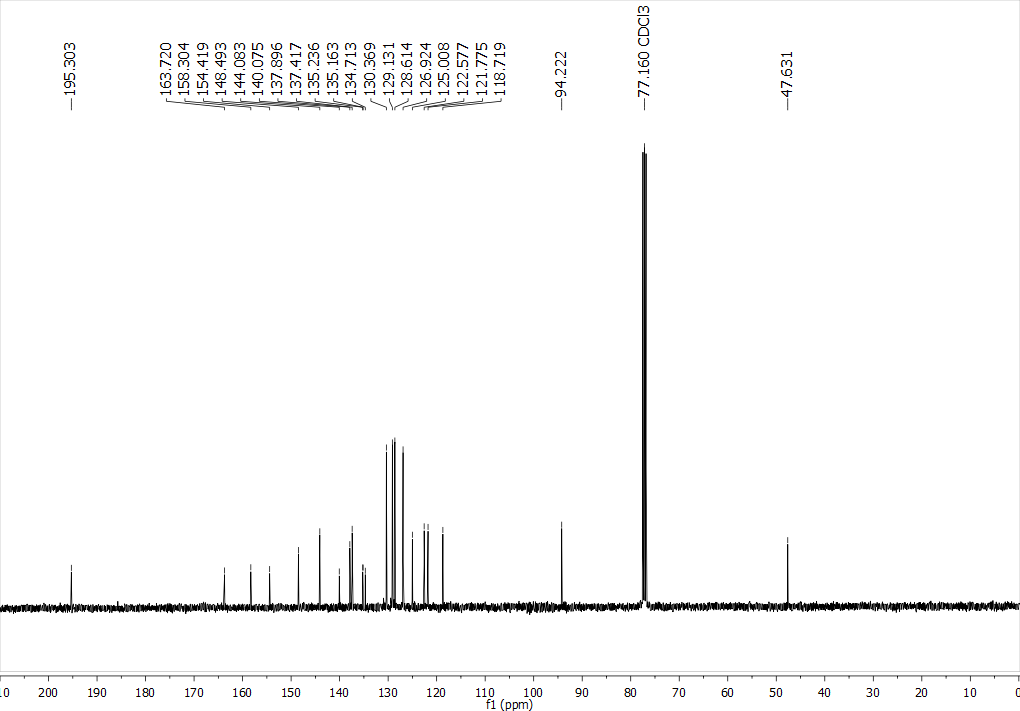
**

**^13^C NMR (100MHz, DMSO-d_6_) 4b**

**
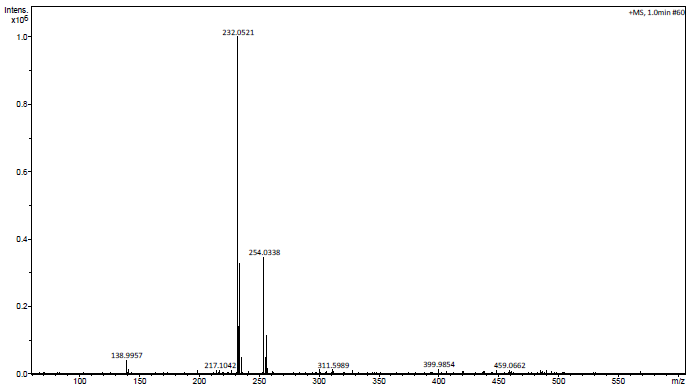
**

**HRAM-MS**  **(ESI+) 4b**

**HPLC Purity - 4b (Equipment 2, Method C) R_t_: 28.5 min**


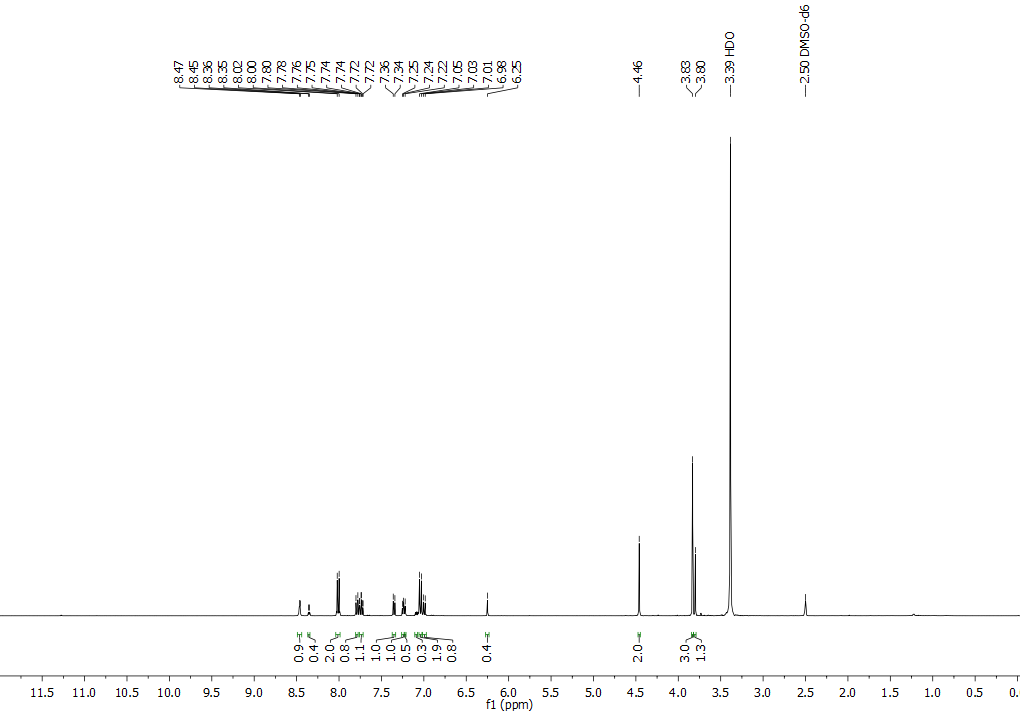


**^1^H NMR (400MHz, DMSO-d_6_) 4c**

**
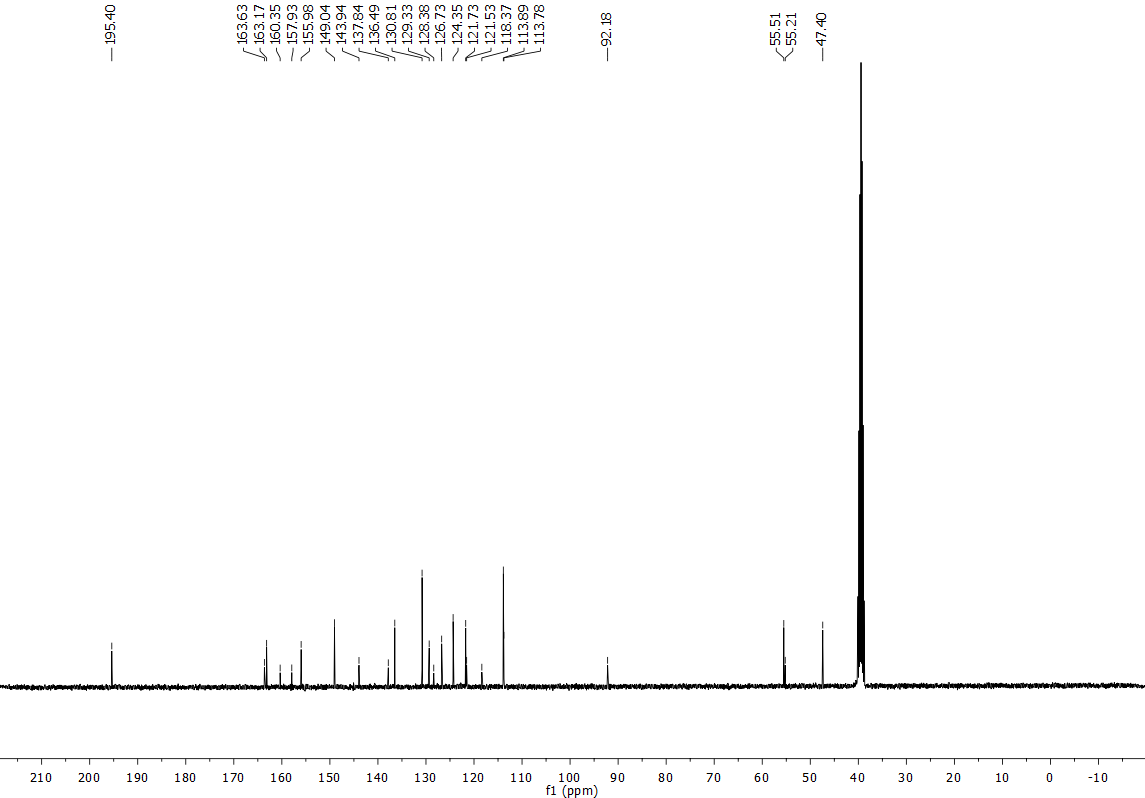
**

**^13^C NMR (100MHz, DMSO-d_6_) 4c**

**
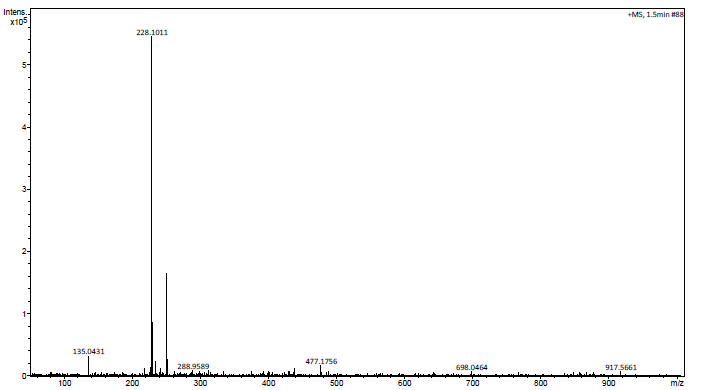
**

**HRAM-MS**  **(ESI+) 4c**

**HPLC Purity - 4c (Equipment 2, Method B) R_t_: 8.9 min**


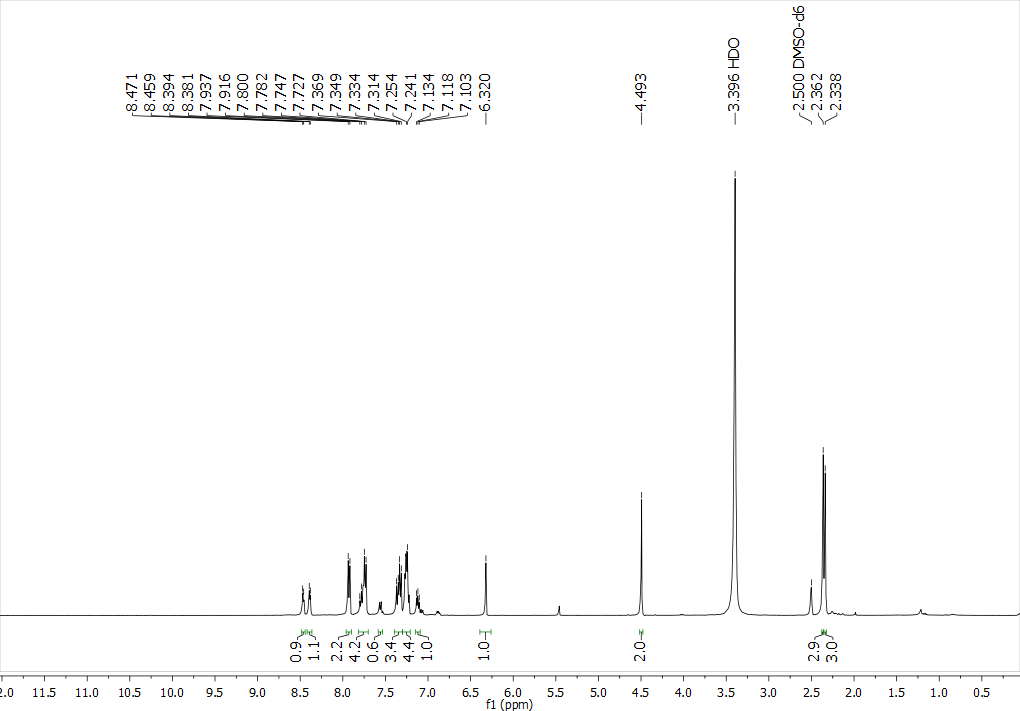


**^1^H NMR (400MHz, DMSO-d_6_) 4d**


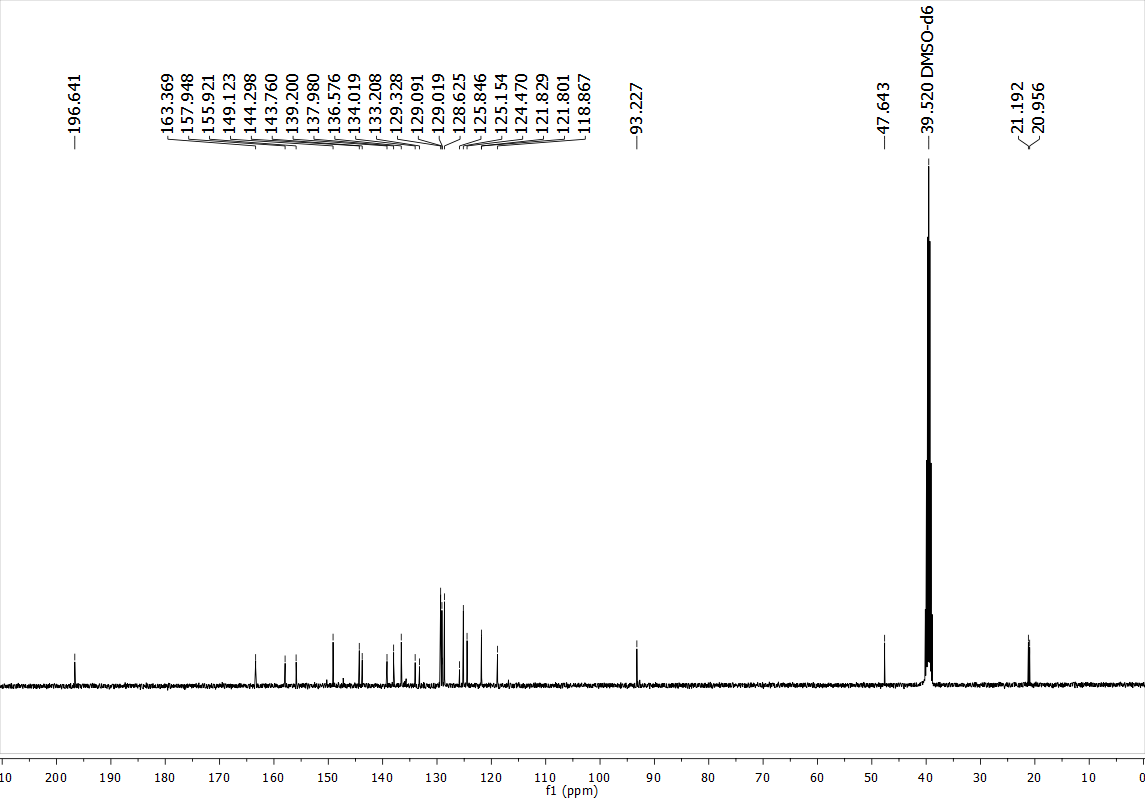


**^13^C NMR (100MHz, DMSO-d_6_) 4d**

**
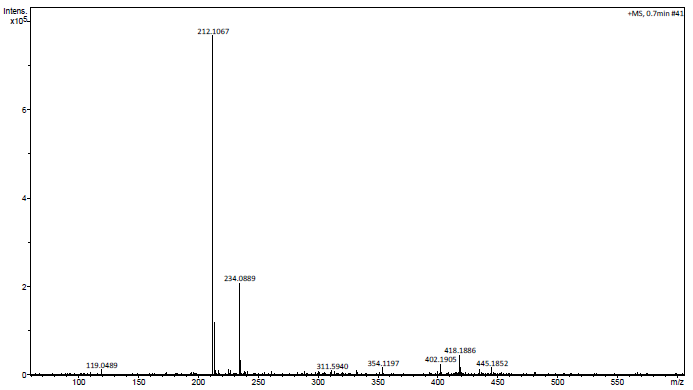
**

**HRAM-MS**  **(ESI+) 4d**

**HPLC Purity - 4d (Equipment 2, Method C) R_t_: 18.2 min**


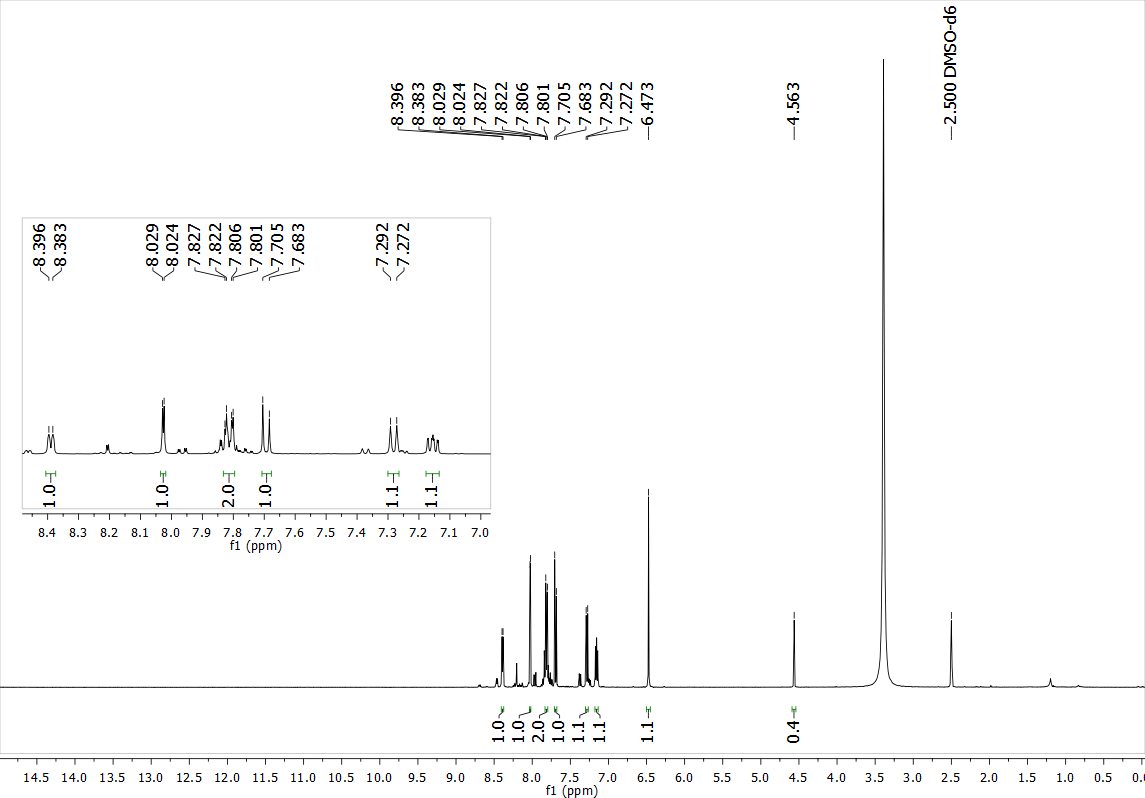


**^1^H NMR (400MHz, DMSO-d_6_) 4e**


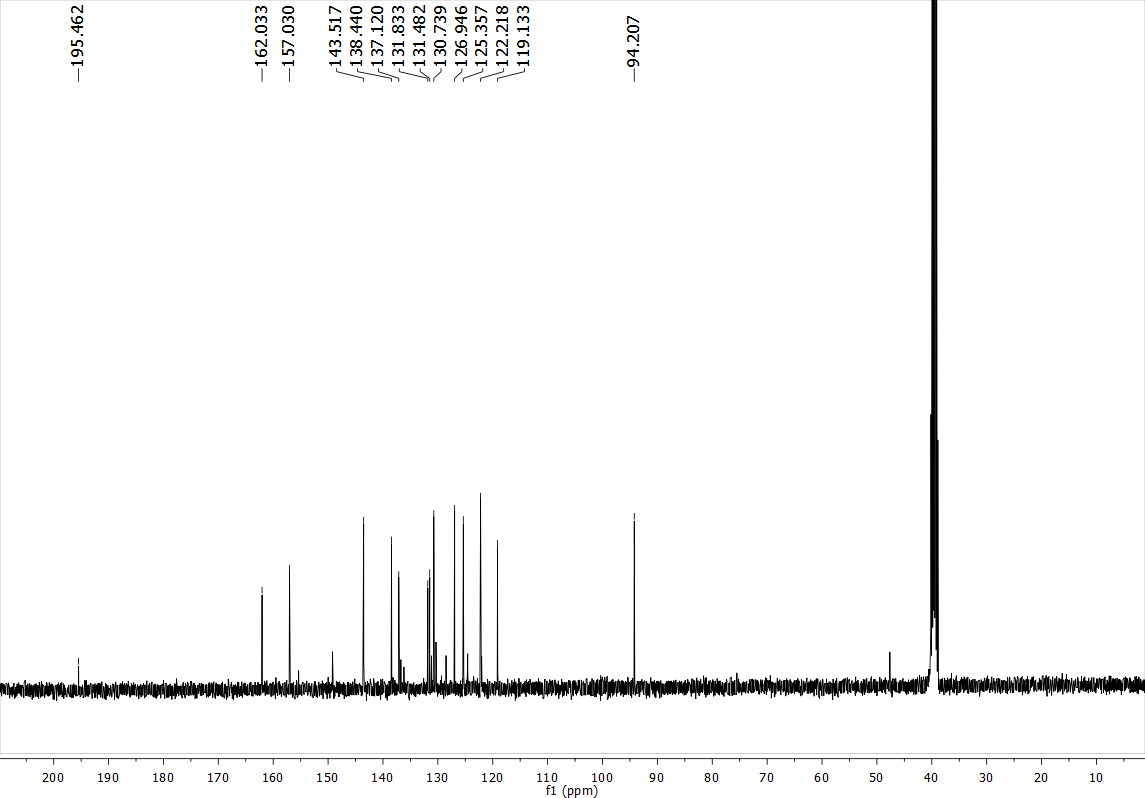


**^13^C NMR (100MHz, DMSO-d_6_) 4e**

**
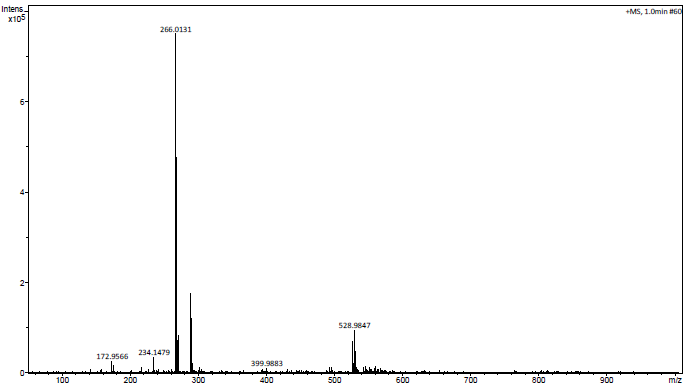
**

**HRAM-MS**  **(ESI+) 4e**

**HPLC Purity - 4e (Equipment 2, Method B) R_t_: 15.7 min**

**
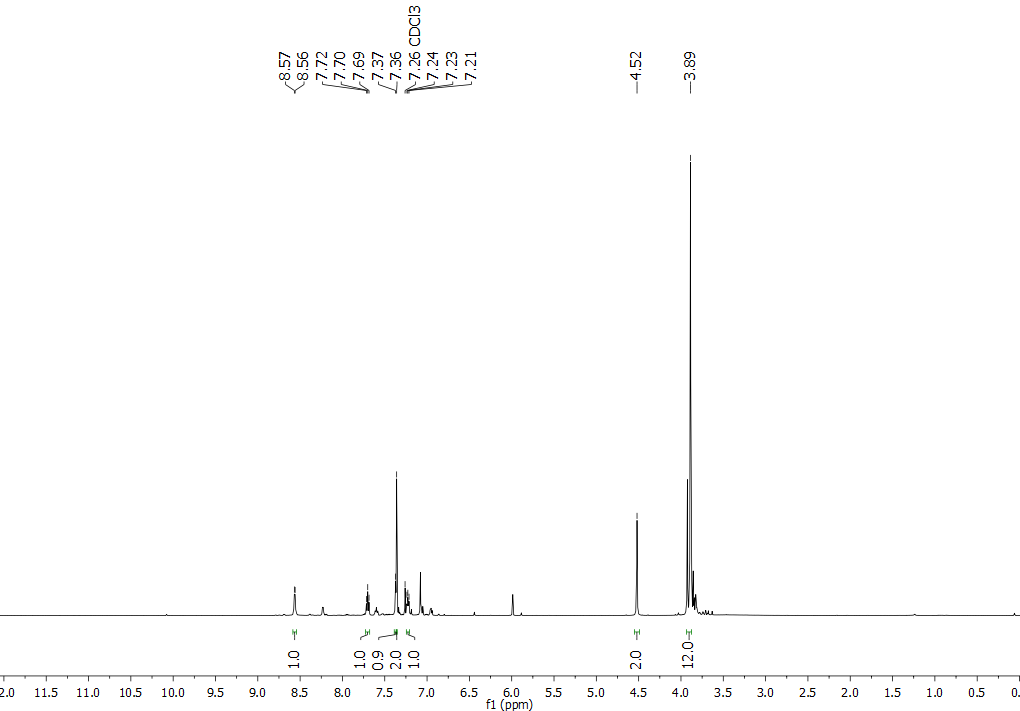
**

**^1^H NMR (500MHz, CDCl_3_) 4f**

**
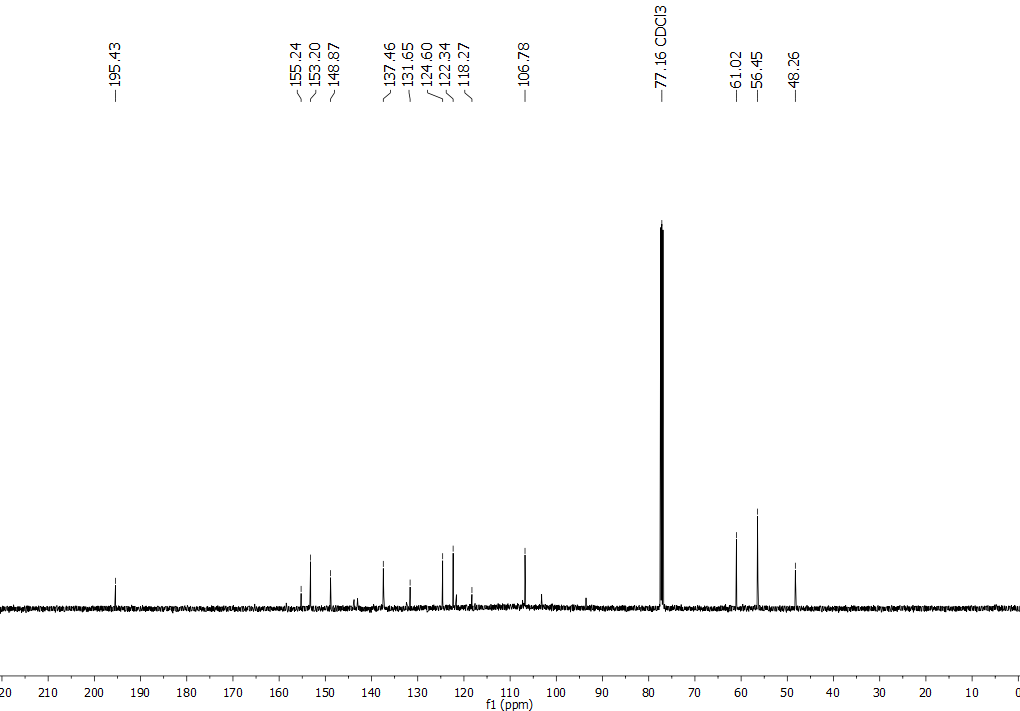
**

**^13^C NMR (125MHz, CDCl_3_) 4f**

**
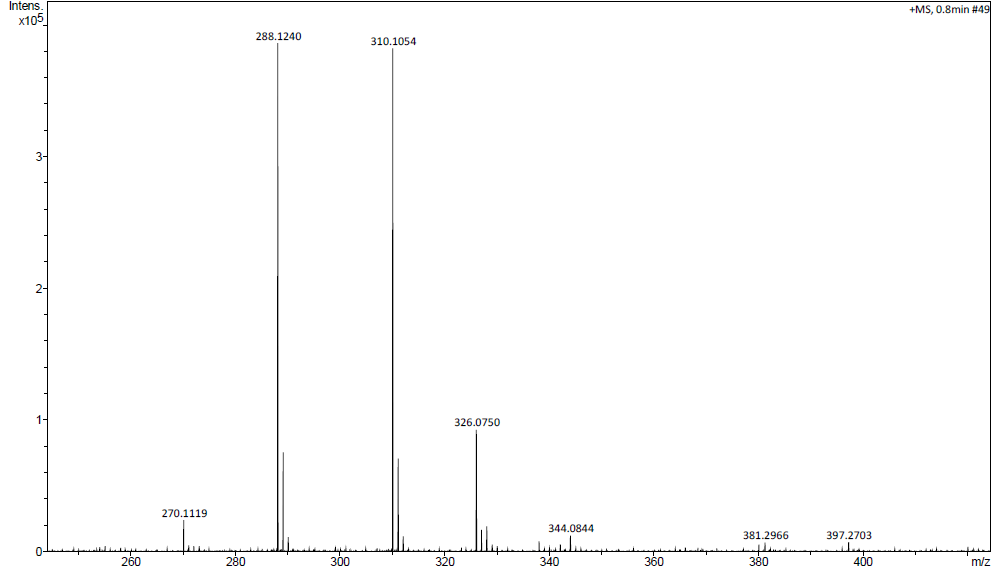
**

**HRAM-MS**  **(ESI+) 4f**

**HPLC Purity - 4f (Equipment 2, Method B) R_t_: 15.7 min**


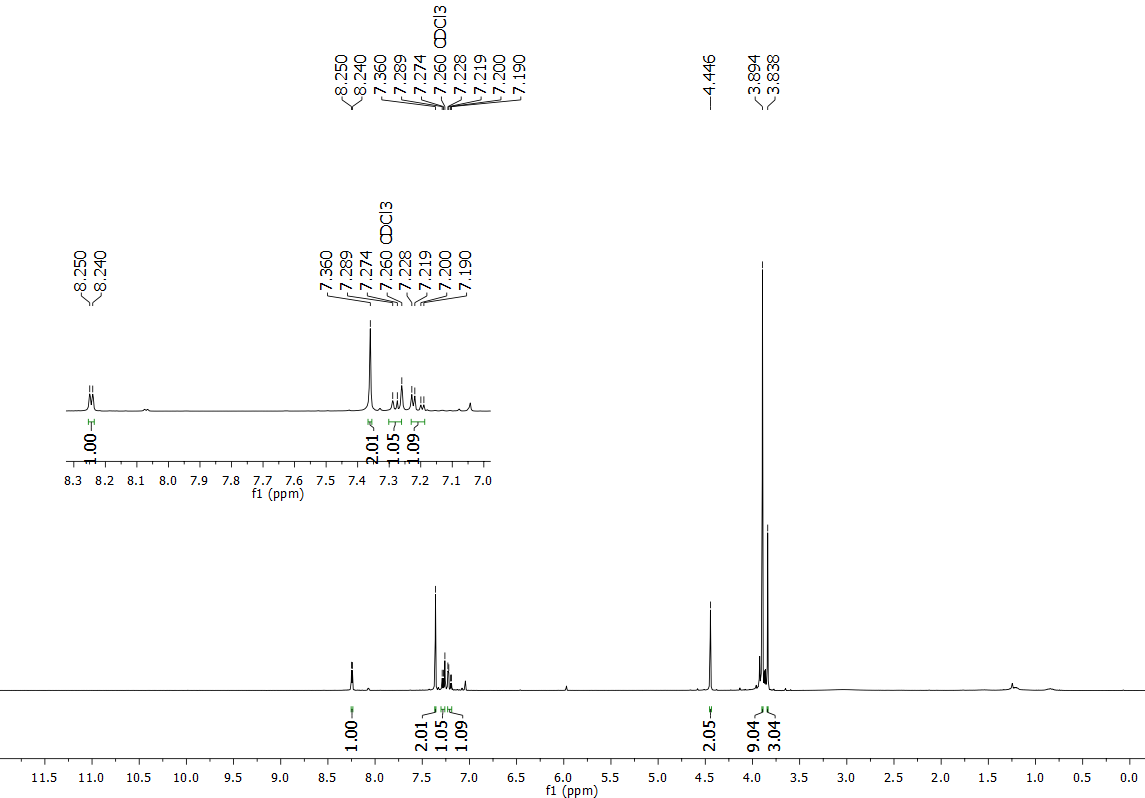


**^1^H NMR (300MHz, CDCl_3_) 5f**


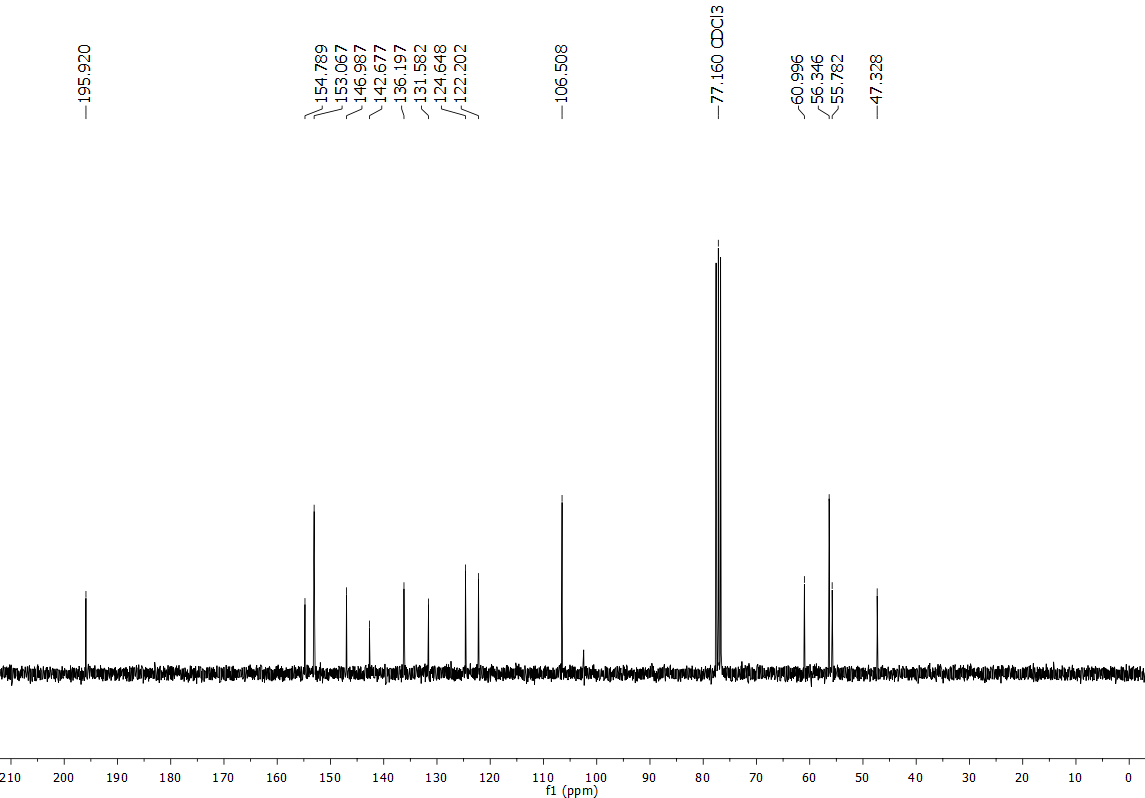


**^13^C NMR (75MHz, CDCl_3_) 5f**

**
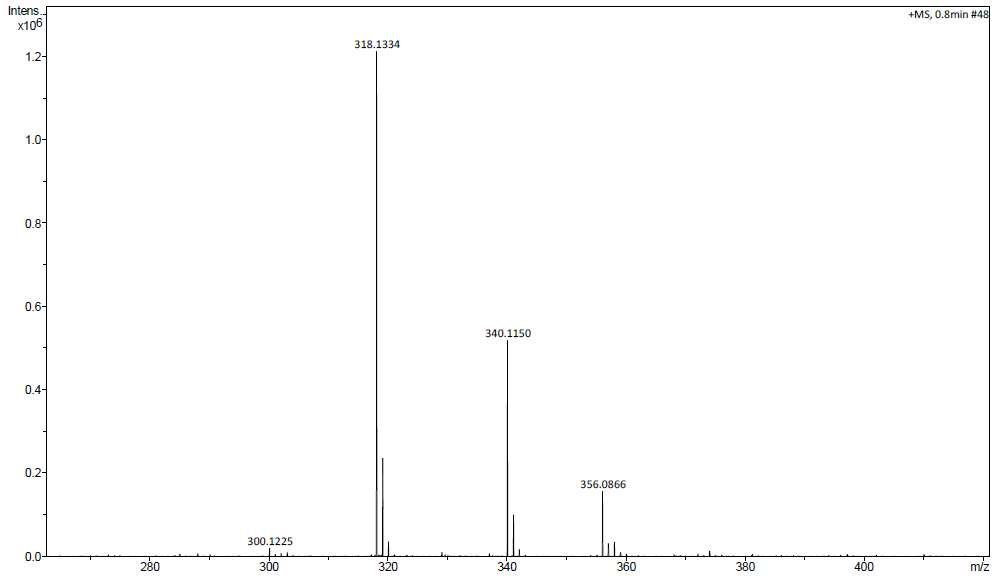
**

**HRAM-MS**  **(ESI+) 5f**

**
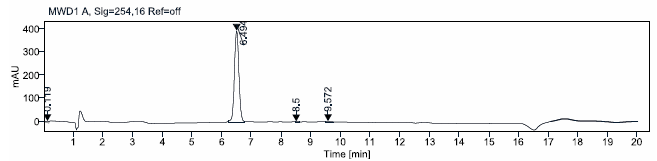

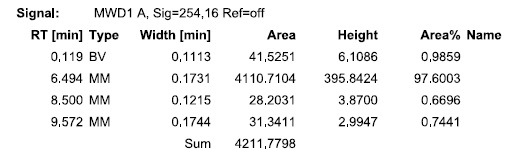
**

**HPLC Purity - 5f (Equipment 1, Method A) R_t_: 6.49 min**

**
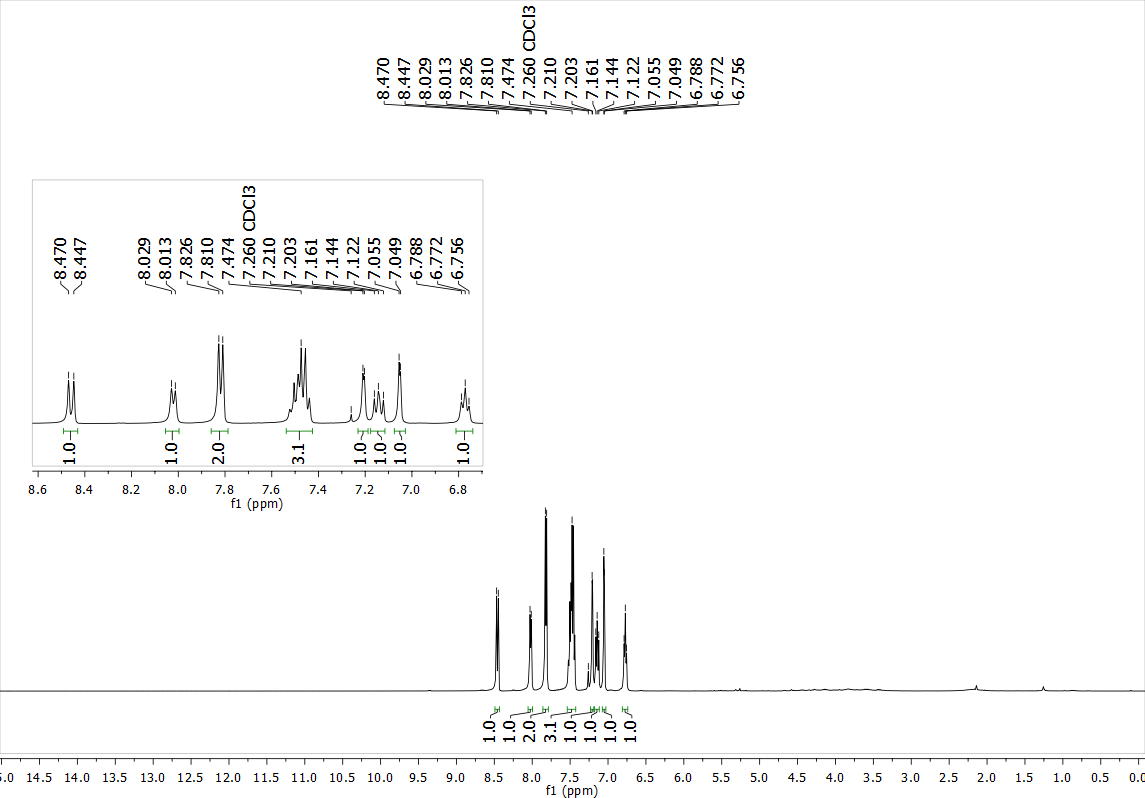
**

**^1^H NMR (400MHz, CDCl_3_) 6a**

**
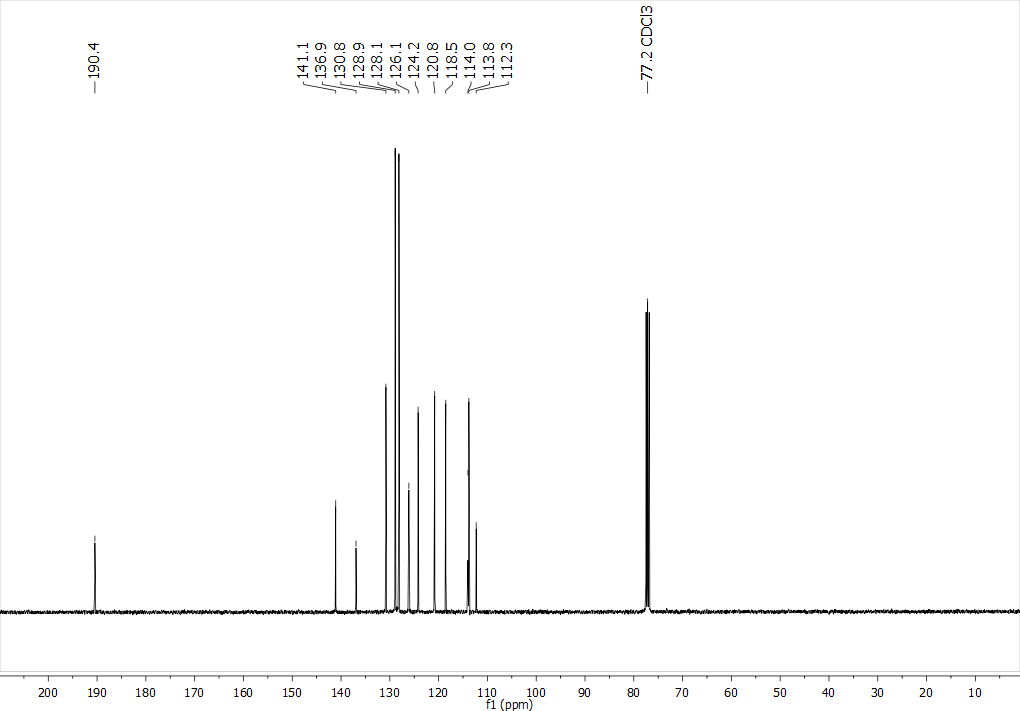
**

**^13^C NMR (100MHz, CDCl_3_) 6a**

**
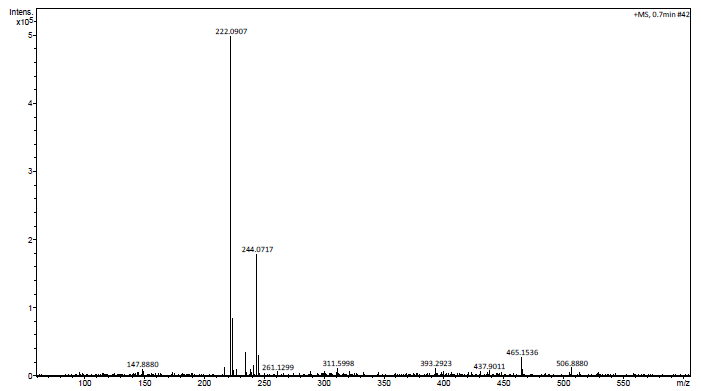
**

**HRAM-MS**  **(ESI+) 6a**

**HPLC Purity - 6a (Equipment 2, Method B) R_t_: 14.2 min**

**^
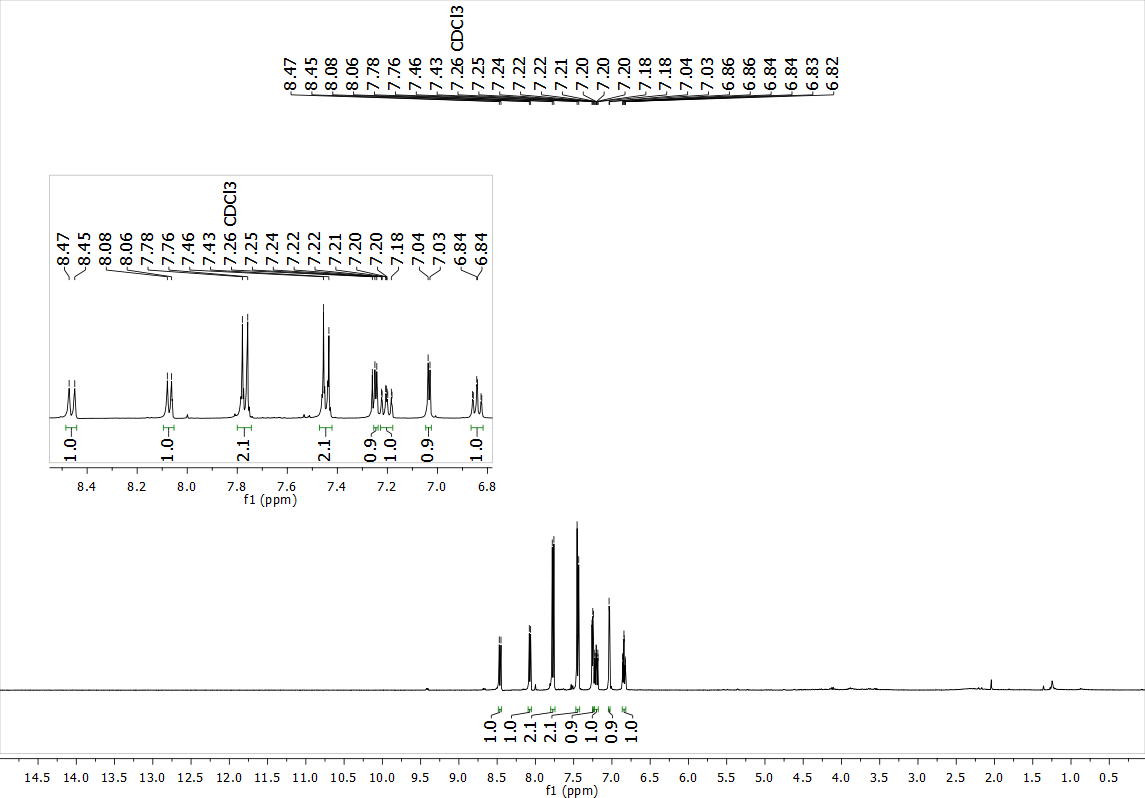
^**

**^1^H NMR (400MHz, CDCl_3_) 6b**

**
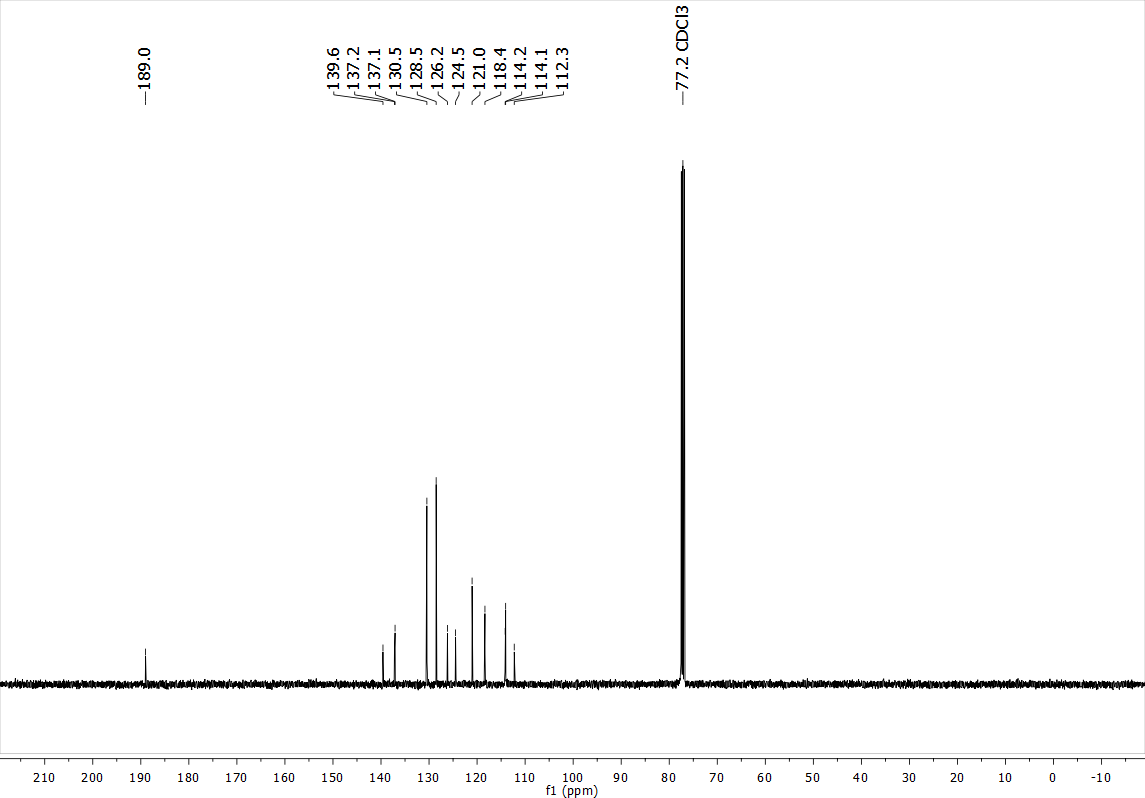
**

**^13^C NMR (100MHz, CDCl_3_) 6b**

**
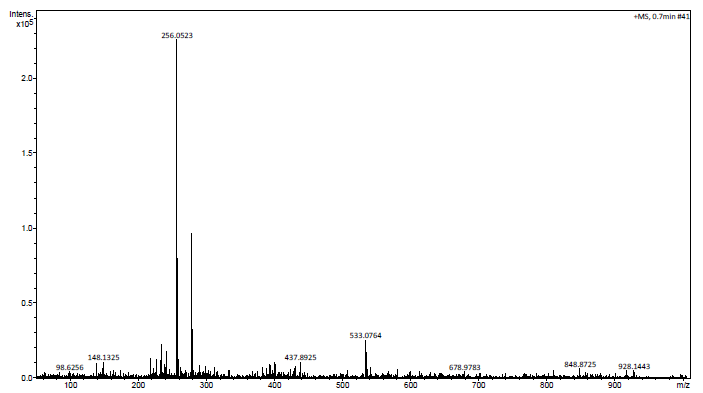
**

**HRAM-MS**  **(ESI+) 6b**

**
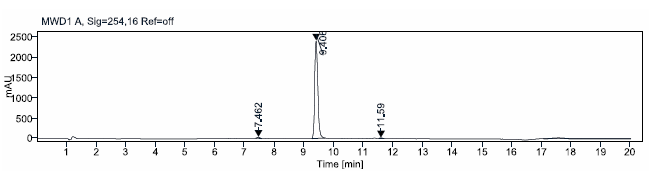
**

**
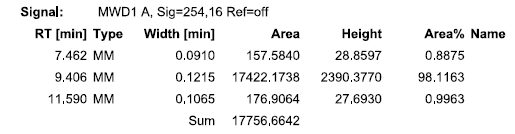
**

**HPLC Purity – 6b (Equipment 1, Method A) R_t_: 9.40 min**

**^
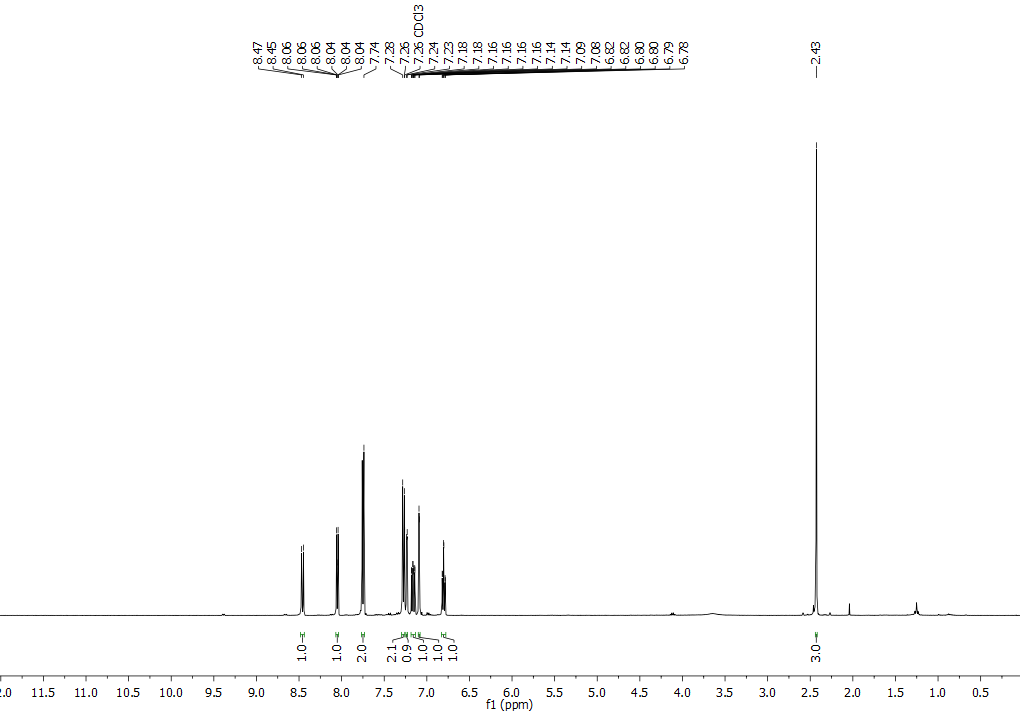
^**

**^1^H NMR (400MHz, CDCl_3_) 6c**

**
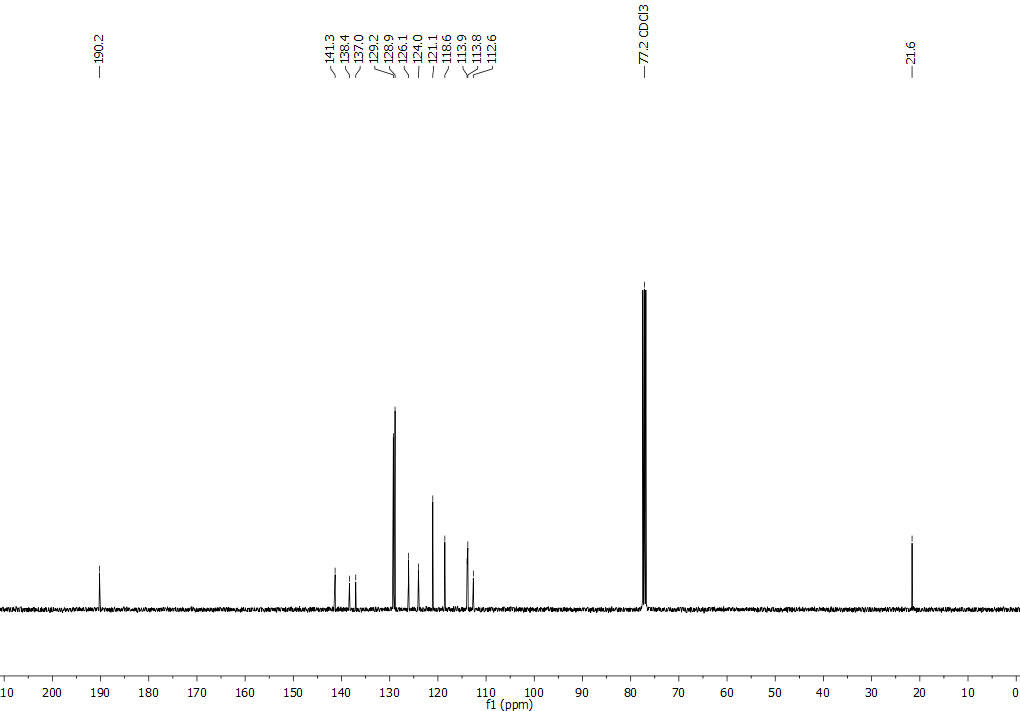
**

**^13^C NMR (100MHz, CDCl_3_) 6c**

**
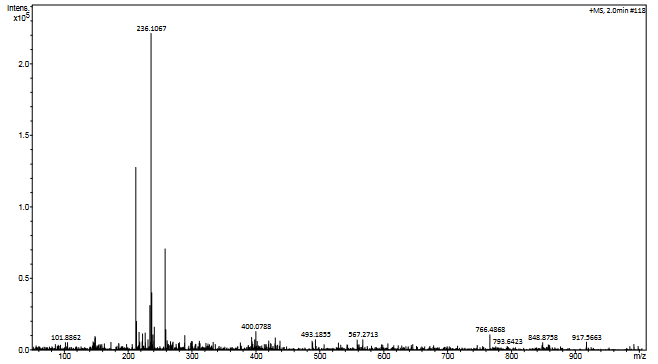
**

**HRAM-MS**  **(ESI+) 6c**

**
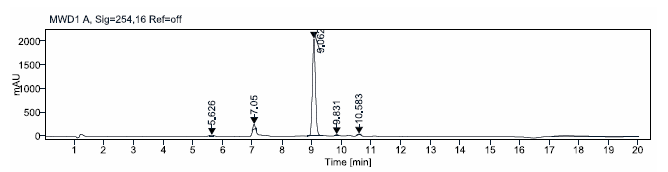
**

**
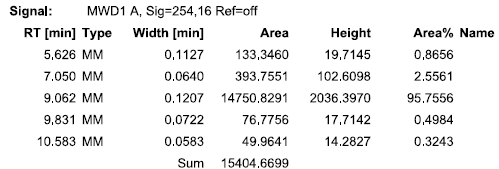
**

**HPLC Purity – 6c (Equipment 1, Method A) R_t_: 9.06 min**

**^
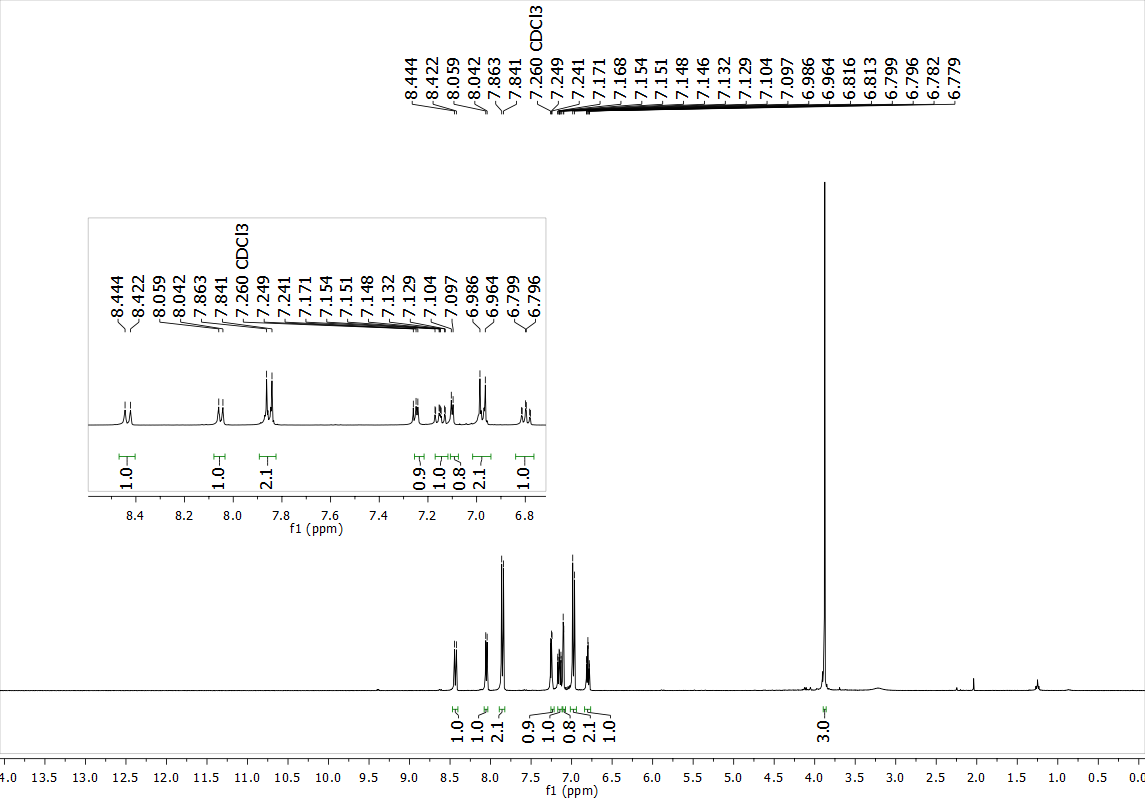
^**

**^1^H NMR (400MHz, CDCl_3_) 6d**

**^^
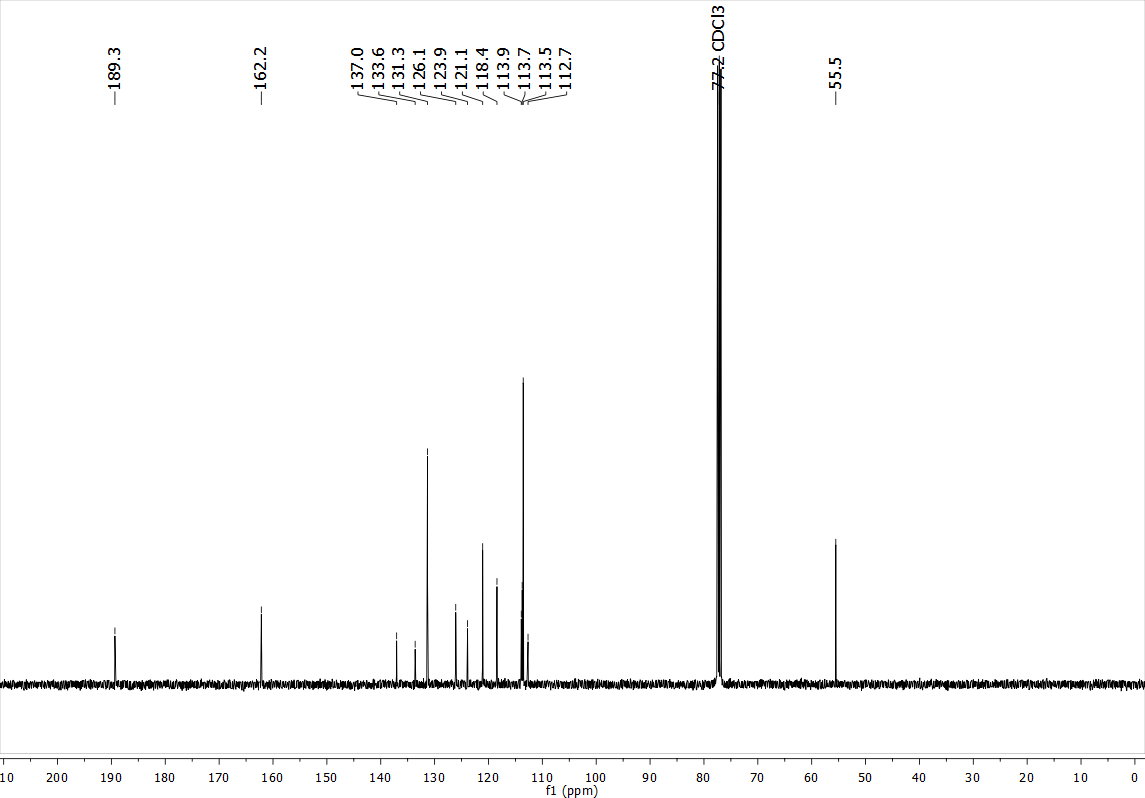
**

**^13^C NMR (100MHz, CDCl_3_) 6d**

**
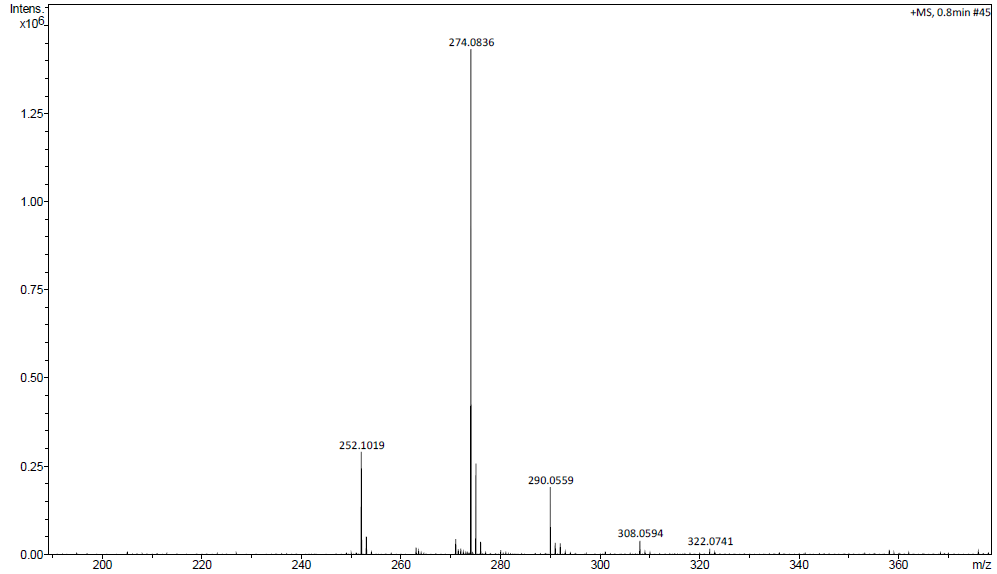
**

**HRAM-MS**  **(ESI+) 6d**

**
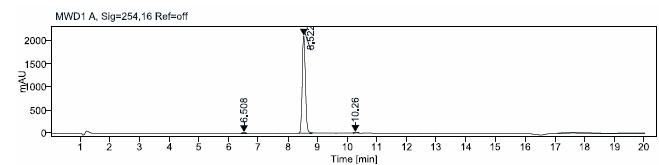
**

**
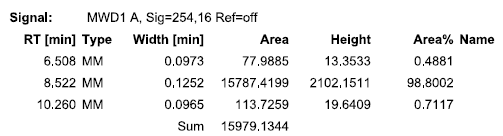
**

**HPLC Purity – 6d (Equipment 1, Method A) R_t_: 8.52 min**

**^
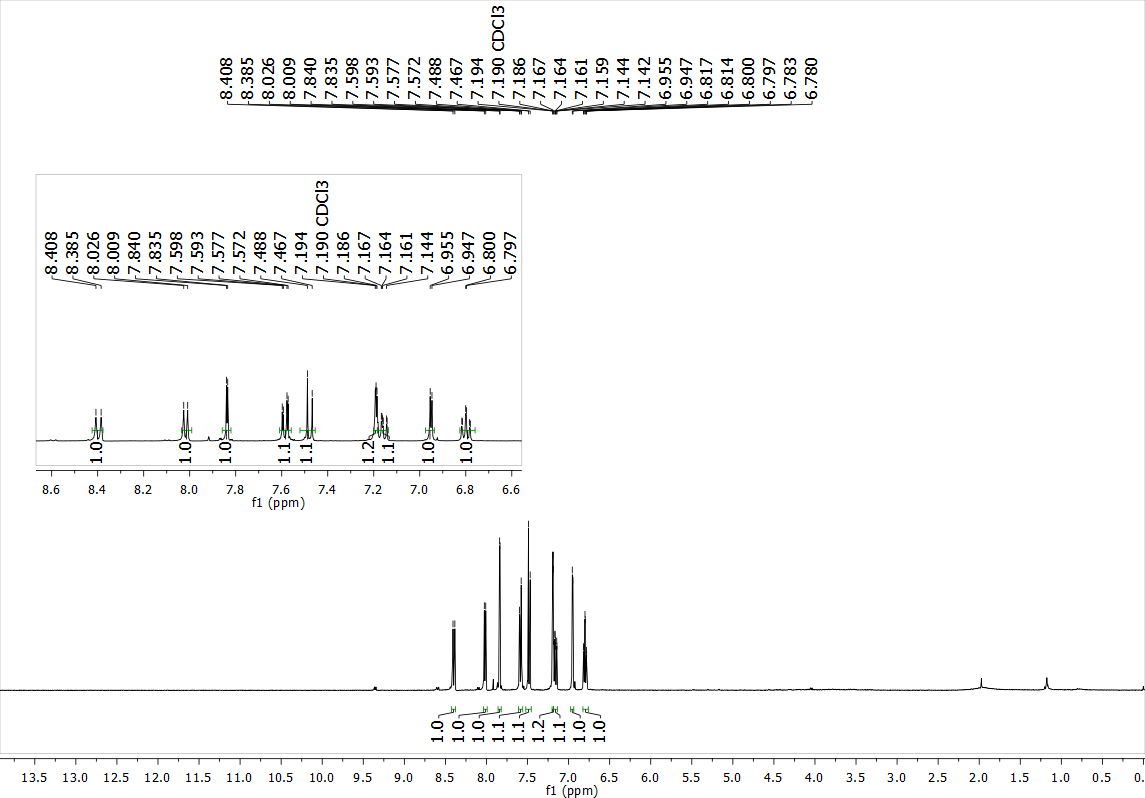
^**

**^1^H NMR (400MHz, CDCl_3_) 6e**

**^^
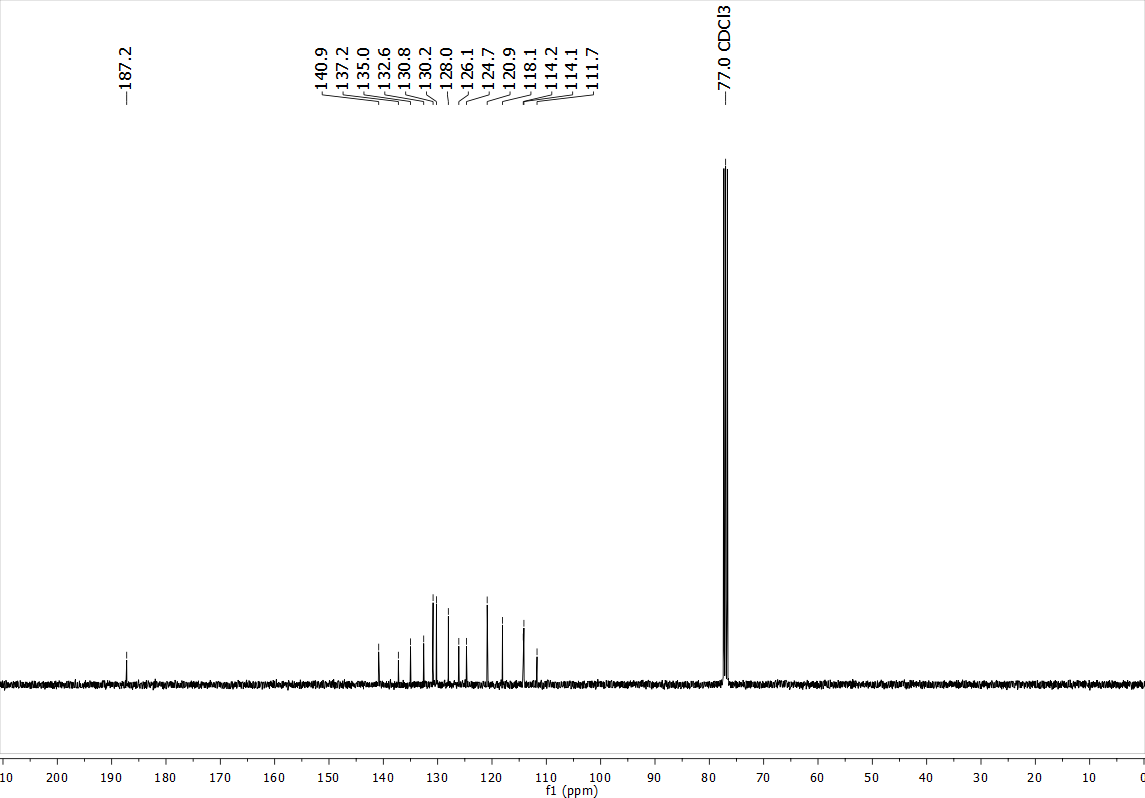
**

**^13^C NMR (100MHz, CDCl_3_) 6e**

**
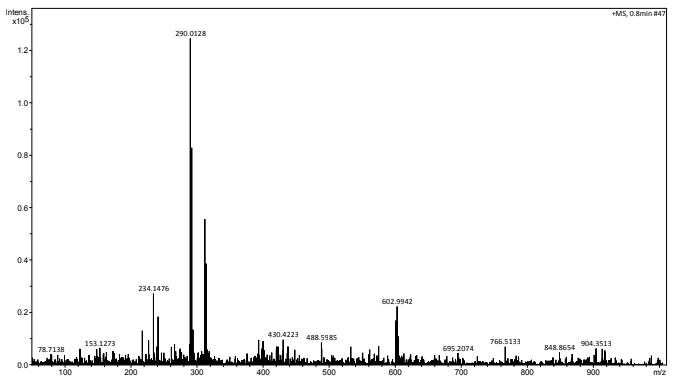
**

**HRAM-MS**  **(ESI+) 6e**

**
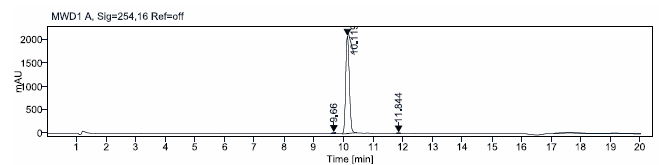
**

**
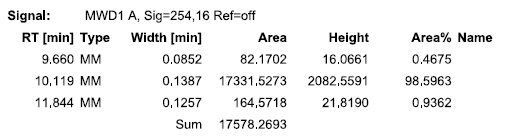
**

**HPLC Purity – 6e (Equipment 1, Method A) R_t_: 10.12 min**

**^
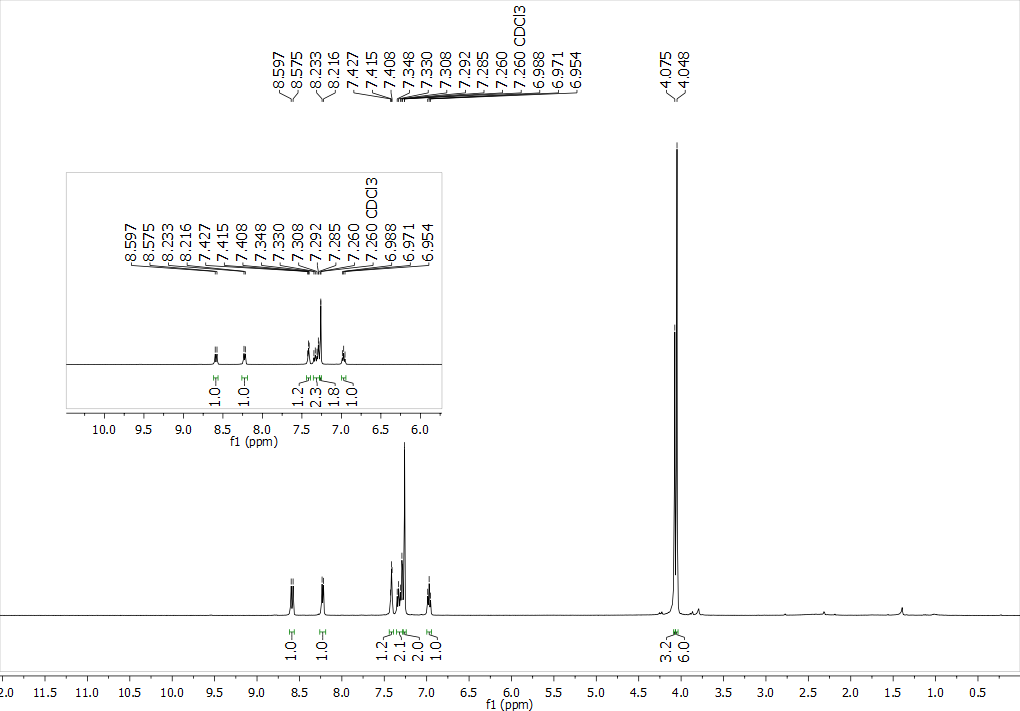
^**

**^1^H NMR (400MHz, CDCl_3_) 6f**

**^^
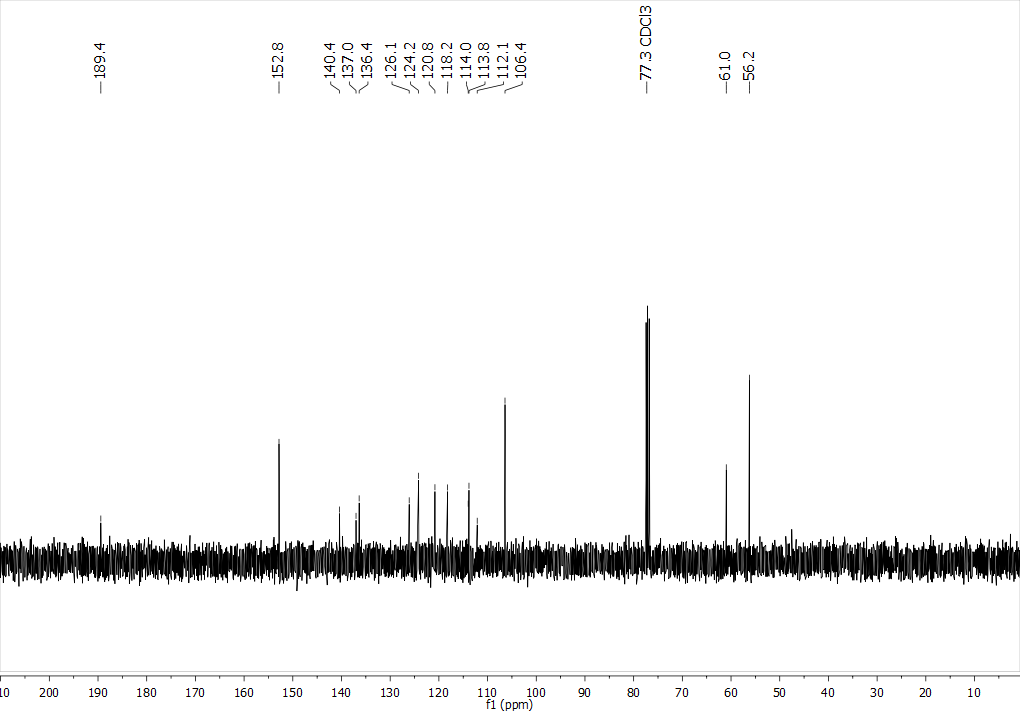
**

**^13^C NMR (100MHz, CDCl_3_) 6f**

**
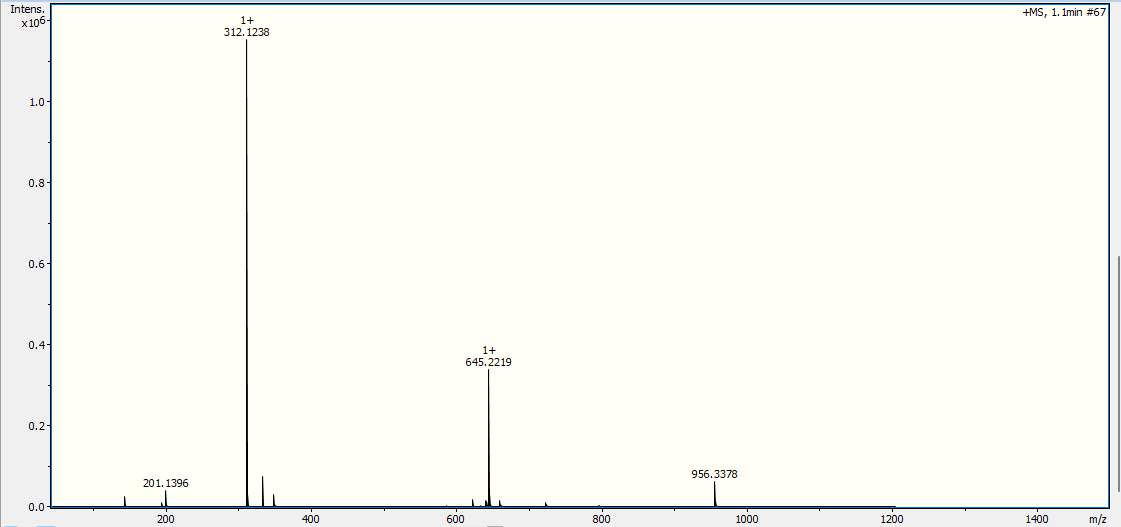
**

**HRAM-MS** **(ESI+) 6f**

**
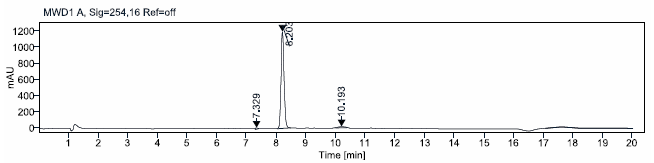
**

**
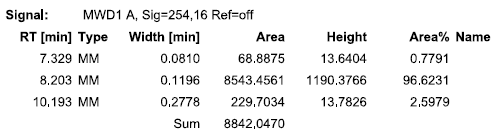
**

**HPLC Purity – 6f (Equipment 1, Method A) R_t_: 8.20 min**

**^
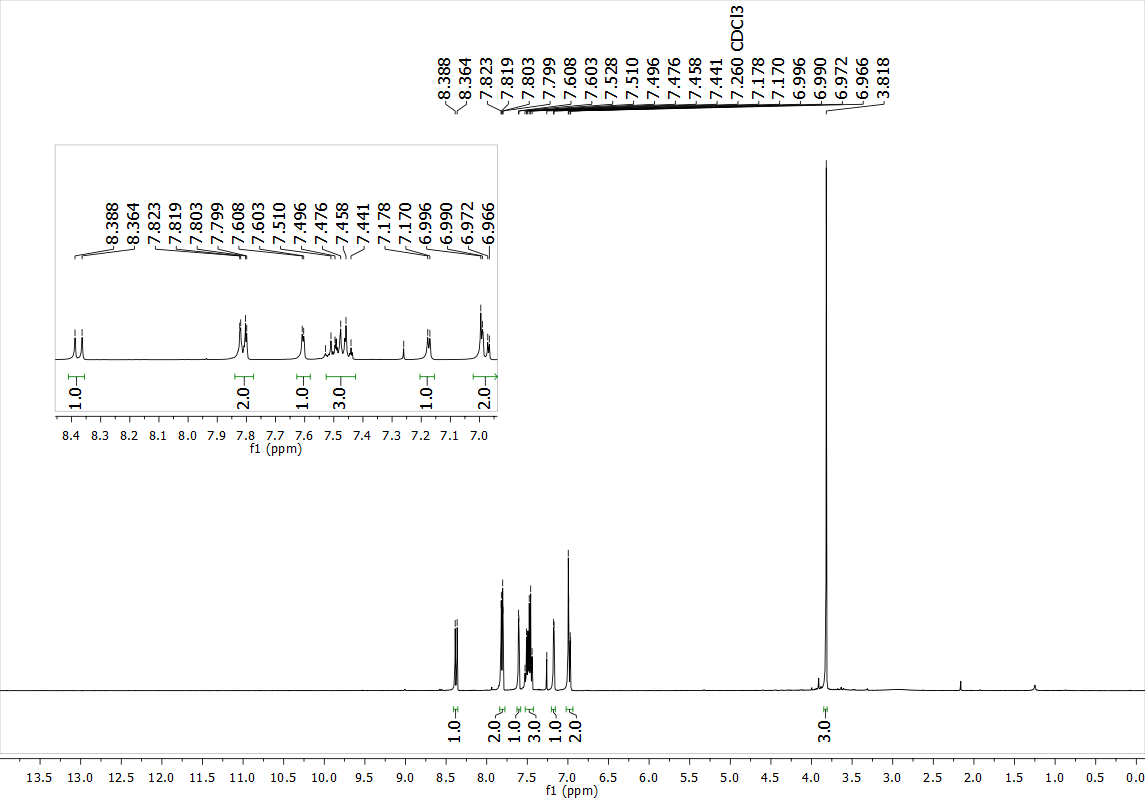
^**

**^1^H NMR (400MHz, CDCl_3_) 7a**

**
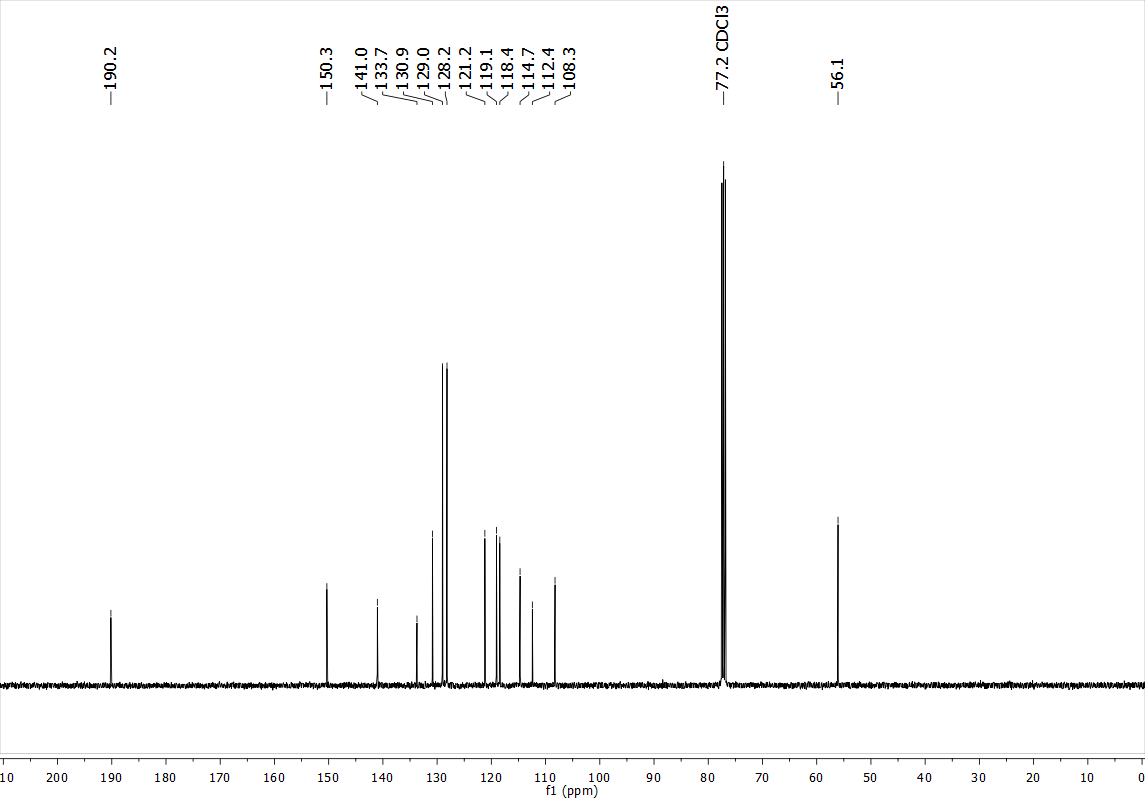
**

**^13^C NMR (100MHz, CDCl_3_) 7a**

**
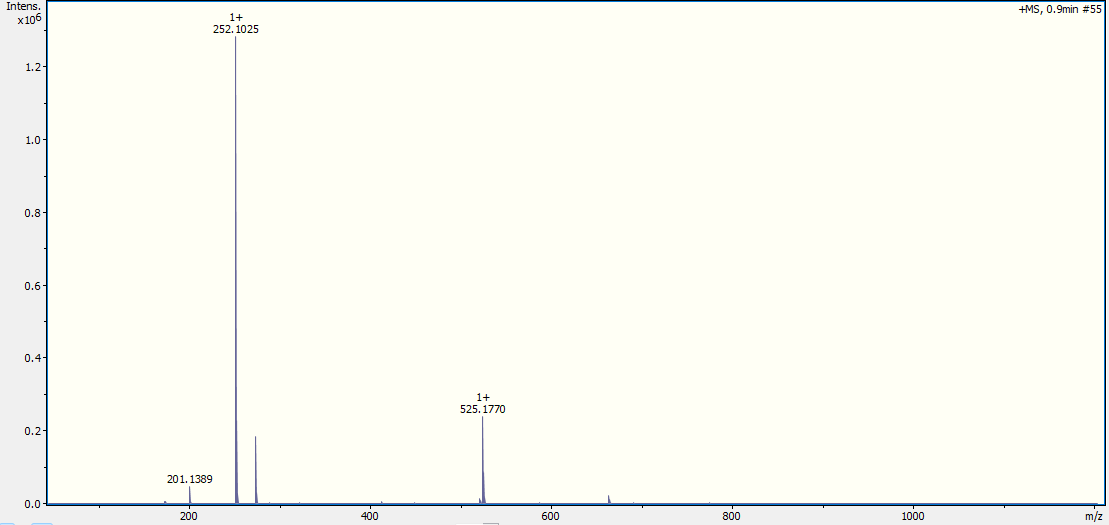
**

**HRAM-MS**  **(ESI+) 7a**

**
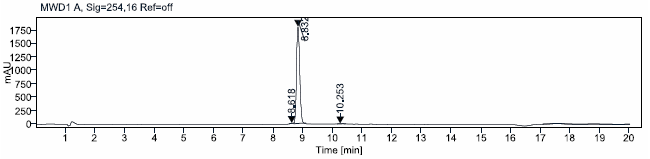
**

**
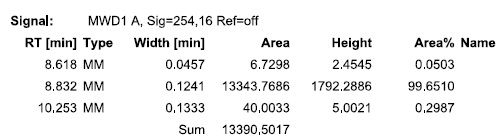
**

**HPLC Purity – 7a (Equipment 1, Method A) R_t_: 8.83 min**

**^
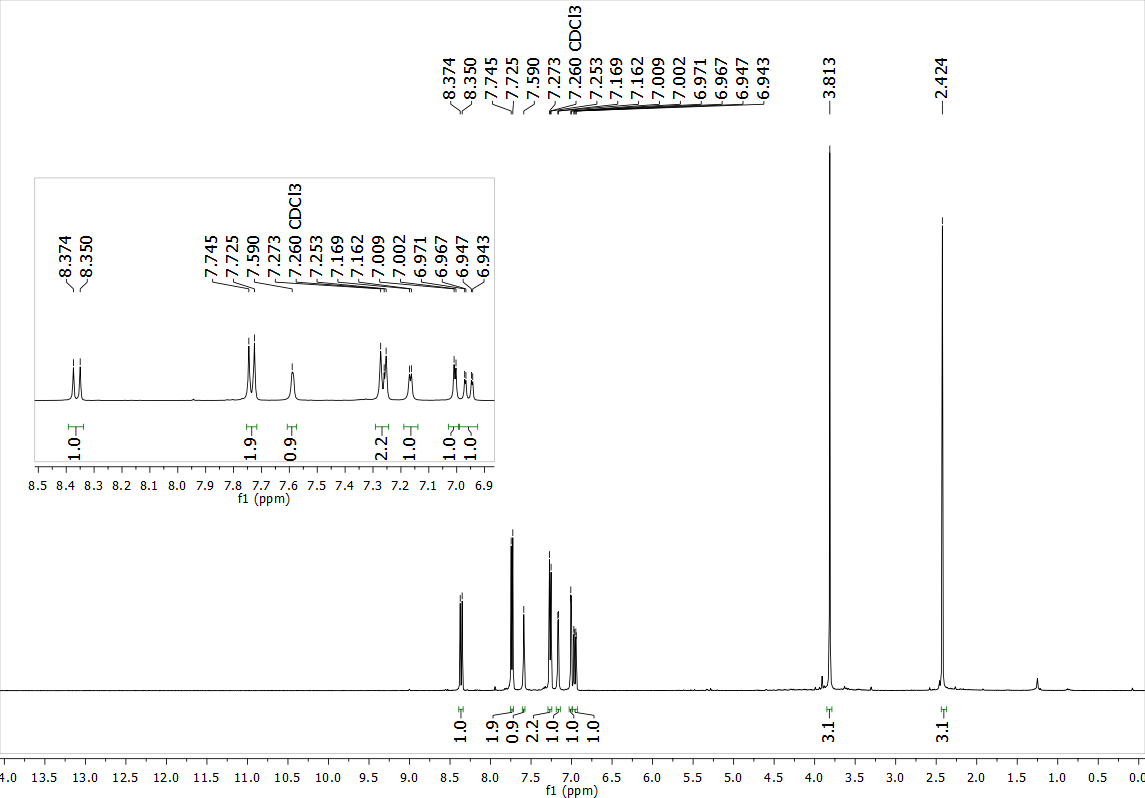
^**

**^1^H NMR (400MHz, CDCl_3_) 7c**

**
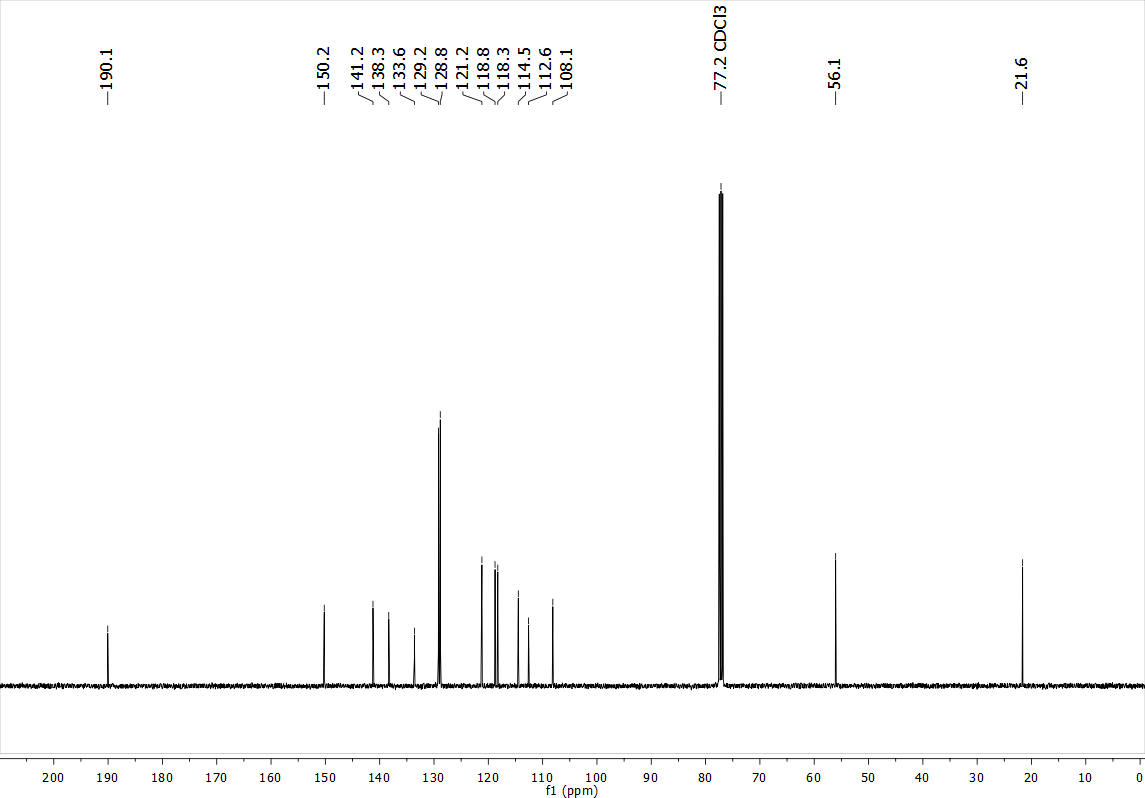
**

**^13^C NMR (100MHz, CDCl_3_) 7c**

**
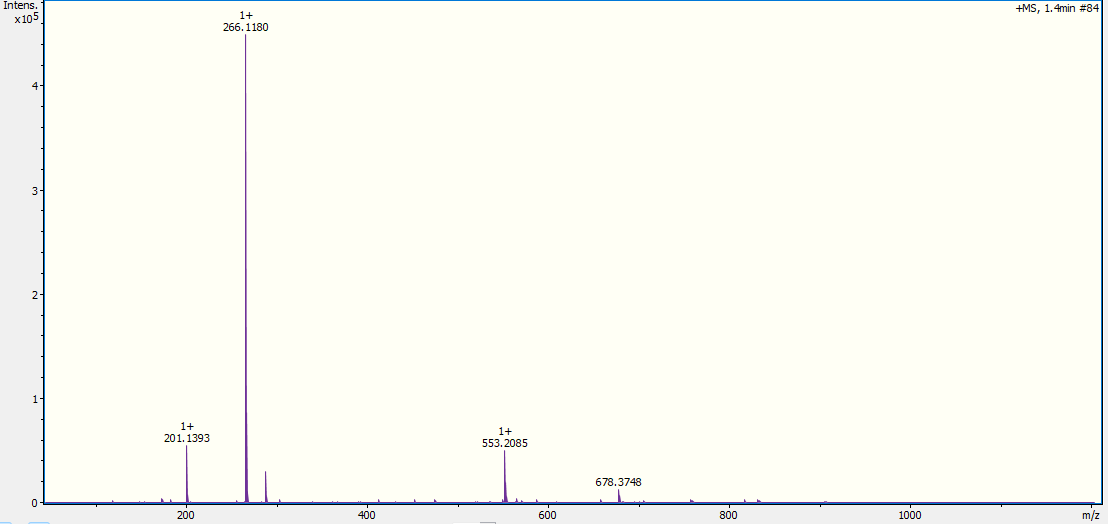
**

**HRAM-MS**  **(ESI+) 7c**

**
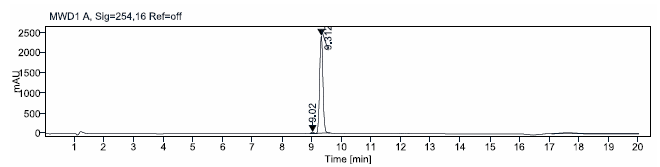
**

**
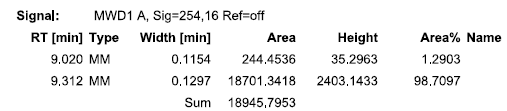
**

**HPLC Purity – 7c (Equipment 1, Method A) R_t_: 9.31 min**

**
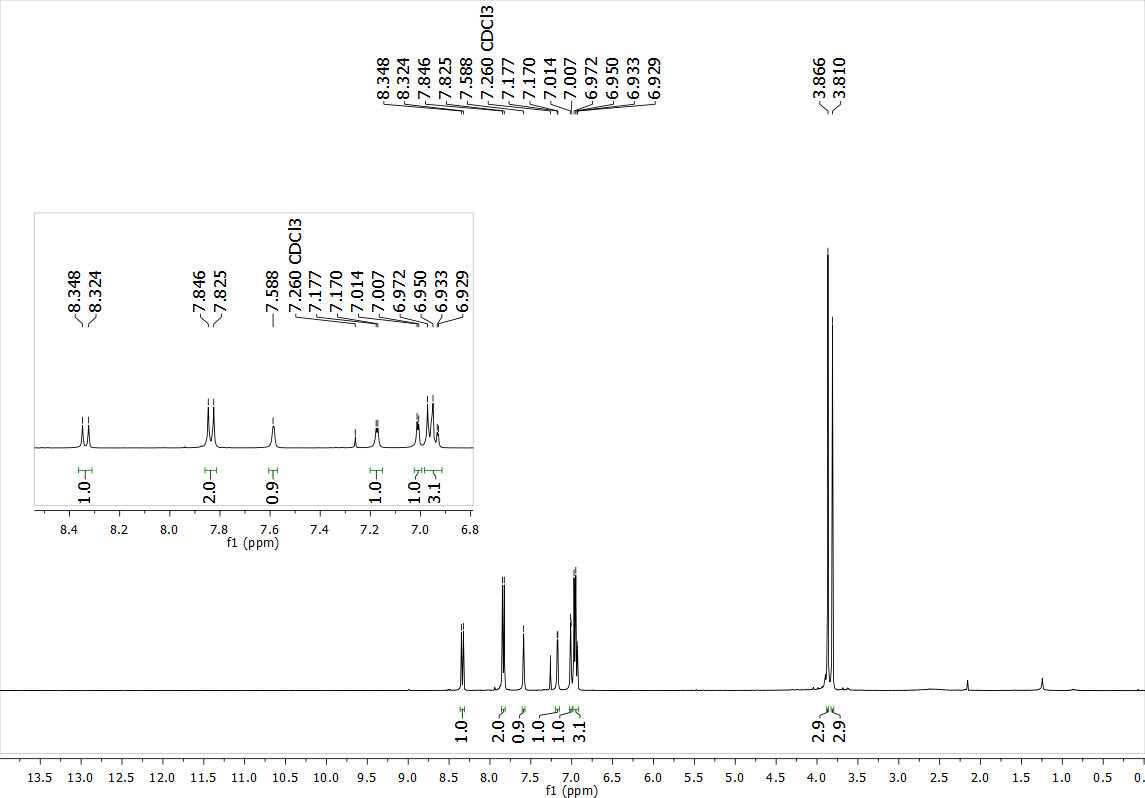
**

**^1^H NMR (400MHz, CDCl_3_) 7d**

**
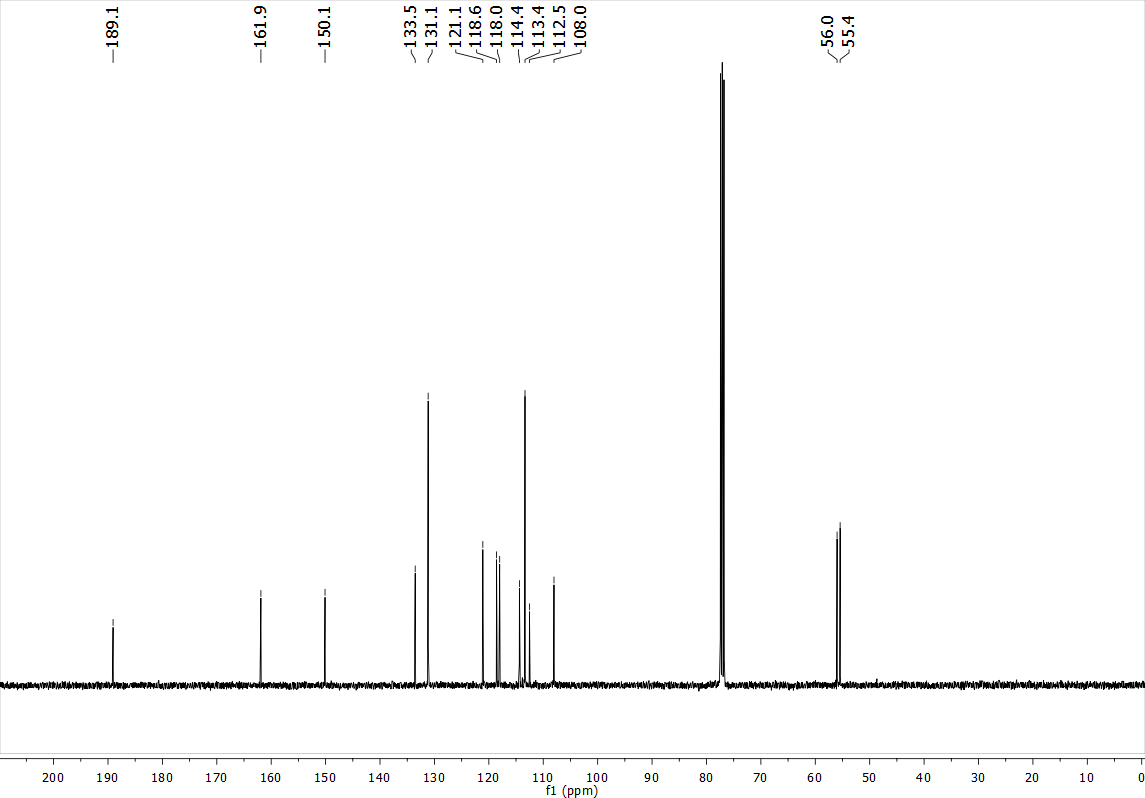
**

**^13^C NMR (100MHz, CDCl_3_) 7d**

**
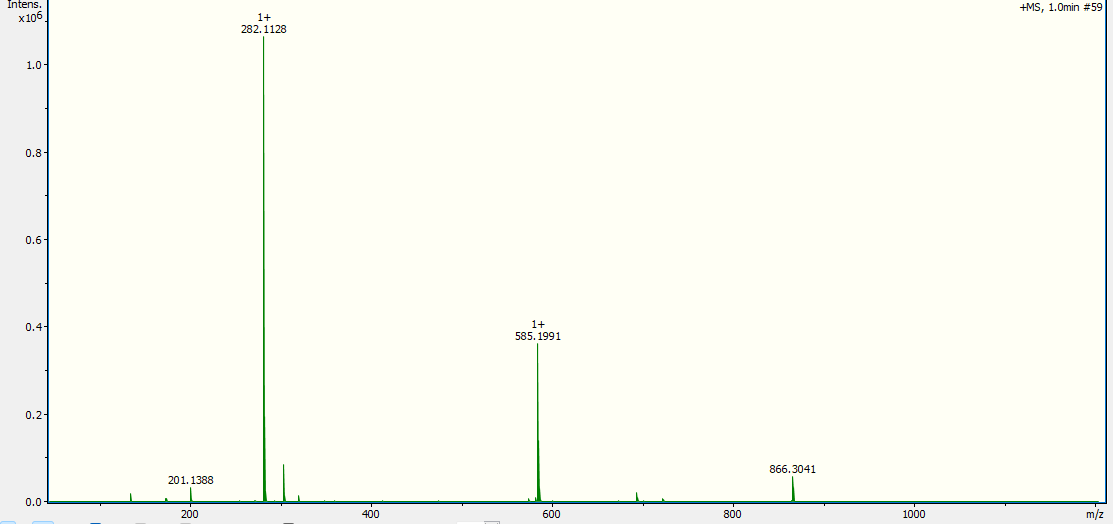
**

**HRAM-MS**  **(ESI+) 7d**

**
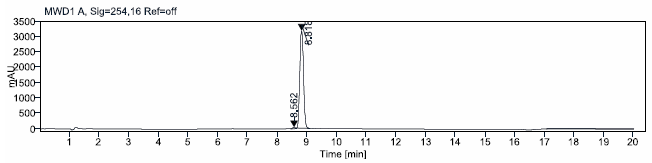
**

**
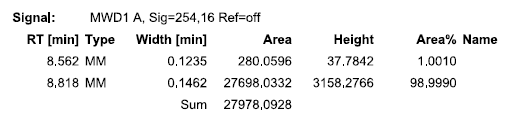
**

**HPLC Purity – 7d (Equipment 1, Method A) R_t_: 8.81 min**

**
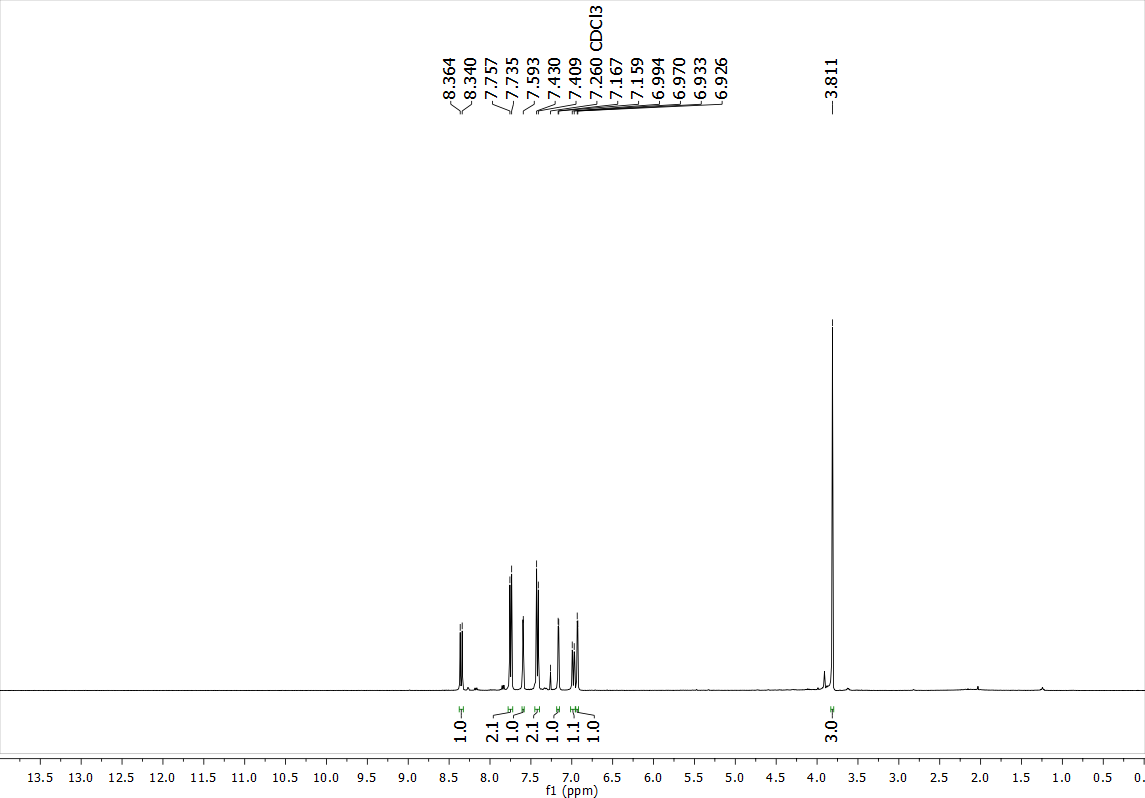
**

**^1^H NMR (400MHz, CDCl_3_) 7b**

**
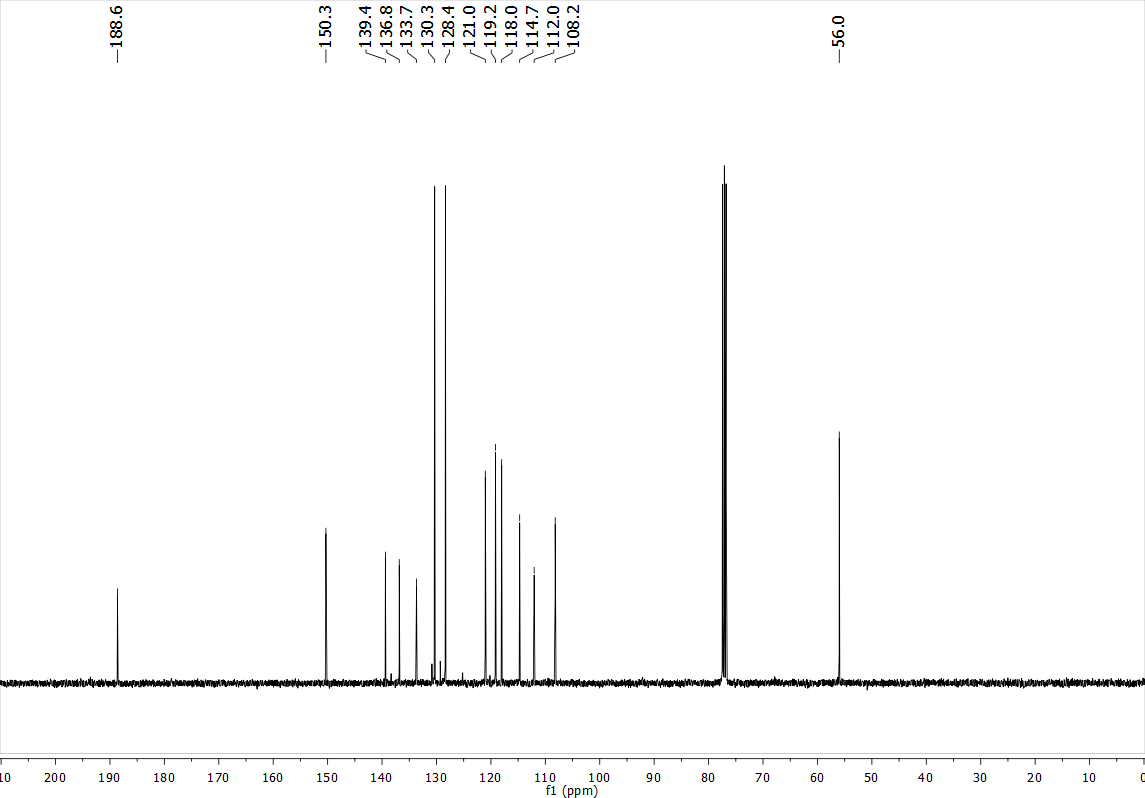
**

**^13^C NMR (100MHz, CDCl_3_) 7b**

**
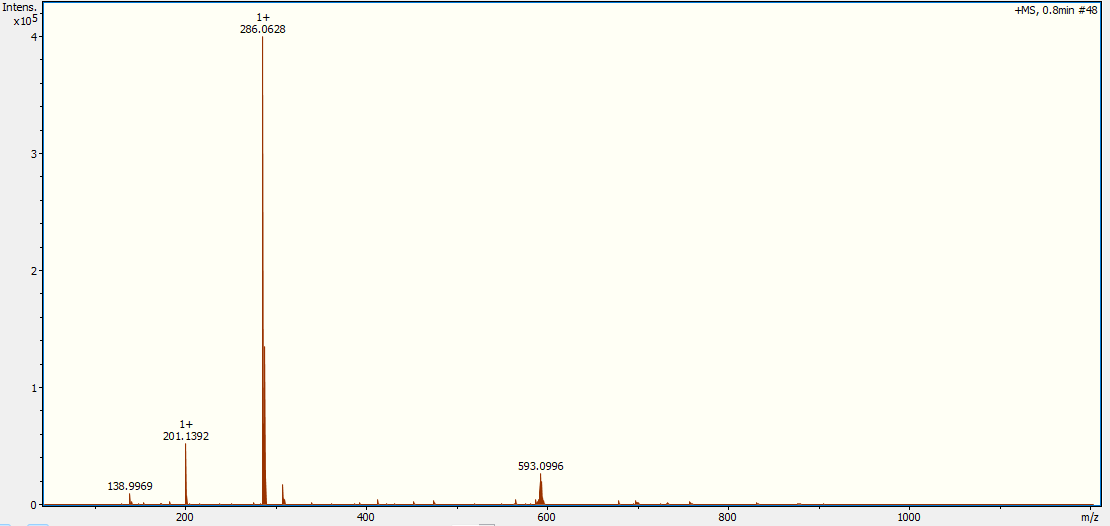
**

**HRAM-MS**  **(ESI+) 7b**

**
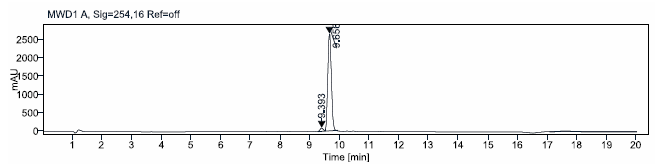
**

**
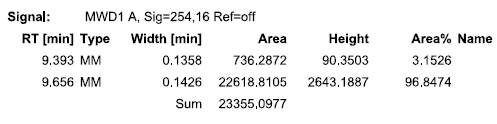
**

**HPLC Purity – 7b (Equipment 1, Method A) R_t_: 7.66 min**

**
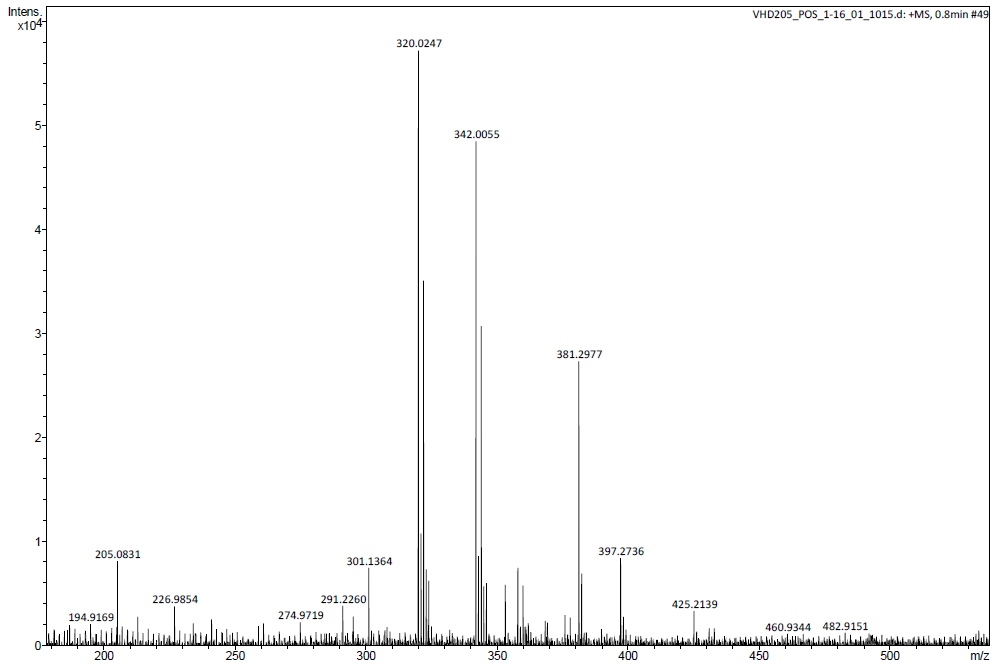
**

**HRAM-MS**  **(ESI+) 7e**

**
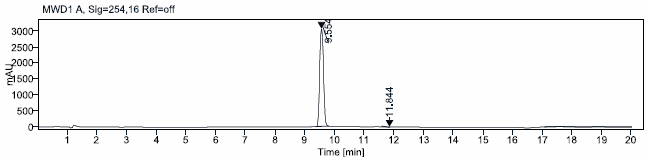
**

**
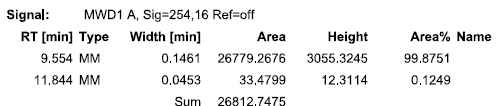
**

**HPLC Purity – 7e (Equipment 1, Method A) R_t_: 9.55 min**

**
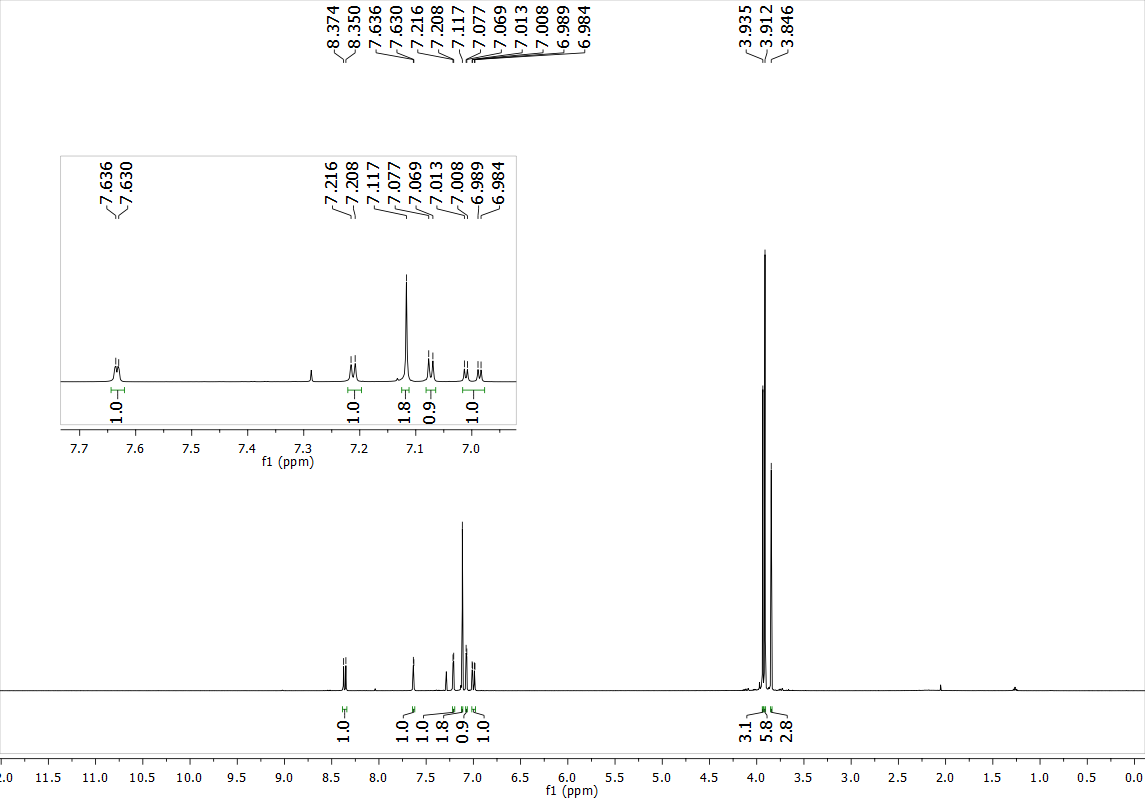
**

**^1^H NMR (400MHz, CDCl_3_) 7f**

**
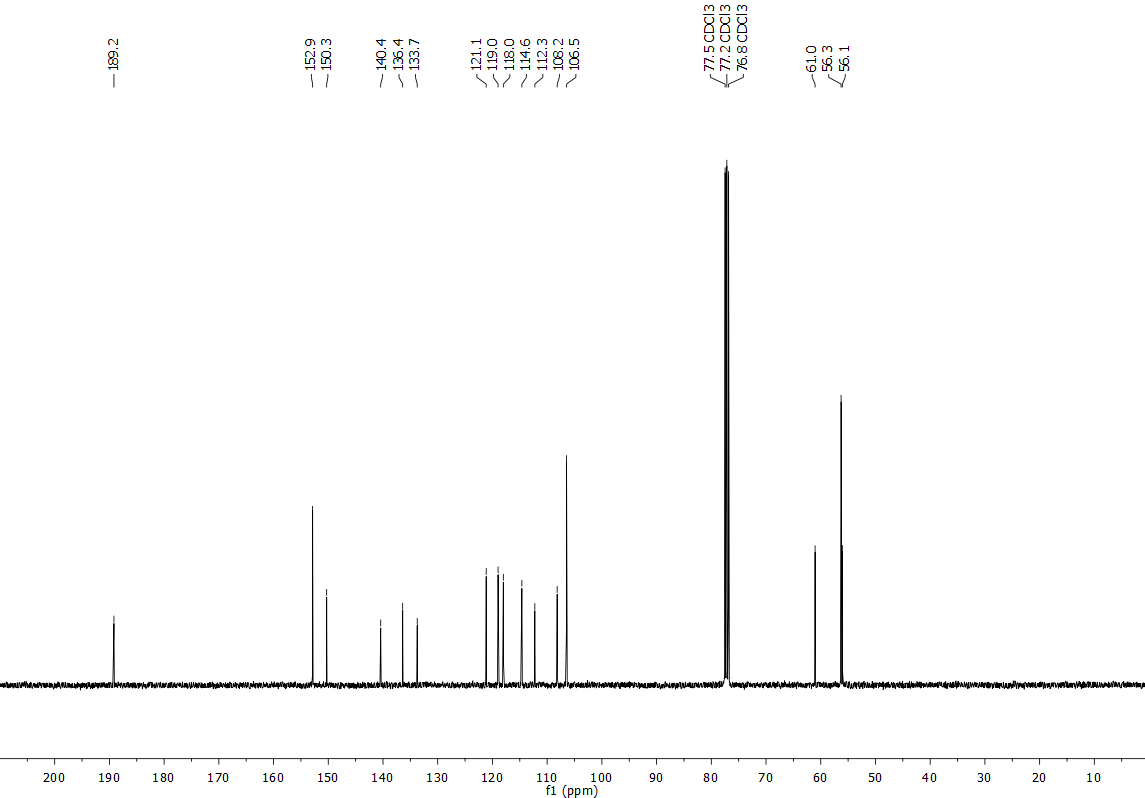
**

**^13^C NMR (100MHz, CDCl_3_) 7f**

**
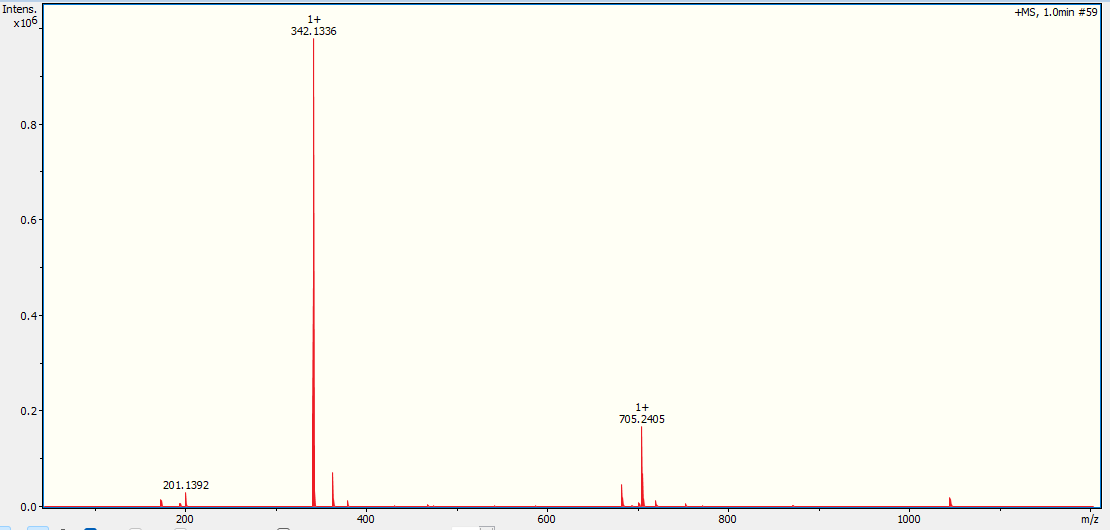
**

**HRAM-MS**  **(ESI+) 7f**

**
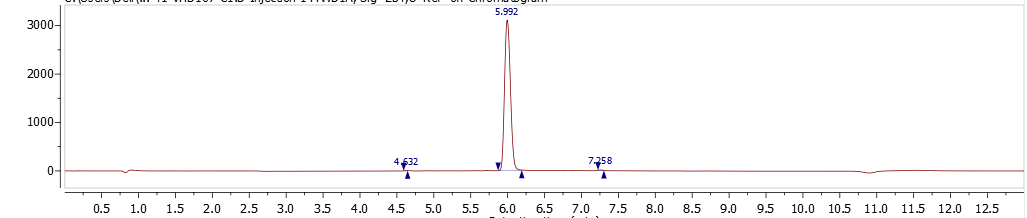
**

| \|  \| RT \| Scan \| Type \| Height \| Area \| Total Height % \| Total Area % \| Start time \| End time \| \| --- \| --- \| --- \| --- \| --- \| --- \| --- \| --- \| --- \| --- \| \| 1 \| 7.258 \| 1089 \| BB \| 6.946 \| 0.348 \| 0.22 \| 0.12 \| 7.224 \| 7.304 \| \| 2 \| 5.992 \| 899 \| BB \| 3105.795 \| 285.915 \| 99.76 \| 99.88 \| 5.872 \| 6.191 \| \| 3 \| 4.632 \| 695 \| BB \| 0.391 \| 0.007 \| 0.01 \| 0.00 \| 4.592 \| 4.646 \| |
| --- | --- | --- | --- | --- | --- | --- | --- | --- | --- | --- | --- | --- | --- | --- | --- | --- | --- | --- | --- | --- | --- | --- | --- | --- | --- | --- | --- | --- | --- | --- | --- | --- | --- | --- | --- | --- | --- | --- | --- | --- |

**HPLC Purity – 7f (Equipment 1, Method A) R_t_: 5.99 min**


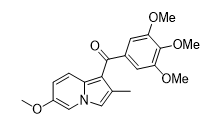
**
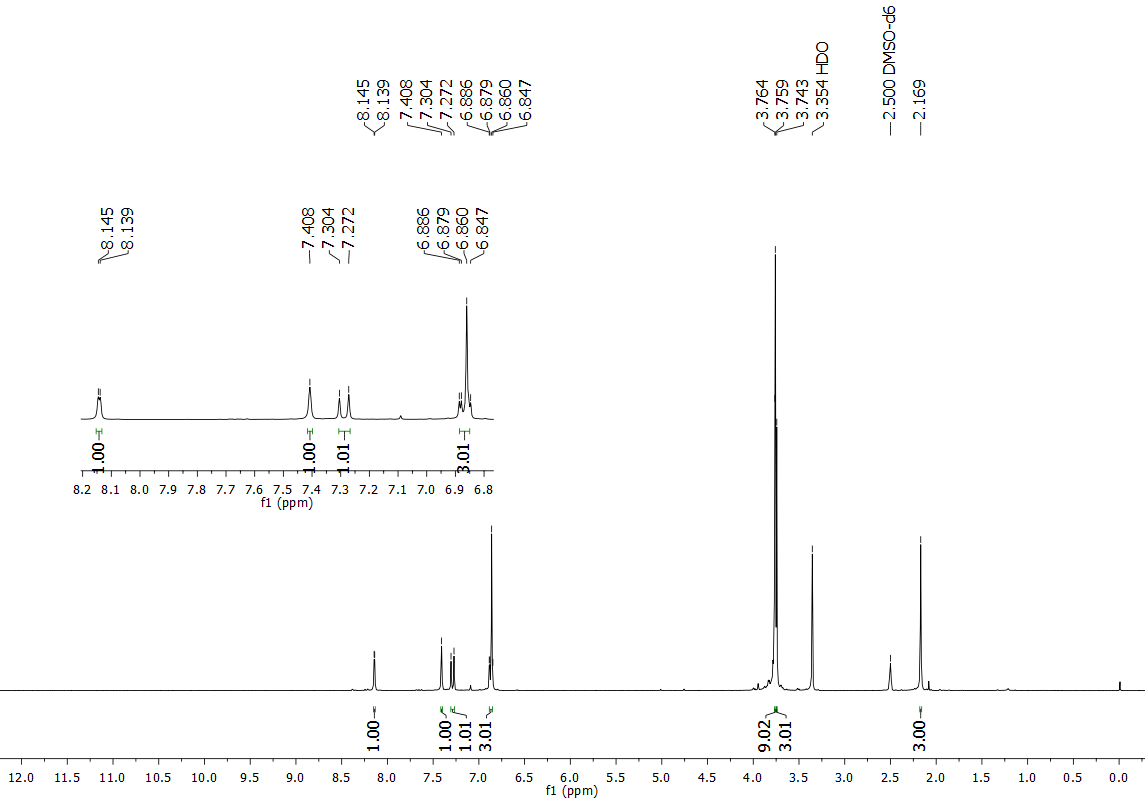
**

**^1^H NMR (300MHz, CDCl3) 8a**


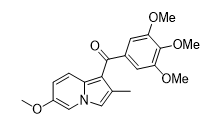

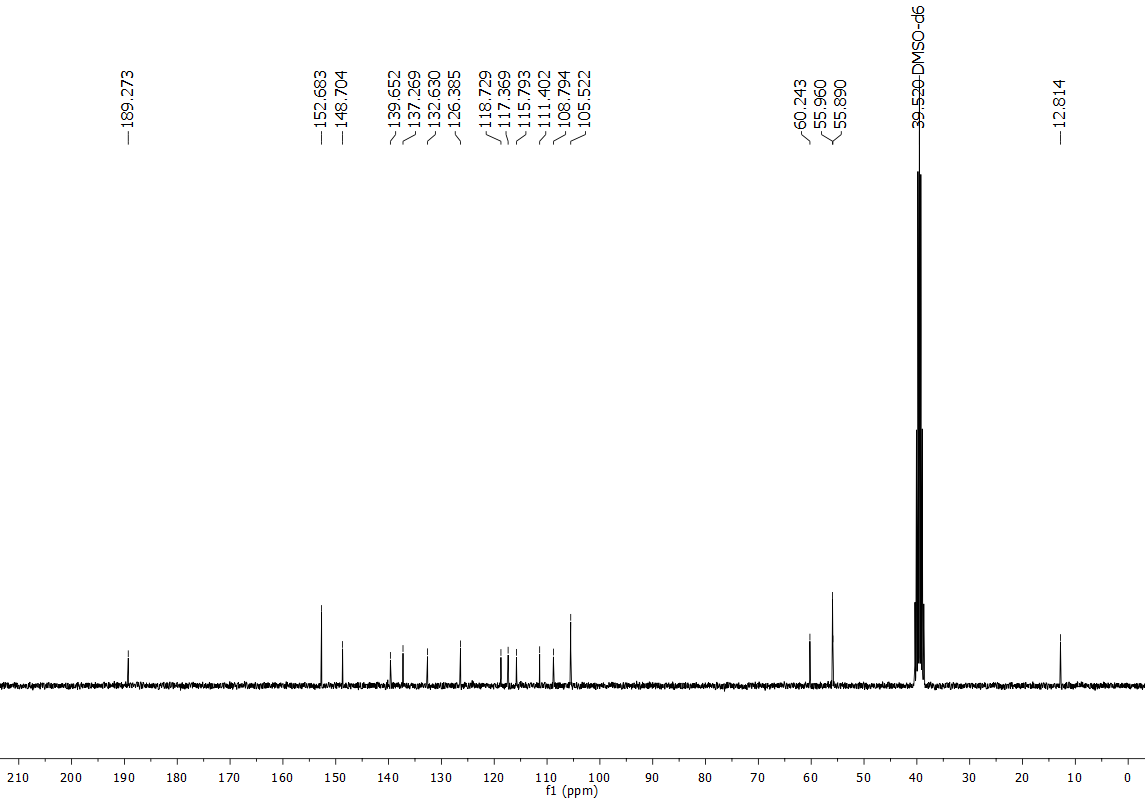


**^13^C NMR (75MHz, CDCl3) 8a**

**
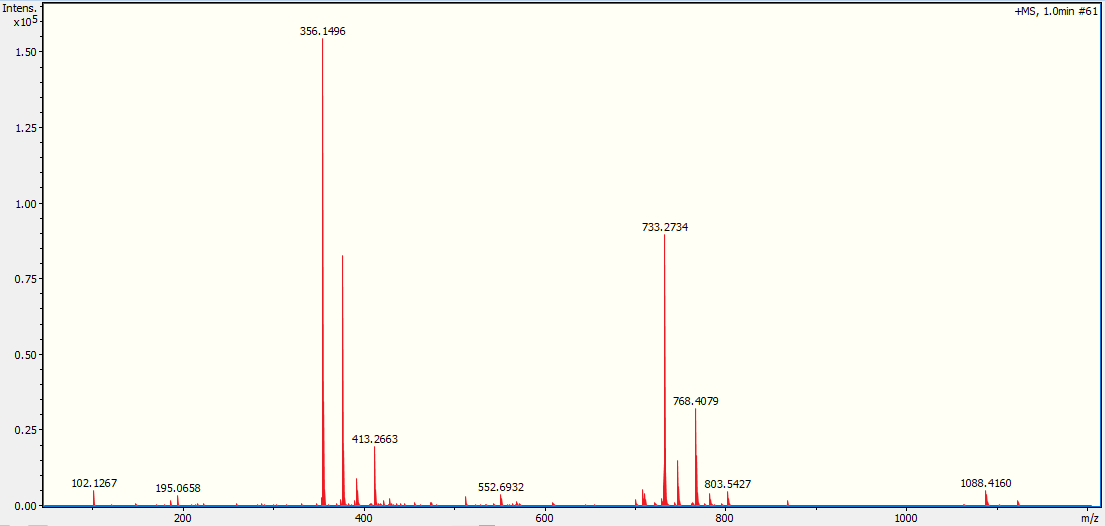
**

**HRAM-MS**  **(ESI+) 8a**

**
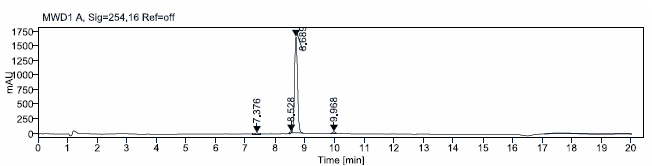
**

**
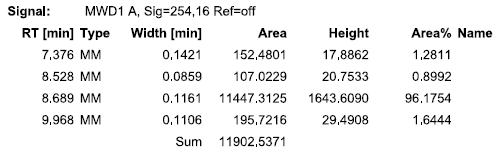
**

**HPLC Purity – 8a (Equipment 1, Method A) R_t_: 8.69 min**

**^
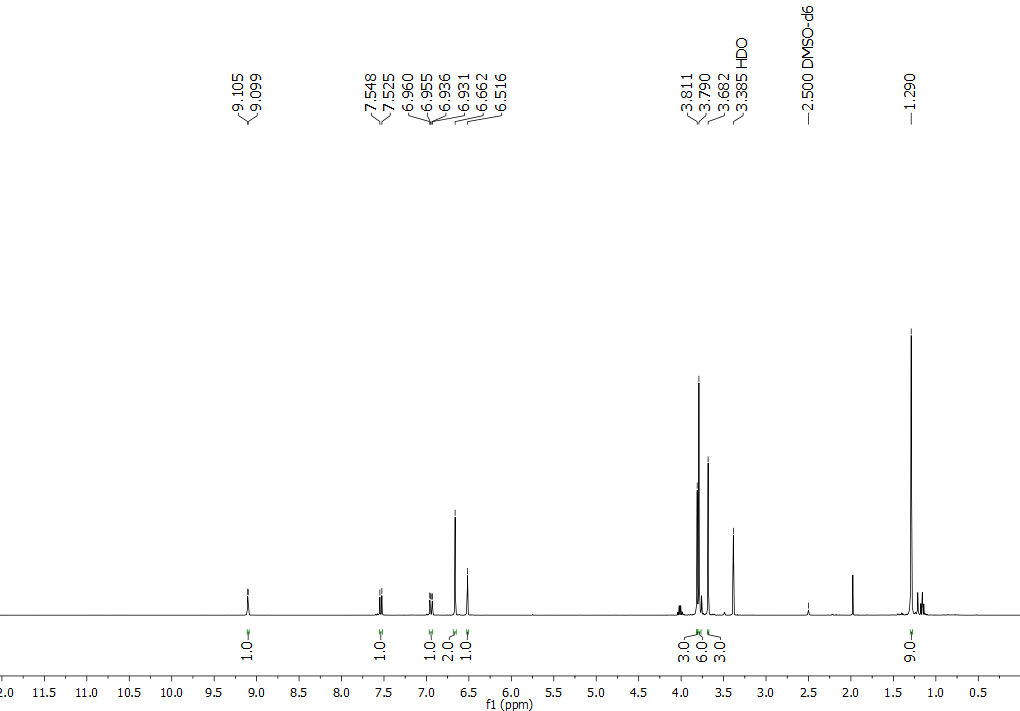
^**

**^1^H NMR (400MHz, DMSO-d_6_) 8b**

**^
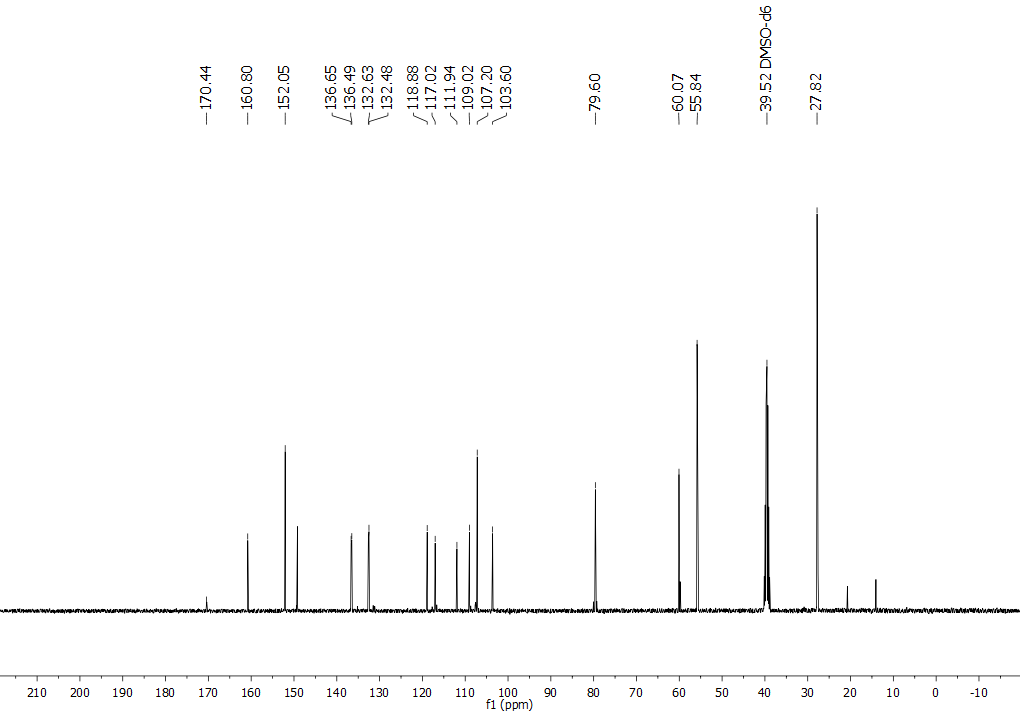
^**

**^13^C NMR (100 MHz, DMSO-d_6_) 8b**

**
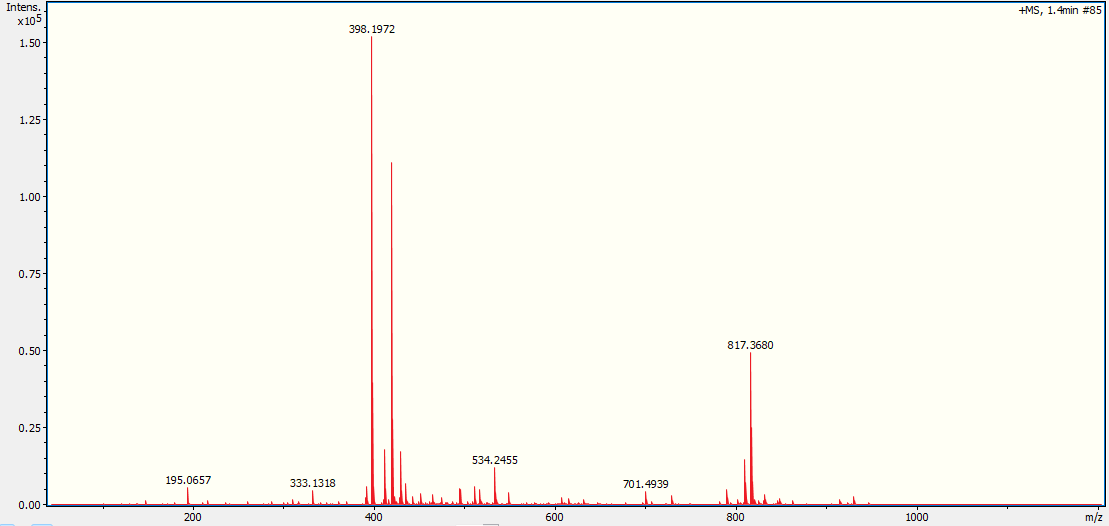
**

**HRAM-MS** **(ESI+) 8b**

**HPLC Purity – 8b (Equipment 2, Method B) R_t_: 21.8 min**

**^1^H NMR (300MHz, DMSO-d_6_) 8c**

**^13^C NMR (75MHz, CDCl3) 8c**

**HRAM-MS**  **(ESI+) 8c**

**HPLC Purity – 8c (Equipment 1, Method A) R_t_: 10.1 min**

**^1^H NMR (300MHz, DMSO-d_6_) 8d**

**^13^C NMR (75MHz, DMSO-d_6_) 8d**

**HRAM-MS**  **(ESI+) 8d**

**HPLC Purity – 8d (Equipment 1, Method A) R_t_: 8.8 min**

**^1^H NMR (300MHz, DMSO-d6) 8e**

**^13^C NMR (75MHz, DMSO-d6) 8e**

**HRAM-MS**  **(ESI+) 8e**

**HPLC Purity – 8e (Equipment 1, Method A) R_t_: 9.3 min**

**^1^H NMR (300MHz, CDCl3) 8f**

**^13^C NMR (75MHz, CDCl3) 8f**

**HRAM-MS**  **(ESI+) 8f**

**HPLC Purity – 8f (Equipment 1, Method A) R_t_: 9.5 min**

**^1^H NMR (300MHz, DMSO-d_6_) 8g**

**^13^C NMR (75MHz, DMSO-d_6_) 8g**

**HRAM-MS**  **(ESI+) 8g**

**HPLC Purity – 8g (Equipment 1, Method A) R_t_: 6.5 min**

**^1^H NMR (300MHz, DMSO-d_6_) 8h**

**^13^C NMR (75MHz, DMSO-d_6_) 8h**

**HRAM-MS**  **(ESI+) 8h**

**HPLC Purity – 8h (Equipment 1, Method A) R_t_: 9.6 min**

**^1^H NMR (300MHz, CDCl_3_) 8j**

**^13^C NMR (75MHz, CDCl_3_) 8j**

**HRAM-MS**  **(ESI+) 8j**

**HPLC Purity – 8j (Equipment 1, Method A) R_t_: 10.4 min**

**^1^H NMR (500MHz, DMSO-d_6_) 8i**

**^13^C NMR (125MHz, DMSO-d_6_) 8i**

**HRAM-MS**  **(ESI+) 8i**

**HPLC Purity – 8i (Equipment 1, Method A) R_t_: 9.8 min**

**^1^H NMR (500MHz, DMSO-d_6_) 8j**

**^13^C NMR (125MHz, DMSO-d_6_) 8j**

**HRAM-MS**  **(ESI+) 8j**

**HPLC Purity – 8c (Equipment 1, Method A) R_t_: 9.36 min**
