# Supplementary material for: Design, Synthesis, and Selective Antiproliferative Activity of Indolizine Derivatives as Microtubule Destabilizers
Source: Arch Pharm (Weinheim). 2025 Dec 3;358(12):e70161. doi: 10.1002/ardp.70161 (PMC12673918; doi:10.1002/ardp.70161)
Supplement: Supplementary file 2 — ArchPharm SupplMat InChI 24 10 revised. [file ARDP-358-e70161-s001.doc]

Supplemental Material: Design and Functionalization of Indolizine Derivatives as Potent Cytotoxic Agents Targeting Microtubules

Victor Hugo Catricala Fernandes^1,3^, Maitê Bueno Giometti^1^, Franco Jazon Caires^1^, Gabriel de Paula Bueno^1^, Gabriel da Silva^2^, Andréia Machado Leopoldino^2^, Anna Junker^3^, Giuliano Cesar Clososki^1^,*

1 Research Center on Natural and Synthetic Products, Department of Biomolecular Sciences, Faculty of Pharmaceutical Sciences of Ribeirão Preto, Ribeirão Preto, Brazil.

2 Department of Clinical Analyses, Toxicological and Food Sciences, Faculty of Pharmaceutical Sciences of Ribeirão Preto, Ribeirão Preto, Brazil.

3 Werner Siemens Imaging Center, Department of Preclinical Imaging and Radiopharmacy, University of Tuebingen, Roentgenweg 13, 72076 Tuebingen, Germany.

*Correspondence:

Prof. Dr. Giuliano Cesar Clososki, Department of Biomolecular Sciences, Faculty of Pharmaceutical Sciences of Ribeirão Preto, Av. Prof. Zeferino Vaz, S/N, 14040-230, Ribeirão Preto, Brazil.

Email: gclososki@usp.br

| **Compound No.** | **InChI** | **% Cellular growth inhibition (at 50 µM)^a^** | | | **IC_50_ (nM, Cellular growth inhibition)**^b^ | | | | **IC_50_ (µM)**  **Tubulin polymerization assay^C^** |
| --- | --- | --- | --- | --- | --- | --- | --- | --- | --- |
|  |  | **CAL-27** | **BT-20** | **HGC-27** | **CAL-27** | **BT-20** | **HGC-27** | **GNP15** |  |
| **4a** | InChI=1S/C13H11NO/c15-13(11-6-2-1-3-7-11)10-12-8-4-5-9-14-12/h1-9H,10H2 | 12.2 ± 2.0 | 7.5 ± 0.5 | 2.4 ± 0.9 | NT | | | | NT |
| **4b** | InChI=1S/C13H10ClNO/c14-11-6-4-10(5-7-11)13(16)9-12-3-1-2-8-15-12/h1-8H,9H2 | 11.4 ± 0.4 | 17.3 ± 1.8 | 16.1 ± 1.6 | NT | | | | NT |
| **4c** | InChI=1S/C14H13NO/c1-11-5-7-12(8-6-11)14(16)10-13-4-2-3-9-15-13/h2-9H,10H2,1H3 | 9.0 ± 0.8 | 13.7 ± 0.7 | 14.1 ± 1.8 | NT | | | | NT |
| **4d** | InChI=1S/C14H13NO2/c1-17-13-7-5-11(6-8-13)14(16)10-12-4-2-3-9-15-12/h2-9H,10H2,1H3 | 12.6 ± 1.2 | 17.4 ± 0.6 | 17.3 ± 1.2 | NT | | | | NT |
| **4e** | InChI=1S/C13H9Cl2NO/c14-11-5-4-9(7-12(11)15)13(17)8-10-3-1-2-6-16-10/h1-7H,8H2 | 22.3 ± 1.3 | 43.3 ± 0.6 | 36.9 ± 0.8 | NT | | | | NT |
| **6a** | InChI=1S/C15H11NO/c17-15(12-6-2-1-3-7-12)13-9-11-16-10-5-4-8-14(13)16/h1-11H | 15 ± 0.8 | 26 ± 1.0 | 20 ± 1.1 | NT | | | | NT |
| **6b** | InChI=1S/C15H10ClNO/c16-12-6-4-11(5-7-12)15(18)13-8-10-17-9-2-1-3-14(13)17/h1-10H | 25 ± 0.4 | 32 ± 0.8 | 26 ± 1.0 | NT | | | | NT |
| **6c** | InChI=1S/C16H13NO/c1-12-5-7-13(8-6-12)16(18)14-9-11-17-10-3-2-4-15(14)17/h2-11H,1H3 | 22 ± 2.6 | 28 ± 1.3 | 20 ± 0.6 | NT | | | | NT |
| **6d** | InChI=1S/C16H13NO2/c1-19-13-7-5-12(6-8-13)16(18)14-9-11-17-10-3-2-4-15(14)17/h2-11H,1H3 | 18 ± 0.6 | 28 ± 0.5 | 24 ± 1.1 | NT | | | | NT |
| **6e** | InChI=1S/C15H9Cl2NO/c16-12-5-4-10(9-13(12)17)15(19)11-6-8-18-7-2-1-3-14(11)18/h1-9H | 42 ± 0.8 | 40 ± 0.4 | 41 ± 1.0 | NT | | | | NT |
| **7a** | InChI=1S/C16H13NO2/c1-19-13-7-8-15-14(9-10-17(15)11-13)16(18)12-5-3-2-4-6-12/h2-11H,1H3 | 20 ± 0.9 | 20 ± 0.4 | 18 ± 0.5 | NT | | | | NT |
| **7b** | InChI=1S/C17H15NO2/c1-12-3-5-13(6-4-12)17(19)15-9-10-18-11-14(20-2)7-8-16(15)18/h3-11H,1-2H3 | 23 ± 1.0 | 8 ± 1.3 | 10 ± 1.4 | NT | | | | NT |
| **7c** | InChI=1S/C17H15NO3/c1-20-13-5-3-12(4-6-13)17(19)15-9-10-18-11-14(21-2)7-8-16(15)18/h3-11H,1-2H3 | 20 ± 0.7 | 15 ± 0.5 | 13 ± 0.7 | NT | | | | NT |
| **7d** | InChI=1S/C16H12ClNO2/c1-20-13-6-7-15-14(8-9-18(15)10-13)16(19)11-2-4-12(17)5-3-11/h2-10H,1H3 | 15 ± 1.1 | 7 ± 0.9 | 1 ± 1.0 | NT | | | | NT |
| **7e** | InChI=1S/C16H11Cl2NO2/c1-21-11-3-5-15-12(6-7-19(15)9-11)16(20)10-2-4-13(17)14(18)8-10/h2-9H,1H3 | **70 ± 3.8** | **53 ± 1.8** | **68 ± 3.4** | 11,860 ± 1,180 | 12,350 ± 353 | 14,095 ± 1,138 | > 50,000 | NT |
| **6f** | InChI=1S/C18H17NO4/c1-21-15-10-12(11-16(22-2)18(15)23-3)17(20)13-7-9-19-8-5-4-6-14(13)19/h4-11H,1-3H3 | **57 ± 0.9** | **43 ± 0.5** | **38 ± 1.6** | NT | | | | NT |
| **7f** | InChI=1S/C19H19NO5/c1-22-13-5-6-15-14(7-8-20(15)11-13)18(21)12-9-16(23-2)19(25-4)17(10-12)24-3/h5-11H,1-4H3 | **58 ± 1.3** | **43 ± 0.8** | **43 ± 0.7** | 475 ± 105 | 105 ± 21 | 111 ± 28 | > 50,000 | 14.8 |
| **4f** | InChI=1S/C16H17NO4/c1-19-14-8-11(9-15(20-2)16(14)21-3)13(18)10-12-6-4-5-7-17-12/h4-9H,10H2,1-3H3 | 20 ± 2.2 | 19 ± 1.2 | 10 ± 0.7 | NT | | | | NT |
| **5f** | InChI=1S/C17H19NO5/c1-20-13-6-5-12(18-10-13)9-14(19)11-7-15(21-2)17(23-4)16(8-11)22-3/h5-8,10H,9H2,1-4H3 | 67 ± 2.7 | 52 ± 4.2 | 48 ± 4.9 | NT | | | | NT |
| **8a** | InChI=1S/C20H21NO5/c1-12-10-21-11-14(23-2)6-7-15(21)18(12)19(22)13-8-16(24-3)20(26-5)17(9-13)25-4/h6-11H,1-5H3 | 64 ± 2.7 | 52 ± 5.0 | 41 ± 4.3 | 410 ± 212 | 325 ± 92 | 370 ± 28 | > 50,000 | NT |
| **8b** | InChI=1S/C23H27NO5/c1-23(2,3)16-13-24-12-15(26-4)8-9-17(24)20(16)21(25)14-10-18(27-5)22(29-7)19(11-14)28-6/h8-13H,1-7H3 | 84 ± 3.2 | 62 ± 0.5 | 46 ± 6.0 | NT | | | | NT |
| **8c** | InChI=1S/C21H23NO6/c1-6-28-18-12-22-11-14(24-2)7-8-15(22)19(18)20(23)13-9-16(25-3)21(27-5)17(10-13)26-4/h7-12H,6H2,1-5H3 | 56 ± 4.6 | 55 ± 4.7 | 43 ± 3.5 | NT | | | | NT |
| **8d** | InChI=1S/C22H23NO7/c1-6-30-22(25)15-12-23-11-14(26-2)7-8-16(23)19(15)20(24)13-9-17(27-3)21(29-5)18(10-13)28-4/h7-12H,6H2,1-5H3 | 66 ± 0.8 | 55 ± 4.7 | 44 ± 1.9 | NT | | | | NT |
| **8e** | InChI=1S/C20H18F3NO5/c1-26-12-5-6-14-17(13(20(21,22)23)10-24(14)9-12)18(25)11-7-15(27-2)19(29-4)16(8-11)28-3/h5-10H,1-4H3 | 76 ± 1.3 | 62 ± 2.3 | 53 ± 2.2 | 117 ± 32 | 75 ± 21 | 105 ± 49 | > 50,000 | 9.3 |
| **8f** | InChI=1S/C25H23NO5/c1-28-18-10-11-20-23(19(15-26(20)14-18)16-8-6-5-7-9-16)24(27)17-12-21(29-2)25(31-4)22(13-17)30-3/h5-15H,1-4H3 | 69 ± 0.4 | 55 ± 1.8 | 49 ± 4.0 | NT | | | | NT |
| **8g** | InChI=1S/C24H22N2O5/c1-28-16-8-9-19-22(17(14-26(19)13-16)18-7-5-6-10-25-18)23(27)15-11-20(29-2)24(31-4)21(12-15)30-3/h5-14H,1-4H3 | 68 ± 1.0 | 54 ± 2.8 | 44 ± 0.4 | NT | | | | NT |
| **8h** | InChI=1S/C22H25NO5/c1-6-7-14-12-23-13-16(25-2)8-9-17(23)20(14)21(24)15-10-18(26-3)22(28-5)19(11-15)27-4/h8-13H,6-7H2,1-5H3 | 90 ± 1.8 | 71 ± 2.8 | 70 ± 3.3 | 75 ± 35 | 47 ± 18 | 90 ± 56 | > 50,000 | 10.9 |
| **8i** | InChI=1S/C24H29NO5/c1-6-7-8-9-16-14-25-15-18(27-2)10-11-19(25)22(16)23(26)17-12-20(28-3)24(30-5)21(13-17)29-4/h10-15H,6-9H2,1-5H3 | 57 ± 2.5 | 38 ± 3.5 | 61 ± 1.5 | NT | | | | NT |
| **8j** | InChI=1S/C26H25NO5/c1-16-6-8-17(9-7-16)20-15-27-14-19(29-2)10-11-21(27)24(20)25(28)18-12-22(30-3)26(32-5)23(13-18)31-4/h6-15H,1-5H3 | 73 ± 4.0 | 61 ± 2.0 | 71 ± 2.0 | 465 ± 35 | 300 ± 85 | 465 ± 35 | > 50,000 | NT |
| **8k** | InChI=1S/C26H25NO6/c1-29-18-8-6-16(7-9-18)20-15-27-14-19(30-2)10-11-21(27)24(20)25(28)17-12-22(31-3)26(33-5)23(13-17)32-4/h6-15H,1-5H3 | 73 ± 4.5 | 64 ± 1.5 | 74 ± 2.0 | 880 ± 14 | 690 ± 156 | 800 ± 127 | > 50,000 | NT |
| **Paclitaxel** | InChI=1S/C47H51NO14/c1-25-31(60-43(56)36(52)35(28-16-10-7-11-17-28)48-41(54)29-18-12-8-13-19-29)23-47(57)40(61-42(55)30-20-14-9-15-21-30)38-45(6,32(51)22-33-46(38,24-58-33)62-27(3)50)39(53)37(59-26(2)49)34(25)44(47,4)5/h7-21,31-33,35-38,40,51-52,57H,22-24H2,1-6H3,(H,48,54)/t31-,32-,33+,35-,36+,37+,38-,40-,45+,46-,47+/m0/s1 | 54 ± 1.2 | 50 ± 1.0 | 46 ± 1.8 | 5 ± 1.4 | 2 ± 0.1 | 4 ± 1.1 | NT | 8.2 |
| **Verubulin** | InChI=1S/C17H17N3O/c1-12-18-16-7-5-4-6-15(16)17(19-12)20(2)13-8-10-14(21-3)11-9-13/h4-11H,1-3H3InChI=1S/C17H17N3O/c1-12-18-16-7-5-4-6-15(16)17(19-12)20(2)13-8-10-14(21-3)11-9-13/h4-11H,1-3H3 | NT | | | 3 ± 0.4 | 2 ± 0.3 | 5.0 ± 0.7 | NT | 3.2 |
| **BPR0L075** | InChI=1S/C19H19NO5/c1-22-12-5-6-13-14(10-20-15(13)9-12)18(21)11-7-16(23-2)19(25-4)17(8-11)24-3/h5-10,20H,1-4H3 | NT | | | 48 ± 16 | 3 ± 0.6 | 45 ± 7 | NT | NT |

^a^ Cell viability was assessed using the resazurin assay after 72 hours of compound treatment. Fluorescence was measured to estimate % of cellular growth inhibition at 50 µM values, based on triplicate experiments.

^b^ IC_50_ values were determined using the same resazurin-based assay, with fluorescence measurements performed after 72 hours of treatment. These experiments were carried out in triplicate.

^c^ Tubulin polymerization was evaluated using a commercial assay kit, following standard procedures. Compounds were incubated with tubulin, and fluorescence was measured over time to generate kinetic curves and determine the maximum polymerization rate (Vmax). These experiments were carried out in duplicate.

NT - Not Tested
